# Inhibition of the Caveolin-1 pathway promotes apoptosis and overcomes pan-tyrosine kinase inhibitor resistance in hepatocellular carcinoma

**DOI:** 10.1038/s41419-025-07887-4

**Published:** 2025-07-25

**Authors:** Tasnuva D. Kabir, Samuel Beck, Lisa M. Stuart, Ji Li, Rui Hou, Peiwen Liu, Shelby Margolius, Claire Kim, Yu Suk Choi, Edward R. Bastow, Dianne J. Beveridge, Lisa Spalding, Ziyi Li, Florent Ginhoux, Pierce Chow, Michael Phillips, Andrew D. Redfern, on behalf of The Liver Cancer Collaborative, Janina E. E. Tirnitz-Parker, Michael C. Wallace, Louise N. Winteringham, Benjamin J. Dwyer, Gayatri Shirolkar, Sara Pasic, Larissa Dymond, Jonathan Tibballs, George C. Yeoh, Alistair Forrest, Andrew J. Woo, Ankur Sharma, Jacob George, Geoffrey McCaughan, Peter J. Leedman

**Affiliations:** 1 Harry Perkins Institute of Medical Research, QEII Medical Centre, Nedlands and Centre for Medical Research, The University of Western Australia, Crawley, WA Australia; 2 Department of Dermatology, Boston University School of Medicine & Boston Medical Center, Boston, MA USA; 3 School of Science, Zhejiang Sci-Tech University, Hangzhou, China; 4 School of Human Sciences, The University of Western Australia, Crawley, WA Australia; 5 Medical School, The University of Western Australia, Crawley, WA Australia; 6 Department of Medical Oncology, Fiona Stanley Hospital, Murdoch, WA Australia; 7 Shanghai Institute of Immunology, Department of Immunology and Microbiology, Shanghai Jiao Tong University School of Medicine, Shanghai, China; 8 Singapore Immunology Network (SIgN), Agency for Science, Technology and Research (A∗STAR), Singapore, Singapore; 9 Translational Immunology Institute, SingHealth Duke-NUS Academic Medical Centre, Singapore, Singapore; 10 National Cancer Center Singapore, Singapore, Singapore; 11 Centre for Precision Health, Edith Cowan University, Joondalup, WA Australia; 12 Translational Genomics Program, Garvan Institute of Medical Research, Darlinghurst, NSW Australia; 13 Storr Liver Centre, Westmead Institute for Medical Research, Westmead Hospital and University of Sydney, Sydney, NSW Australia; 14 Liver Injury and Cancer, Centenary Institute, Sydney, NSW Australia; 15 Curtin Medical Research Institute, Curtin University, Perth, WA Australia; 16 Department of Hepatology, Sir Charles Gairdner Hospital, Perth, WA Australia; 17 Medical Imaging Department, Sir Charles Gairdner Hospital, Perth, WA Australia

**Keywords:** Liver cancer, miRNAs

## Abstract

Resistance to multi-tyrosine kinase inhibitors (TKI) is a major clinical concern in advanced hepatocellular carcinoma (HCC). Herein, we aimed to uncover the mechanisms underlying pan-TKI resistance and to identify potential therapeutic targets. We used multiple TKI-resistant HCC cell lines to identify caveolin-1 (CAV1) as a key driver of therapeutic resistance. CAV1 downregulation induced apoptosis, inhibited metastasis and restored TKI sensitivity in both inherent and acquired TKI-resistant HCC cells. Mechanistically, in acquired TKI-resistant cells aberrant CAV1/STAT3/P70S6K signalling is required for their survival, motility, and invasiveness. CAV1 inhibition reduced expression of dormancy regulators E-cadherin, RAC1 and p21, enhanced cancer stemness markers, and disrupted downstream STAT3/P70S6K and AMPKα signalling pathways, prompting cancer cells to exit from dormancy and initiate autophagy-induced cell death. Furthermore, selective inhibition of AXL and FGFR4 downstream of the CAV1 pathway sensitized TKI-resistant cells to sorafenib and lenvatinib, respectively. In addition, microRNA-7-5p (miR-7) was identified as an endogenous regulator of CAV1; and miR-7’s inhibitory effect on CAV1 and FGFR4 suppressed the STAT3/P70S6K pathway, promoted autophagy and triggered apoptosis in lenvatinib-resistant cells. Combination therapy using either lenvatinib or sorafenib and selective CAV1 inhibitors (e.g., siCAV1/miR-7), or AXL/FGFR4 inhibitors (e.g., BGB324/BLU9931) effectively overcame pan-TKI resistance. In HCC patient datasets, elevated CAV1 mRNA was observed in sorafenib non-responders, and single cell RNA-sequencing of HCC patient tumours revealed a rare population of CAV1+ cancer cells associated with recurrence. High CAV1 expression was specific to HBV+ HCC patients and independently predicted poor survival. Further, targeting of CAV1, AXL or FGFR4 effectively overcame TKI resistance in HCC patient derived organoids (PDOs). Our findings highlight a previously unrecognized role for CAV1-driven signalling in sustaining tumour dormancy, a critical and challenging therapeutic barrier underlying recurrence and pan-TKI resistance in HCC. Therapeutically targeting these pathways offer a promising and novel strategy to eliminate dormant tumour cells, thereby overcoming resistance and improving treatment outcomes.

## Introduction

Advanced hepatocellular carcinoma (HCC) is incurable. Patients invariably manifest therapeutic resistance, experience locoregional disease progression or metastasis, and succumb [1]. Achieving long-term survival therefore, remains challenging. Current first-line therapies for advanced HCC include multi-tyrosine kinase inhibitors (TKIs) such as sorafenib and lenvatinib, and combination therapy with the immunotherapy checkpoint inhibitors atezolizumab and bevacizumab. However, the efficacy of these therapies remains limited by resistance and adverse reactions [2]. TKIs in conjunction with checkpoint inhibitors and other therapies are currently being explored in clinical trials for advanced HCC (e.g., LEAP-002 and LEAP-012 studies). Primary endpoint analysis from the LEAP-002 trial showed no significant improvement in clinical outcome when HCC patients were co-treated with lenvatinib and Pembrolizumab [3]. Significantly, a meta-analysis of 1656 HCC patients from three randomised phase 3 clinical trials showed that the survival benefit of checkpoint inhibitors was restricted to patients with viral aetiology [4]. Furthermore, subsequent analysis of two smaller HCC cohorts revealed that median survival with immunotherapy was significantly worse in NASH-associated HCC compared to those with other etiologies, cohort 1: 5.4 versus 11 months and cohort 2: 8.8 vs 17.7 months, partly due to the abundance of dysfunctional T cells [4, 5]. TKIs still have a key place in the therapeutic landscape of HCC, as they remain the preferred choice for managing HCC in patients where immunotherapy is contraindicated, and in third-world countries where the costs of immunotherapy are excessive. Therefore, as TKIs play a major role in HCC treatment, further elucidation of the mechanisms of resistance and therapeutic action is warranted.

There is sparse data outlining ways to identify high-risk disease and potential recurrence during TKI therapy. Currently, high expression of c-kit and phospho-ERK in HCC tissues, and low plasma hepatocyte growth factor are used to predict sorafenib responders [6], whereas serum biomarkers for predicting lenvatinib responders include ST6GAL1 [7], c-reactive protein [8] and miRNA-3154 expression [9]. Notably, a 146-gene signature developed by Pinoyl et al. has shown promise in predicting recurrence risks in HCC patients [6]. Additionally, EGFR activation has been implicated in reducing lenvatinib efficacy [10–12], as supported by a small clinical trial where the combination of an EGFR inhibitor with lenvatinib elicited a dramatic anti-tumour response and reversed TKI resistance [12]. Recently, a comparative pharmacodynamic biomarker analysis from the phase III REFLECT trial revealed that both sorafenib and lenvatinib responders exhibit elevated VEGF levels, while lenvatinib responders also display increased serum FGF19 and FGF23 levels, underscoring its dual targeting of VEGFRs and FGFRs, unlike sorafenib [13]. Overall, there remains an urgent need to identify predictive markers of therapeutic response to TKIs and to develop strategies that overcome TKI resistance.

To understand the molecular drivers of pan-TKI resistance, and to identify potential therapeutic targets, we established multiple TKI-resistant (sorafenib and lenvatinib) human HCC cell lines. Using a comparative proteomics approach in HCC cells we identified TYRO3, a member of the TYRO3, AXL and MER (TAM) family of receptor tyrosine kinases (RTKs), as a driver of acquired sorafenib-resistance (SR) [14]. We have reported that a tumour suppressor microRNA-7-5p, miR-7, could inhibit the TYRO3/phospho-AKT pathway to overcome SR, and is therefore a potential HCC therapeutic [14–16]. In our current study, we used RNA-sequencing to comprehensively characterise the resistome of sorafenib-resistant Huh-7 sublines (Huh-7/SR1 and Huh-7/SR2; Huh-7/SR), and together with our lenvatinib-resistant (Huh-7/LR) HCC cells, we investigated in-depth which of these genes and pathways were essential for acquiring pan-TKI resistance.

We discovered that overexpression of caveolin-1 (CAV1), a component of lipid rafts, plays a central role in HCC relapse and in determining an individual’s response to both sorafenib and lenvatinib. Notably, inhibition of the CAV1 pathway using CAV1 targeting siCAV1/miR-7 or selective AXL/FGFR4 inhibitor, alone or in combination with sorafenib/lenvatinib, significantly reduced the viability, growth, and invasiveness of TKI-resistant HCC cells.

## Materials and methods

All materials and methods are described in detail in the supplementary section.

### Statistical analysis

All in vitro experiments were performed on three independent days with at least three technical repeats. For gene expression, the bars represent the average of relative target gene expression as fold changes ± standard deviation of one representative experiment. All numerical data is represented as average ± standard deviation. Statistical significance was determined by unpaired Student’s t-test, one-way ANOVA, RM-ANOVA, and two-way ANOVA as described under the Methods section using GraphPad Prism software (v8.3). Significance is indicated as *p < 0.05, **p < 0.01, ***p < 0.001 and ****p < 0.0001.

## Results

### Identification of CAV1 as a driver of pan-TKI resistance in HCC

**Fig. 1:**
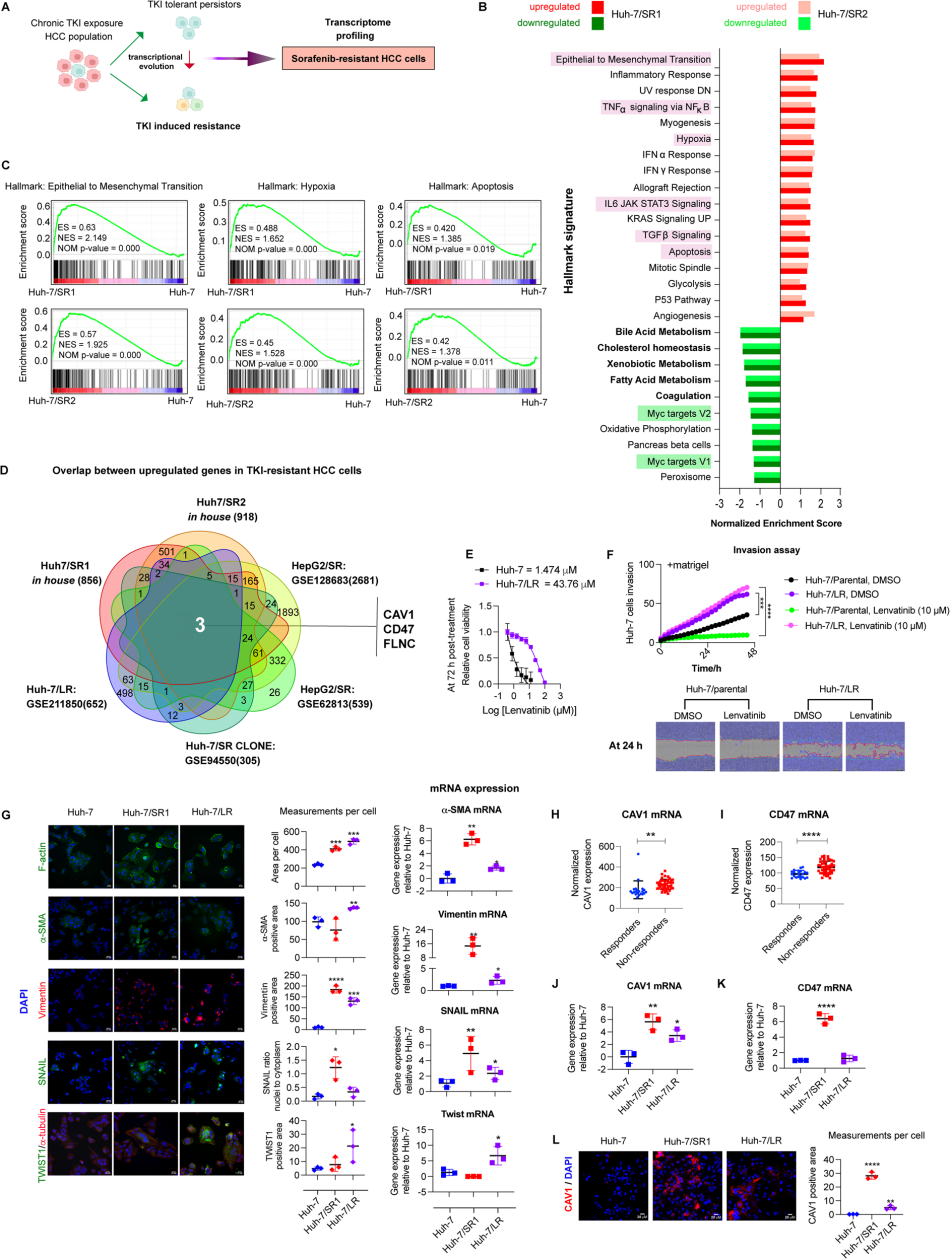
Discovery of CAV1 as a driver of pan-tyrosine kinase inhibitor resistance in HCC. **A** Experimental design. **B** Hallmark pathways up- and downregulated in sorafenib-resistant Huh-7/SR1 and Huh-7/SR2 cells (*n* = 3 per group). **C** Gene set enrichment plots showing enrichment score (ES), normalised enrichment score (NES) and nominal *p*-value (NOM *p*-value) for epithelial-to-mesenchymal transition (EMT), hypoxia and apoptosis hallmark pathways in Huh-7/SR1 and Huh-7/SR2 cells relative to parental Huh-7 cells. **D** Gene overlap analysis of upregulated genes in Huh-7/SR1, Huh-7/SR2, Huh-7 A7 clone (GSE94550), sorafenib-resistant HepG2 cells (GSE62813 and GSE128683) and lenvatinib-resistant Huh-7/LR cells (GSE211850). **E** Lenvatinib dose-response curve in Huh-7 and Huh-7/LR cells, as determined by cell titre assay (*n* = 3). **F** IncuCyte scratch assay evaluating lenvatinib’s effect on cell invasion through Matrigel in Huh-7 and Huh-7/LR cells (*n* = 3). **G** Validation of EMT markers (F-actin, *α*-smooth muscle actin (*α*-SMA), Vimentin, Snail and Twist1) in Huh-7/SR1 and Huh-7/LR cells using immunofluorescence immunocytochemistry and RT-qPCR (*n* = 3 per group). Expression levels of **H** CAV1 and **I** CD47 mRNA in sorafenib recipients from the Biostorm HCC cohort of STORM trial; responders (*n* = 21) vs non-responders (*n* = 46). RT-qPCR of **J** CAV1 and **K** CD47 expression in Huh-7/SR1 and Huh-7/LR cells (*n* = 3). **L** Immunofluorescence immunocytochemistry of CAV1 in Huh-7/LR cells (*n* = 3). Each experiment was performed on three independent days with at least three technical replicates. Error bars represent SD. Data were analysed by one-way ANOVA ( > 2 groups) and unpaired two-tailed student’s t-test. Significance is denoted as follows: *p < 0.05, **p < 0.01, ***p < 0.001, ****p < 0.0001.

To investigate the mechanisms of TKI resistance in HCC, we initially established sorafenib-resistant Huh-7 HCC cell lines (Huh-7/SR: Huh-7/SR1 and Huh-7/SR2) through serial passaging in increasing concentrations of sorafenib in the culture media (Fig. 1A). RNA-sequencing of Huh-7/SR cells identified 1922 differentially expressed genes (DEGs) between Huh-7/parental and its resistant derivatives, with 1,197 in Huh-7/SR1 and 1,296 in Huh-7/SR2 (Table S1, False Discovery Rate, FDR, of <0.01). Importantly, VEGFC was significantly elevated in Huh-7/SR1 cells, while FLT1 (VEGFR1) was upregulated in Huh-7/SR2, and both lines exhibited marked downregulation of FGF19. These findings are consistent with sorafenib’s selective inhibition of the VEGFR pathway and suggest compensatory mechanisms that may contribute to resistance (Suppl. Table S1).

Gene set enrichment analysis of the DEGs showed the hallmark signature pathways were significantly conserved in Huh-7/SR cells (Fig. 1B). Notably, we observed positive enrichment of epithelial to mesenchymal transition (EMT) and several related processes including TNFα/NFκB, IL6/JAK/STAT3 and TGFβ signalling, hypoxia, and apoptosis (Fig. 1B; pink highlights and 1 C). This enrichment suggests a shift towards an EMT phenotype in the Huh-7/SR cells, a process that also contributes to the evasion of apoptosis. In addition, processes related to cell proliferation were negatively enriched (e.g., Myc targets V2 and V1, Fig. 1B; green highlights). Conversely, we observed negative enrichment for hepatocyte-specific functional pathways, e.g., bile acid, xenobiotic, and fatty acid metabolism, and cholesterol homeostasis and coagulation pathways (Fig. 1B; in bold), suggesting sorafenib-resistant cells behave less hepatocyte-like than their parental counterparts. Gene Ontology term analysis confirmed that Huh-7/SR cells were enriched for biological processes, cellular components, and molecular functions associated with EMT (in bold) and dedifferentiation (in grey) (Suppl. Fig. S1A, B).

Comparative analysis of the DEGs from our in-house generated Huh-7/SR1 and Huh-7/SR2 cells with four external data sets; a sorafenib-resistant Huh-7 A7 clone (GSE94550 [17]), two sorafenib-resistant HepG2/SR lines (GSE62813 [18] and GSE128683 [19]), and an LR Huh-7/LR cell line (GSE211850 [20]) identified 22 upregulated and 1 downregulated gene common to sorafenib-resistant cells (Fig. S1C, D). However, only three genes, namely CAV1 (caveolin-1), CD47 (leucocyte surface antigen CD47) and FLNC (Filamin C), were consistently upregulated across all TKI-resistant datasets (Fig. 1D).

To evaluate the predictive value of the basal expression of these genes in defining TKI sensitivity and their potential as a de novo mechanism of pan-TKI resistance, we analysed a panel of 785 treatment-naïve human cancer cell lines from the Cancer Therapeutic Response Portal (v2.0). We evaluated the correlation coefficient between basal mRNA expression of 18,535 genes and their TKI sensitivity and ranked the genes accordingly (Suppl. Fig. S1E–H). We noted a significant positive correlation between TKI sensitivity and mRNA expression of well-established drug targets, e.g., FLT4, FLT3, and KIT, and designated these as “TKI responder” genes (Suppl. Fig. S1E–H, green labels, Suppl. Table S2). In contrast, 20/22 upregulated genes from the sorafenib-resistant cells signature, e.g., CAV1, AXL, FLNC, and CD47, exhibited a significant inverse correlation with sorafenib sensitivity, ranking them at the bottom of the correlation coefficient scores (Suppl. Fig. S1E; red labels, Suppl. Table S3). This inverse correlation suggests that a higher expression of these genes may serve as a predictive marker for poor therapeutic response to sorafenib, leading to their designation as “sorafenib non-responder” genes. Importantly, the high basal expression of CAV1, CD47 and FLNC was negatively correlated with sensitivity to other TKIs; lenvatinib, cabozantinib and regorafenib (in black; Suppl. Fig. S1F-H, Suppl. Table S3). These findings suggest that these genes may drive a common de novo mechanism of pan-TKI resistance across these cancer cell lines.

To validate these in silico findings, we established LR Huh-7/LR cells in a similar workflow as per Fig. 1A with the resistant cells having an EC50 of >40uM to lenvatinib (Fig. 1E). As compared to Huh-7 parental cells, the Huh-7/LR cells exhibited an increased migratory and invasive potential (Fig. 1F), and an elevated expression of the EMT markers *α*-SMA, Vimentin, Snail and Twist1, at both mRNA and protein levels (Fig. 1G). Additionally, TKI-resistant cells displayed significant upregulation of cancer stemness markers (CD44, Myc and KLF4) with Huh-7/LR cells enriched for EpCAM, Sox2, Nanog and CD133, while Huh-7/SR1 cells expressed higher CD24 but lower Nanog (Suppl. Fig. S2A).

To investigate the clinical relevance of CAV1, CD47 and FLNC we analysed the transcriptomic data from 67 HCC cases in the Biostorm cohort of the Storm trial, a phase 3 clinical study exploring sorafenib as an adjuvant therapy in liver cancer patients who had previously had local ablation surgery. The patients were categorised into responder and non-responder groups based on their response to sorafenib treatment and HCC recurrence (Suppl. Fig. S2B).

ROC curve analysis identified threshold geometric mean expression values of CAV1 ( > 163.9) and CD47 (>96.09), beyond which both genes demonstrated strong predictive value for therapeutic response. Specifically, CAV1 showed 93.4% sensitivity and 67% specificity, while CD47 exhibited 89.13% sensitivity and 52.38% specificity, with CAV1 emerging as the superior predictive marker (Fig. 1H, I & Suppl. Fig. S2C, D). Expression of FLNC was unchanged between the sorafenib responders and non-responders (Suppl. Fig. S2E) and was therefore not pursued further.

Further analysis of CAV1 and CD47 expression between sorafenib-resistant Huh-7/SR1 and LR Huh-7/LR cells demonstrated a significant upregulation of CAV1 in both lines, while CD47 was exclusively upregulated in Huh-7/SR1 cells, as compared to the parental cells (Fig. 1J–L).

Both tumour hypoxia and chemotherapy-induced autophagy are implicated in the acquisition of TKI-resistance [21]. Investigation of autophagy markers revealed Huh-7/SR cells had elevated phospho-AMPKα expression compared to parental Huh-7 (Suppl. Fig. S2F). Both Huh-7/SR1 and Huh-7/LR cells had increased lysosomal content, as indicated by lysotracker Deep Red staining (Suppl. Fig. S2G). Evaluation of autophagic flux showed parental Huh-7 cells expressed significantly higher levels of P62, LC3A/B (LC3) II/I, and glycosylated LAMP1 than Huh-7/SR1 and Huh-7/LR cells (Suppl. Fig. S2H, I). Both sorafenib and lenvatinib treatment induced LC3 II and P62 expression while reducing glycosylated LAMP1 compared to DMSO controls. Autophagy blockade with chloroquine (CQ) led to a significant accumulation of LC3 II, P62, and glycosylated LAMP1 across all conditions. Since P62 is a substrate for autophagy and LC3 II (lipidated form of LC3 I) and the glycosylated LAMP1 serve as autophagosome markers that are typically degraded during active autophagy, these findings suggest that parental Huh-7 cells exhibit low basal autophagic flux, reflected by high p62 levels, whereas TKI-resistant cells engage in active autophagy to counteract therapy-induced metabolic stress.

Collectively, our findings suggest CAV1-driven pathways and autophagy may play pivotal roles in mediating resistance to both sorafenib and lenvatinib, and therefore, selective inhibition of these pathways could potentially hinder HCC progression, overcome TKI resistance, and mitigate the risk of recurrence in HCC patients.

### CAV1 drives pan-tyrosine kinase inhibitor resistance by regulating survival, apoptosis and cancer cell motility

Given CAV1’s role as a mechanosensor in epithelial cells [22], its contribution to cancer cell stemness [23] and its promotion of HCC metastasis via autophagy inhibition [24–29], we investigated its impact on TKI resistance and EMT.

**Fig. 2:**
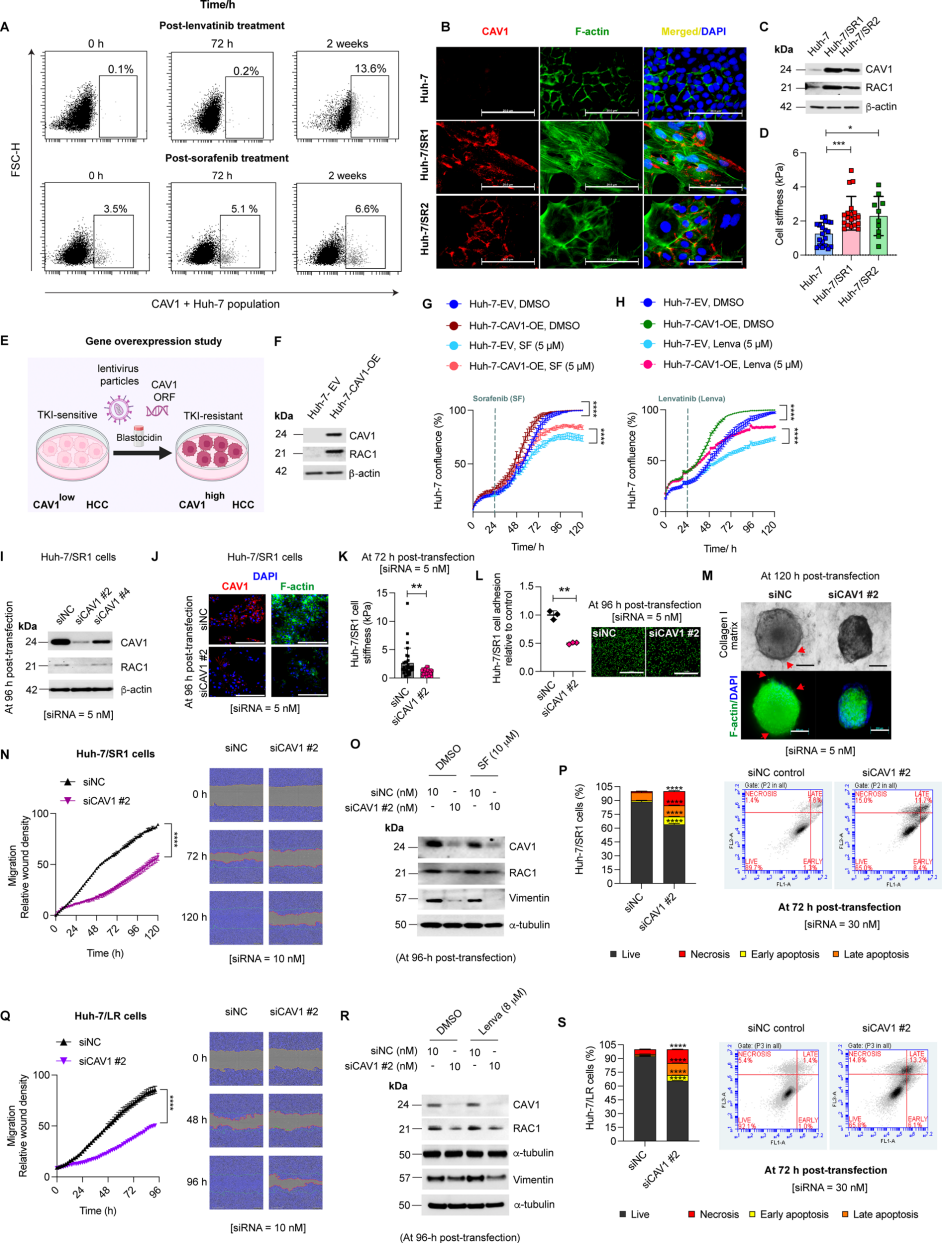
CAV1 regulates survival, motility and apoptosis in TKI-resistant HCC cells via modulating RAC1 and vimentin. **A** FACS analysis showing the proportion of CAV1+cancer cells in Huh-7 after chronic treatment with lenvatinib and sorafenib (*n* = 3 per group). **B** Immunofluorescence immunocytochemistry of CAV1 and F-actin in Huh-7, Huh-7/SR1 and Huh-7/SR2 cells (*n* = 3). **C** Western blot analysis of CAV1 and RAC1 in parental vs. sorafenib-resistant Huh-7/SR cells (*n* = 3). **D** Measurement of cell stiffness of Huh-7 cells and its sorafenib-resistant derivatives using atomic force microscopy (AFM) (*n* = 3, each dot represents one individual cell). **E** Schematics of gene overexpression study. **F** Western blot confirming CAV1 overexpression (OE) in Huh-7 cells; Huh-7-CAV1-OE, using a lentivirus overexpression system compared to empty virus (EV) infected control cells; Huh-7-EV. Growth curve showing the effect of CAV1 overexpression on proliferation of Huh-7 cells (Huh-7-CAV1-OE) and their susceptibility to **G** sorafenib and **H** lenvatinib, assessed by IncuCyte Zoom (*n* = 3). **I** Validation of CAV1 knockdown in Huh-7/SR1 cells and its impact on RAC1 by Western blot (*n* = 3). siNC group served as control. **J** Immunofluorescence for CAV1 and F-actin in CAV1 knockdown Huh-7/SR1 cells (*n* = 3). **K** Effect of CAV1 knockdown on Huh-7/SR1 cell stiffness assessed by AFM (*n* = 3). Evaluation of functional effects of siCAV1 in Huh-7/SR1 cells using: **L** cell adhesion assay (*n* = 3) and **M** 3D-invasion assay in collagen I gel. F-actin staining was done to visualise sprouting in collagen I gel (*n* = 3). **N** Migration of CAV1 knockdown Huh-7/SR1 cells assessed by IncuCyte Zoom scratch assay (*n* = 3). **O** Western blot for RAC1 and vimentin in Huh-7/SR1 cells treated with siCAV1 alone or in combination with DMSO vehicle/sorafenib (*n* = 3). **P** Apoptosis analysis in CAV1 knockdown Huh-7/SR1 cells measured by Annexin V-FITC apoptosis assay (*n* = 3. **Q** Migration of CAV1 knockdown Huh-7/LR cells assessed by IncuCyte Zoom scratch assay (*n* = 3). **R** Western blot for RAC1 and vimentin in Huh-7/LR cells treated with siCAV1 alone or in combination with DMSO vehicle/lenvatinib (*n* = 3). **S** Apoptosis analysis in CAV1 knockdown Huh-7/LR cells using Annexin V-FITC apoptosis assay. *β*-actin and *α*-tubulin were used as loading controls (*n* = 3). Each experiment was performed on three independent days with at least three technical replicates. Error bars represent ± SD. All growth curves and time course studies were analysed by one-way repetitive measure ANOVA. All other data were analysed by unpaired two-tailed student’s t-test. Significance is denoted as follows: **p < 0.01, ***p < 0.001, ****p < 0.0001 SF = sorafenib, Lenva = lenvatinib.

We treated Huh-7 cells with either lenvatinib or sorafenib for 72 h or 2 weeks and found that prolonged TKI treatment increased the population of CAV1+ Huh-7 cells (Suppl. Fig. S3A, Fig. 2A). This suggests that during the development of TKI resistance, drug-tolerant CAV1+ cancer cells are selectively enriched.

Analysis of Huh-7/SR cells revealed CAV1 expression was predominantly localised to the membrane and was associated with elevated F-actin and RAC1 expression (Fig. 2B, C), and an increase in cellular stiffness, as measured by atomic force microscopy (Fig. 2D). These findings were corroborated and independently validated by generating two additional sorafenib-resistant HCC cell lines from Hep3B cells: Hep3B/SR1 and Hep3B/SR2, both of which showed an increased expression of CAV1, F-actin, and RAC1 as compared to parental cells (Suppl. Fig. S3B–D).

We next assessed CAV1 levels across a panel of well-differentiated, metastatic, and moderately-to-poorly differentiated HCC cell lines [30, 31]. Clinically aggressive (metastatic/moderately-to-poorly differentiated) HCC cells exhibited significantly higher CAV1 expression and lower albumin and E-cadherin levels compared to well-differentiated tumour cell lines (Suppl. Fig. S3E). Notably, the more aggressive cell lines originated from HBV+ HCC, demonstrated inherent resistance to both sorafenib and lenvatinib, as indicated by their higher EC_50_ values (Suppl. Fig. S3F). This association between CAV1 overexpression in HBV+ HCC cell lines and inherent TKI resistance was observed exclusively in moderately-to-poorly differentiated HCC, as CAV1 expression in the well-differentiated HBV+ cell lines Hep3B [30] and PLC-PRF-5 [30] was negligible (Suppl. Fig. S3E-F).

To test the hypothesis that CAV1 drives resistance to both sorafenib and lenvatinib in HCC, we used a lentivirus transduction system to stably overexpress CAV1 in TKI-sensitive parental Huh-7 cells (Huh-7-CAV1-OE, Fig. 2E). CAV1 overexpression in Huh-7-CAV1-OE cells induced RAC1 expression (Fig. 2F) and promoted survival under sorafenib and lenvatinib treatment (Fig. 2G, H). Immunocytochemistry confirmed survival advantages in CAV1-overexpressing cells under drug treatment conditions (Suppl. Fig. S3G), consistent with increased viability observed in ATP-based assays (Suppl. Fig. S3H). To delineate whether this increased survival resulted from altered proliferation or apoptosis, we analysed ki67 and cleaved-caspase 3 (c-CASP3) expression. DAPI staining showed that cell counts aligned with growth curves and viability assays (Suppl. Fig. S3I). Although both TKIs retained their anti-proliferative effects, CAV1-overexpressing cells evaded apoptosis, as evidenced by the absence or marked reduction of c-CASP-3-positive cells, which were detected only in TKI-treated Huh-7-EV cells (Suppl. Fig. S3I). These findings highlight CAV1 facilitates TKI resistance by enabling apoptosis evasion.

To further explore the role of CAV1 inhibition on TKI resistance, we used specific siRNAs (siCAV1) to knockdown CAV1 expression in Huh-7/SR1 cells (Suppl. Fig. S4A, B), and observed a significant reduction of RAC1 expression, F-actin levels, and cellular stiffness in the cells with reduced CAV1 (Fig. 2I–K). These cells also demonstrated decreased adhesion to fibronectin and reduced invasion in collagen I matrix (Fig. 2L, M). Moreover, CAV1 downregulation significantly impaired migration and invasion of Huh-7/SR1 in a scratch assay (Fig. 2N, Suppl. Fig. S4C) and chemotaxis in 2D transwell assays (Suppl. Fig. S4D) accompanied by decreased RAC1 and vimentin expression (Fig. 2O). Similar effects were observed in Huh-7/LR (Fig. 2Q, R, Suppl. Fig. S4E) and Huh-7/SR2 cells with siRNA-mediated CAV1 knockdown (Suppl. Fig. S4F-G), and in inherently TKI-resistant cell lines (SNU475 and SNU499) following shRNA-mediated depletion of CAV1 expression (Suppl. Fig. S4H–K).

Given the role of CAV1 overexpression in apoptosis evasion, we investigated apoptosis and cell cycle dynamics in siRNA-mediated CAV1 knockdown Huh-7/SR1 cells. CAV1 depletion significantly increased apoptosis (Fig. 2P) and induced cell cycle exit from G0/G1-phase into S-phase, evidenced by increased ki67 staining (Suppl. Fig. S4L, M). These cell cycle alterations strongly suggest an escape from cellular dormancy. Consistent with this observation, qRT-PCR analysis revealed CAV1 depletion markedly elevated cancer stemness markers in Huh-7/SR1 cells (Suppl. Fig. S4N, O), further supporting the exit from dormancy and reactivation. While Slug expression was increased, its downstream targets, vimentin and ITGβ3, were reduced (Suppl. Fig. S4P). Additionally, CAV1 knockdown disrupted G2/M checkpoint gene expression (Suppl. Fig. S4Q) and elevated ENO2 mRNA, a key glycolytic enzyme (Suppl. Fig. S4R, left panel). Interestingly, despite increased ENO2 expression, ATP production assays indicated a reduction in glycolysis in CAV1-depleted Huh-7/SR1 cells (Suppl. Fig. S4R, right panel). These findings suggest that CAV1 promotes TKI resistance in HCC partly via maintaining tumour dormancy. Sudden depletion of CAV1 triggers metabolic stress, driving dormant cells into apoptosis and compromising their capacity to re-enter the cell cycle. This apoptotic effect of CAV1 depletion was also validated in Huh-7/LR cells (Fig. 2S).

Collectively, these data support CAV1 as a key regulator of survival, apoptosis resistance, and metastatic potential in TKI resistant cells, mediated in part by its action on RAC1 and vimentin, thereby contributing to the development of pan-TKI resistance.

### CAV1 knockdown sensitises HCC cells to low doses of sorafenib and lenvatinib

**Fig. 3:**
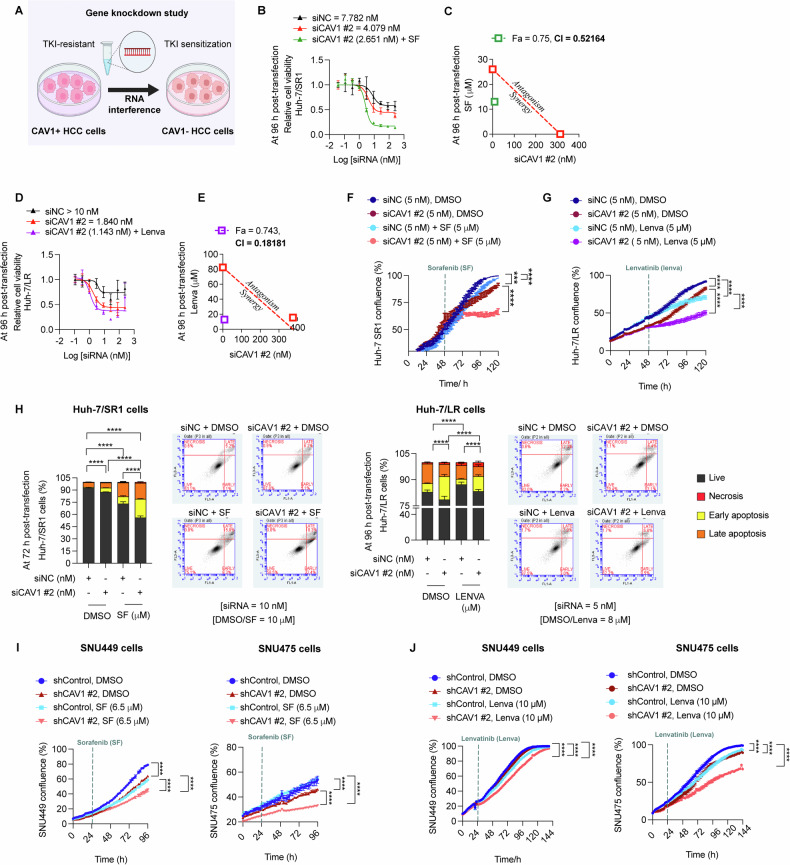
Downregulation of CAV1 restores sensitivity of TKI-resistant HCC cells to sorafenib and lenvatinib. **A** Experimental design schematic. **B** Dose-response curve of siCAV1 alone and in combination with sorafenib (SF) in Huh-7/SR1 cells (*n* = 3). siNC treatment group served as control. **C** Combination index (CI) and isobologram analysis for sorafenib and siCAV1, demonstrating synergy (F_a_ = 0.75, CI = 0.5216) (*n* = 3). **D** Dose-response curve of siCAV1 alone and in combination with lenvatinib (lenva) in LR cells (*n* = 3). siNC treatment group served as control. **E** Combination index (CI) and isobologram analysis for lenvatinib and siCAV1 in Huh-7/LR cells, demonstrating strong synergy (F_a_ = 0.743, CI = 0.18181) (*n* = 3). **(F)** Validation of synergy between siCAV1 #2 and sorafenib in Huh-7/SR1 cells by IncuCyte growth curve assays (*n* = 3). **G** Validation of synergy between siCAV1 #2 and lenvatinib in Huh-7/LR cells by IncuCyte growth curve assays (*n* = 3). **H** Annexin V-FITC apoptosis assay to measure the effect of combining siCAV1 #2 and sorafenib/lenvatinib on Huh-7/SR1 and Huh-7/LR cell death (*n* = 3). Growth curves illustrating the impact of shRNA-mediated CAV1 down-regulation in primary TKI-resistant HCC cell lines SNU449 and SNU475 on sensitivity to **I** sorafenib and **J** lenvatinib. For all drug treatment groups, DMSO vehicle served as control. Each experiment was performed on three independent days with at least three technical replicates. Error bars represent ± SD. All growth curves and time course studies were analysed by one-way repeated measures ANOVA. All other data were analysed by one-way ANOVA with multiple comparison. Significance is denoted as follows: ***p < 0.001, ****p < 0.0001. SF = sorafenib, Lenva = Lenvatinib.

We next investigated whether CAV1 knockdown could sensitise TKI-resistant HCC cells to low doses of TKIs (Fig. 3A). In acquired TKI-resistant cells, siCAV1 alone effectively inhibited viability of both Huh-7/SR1 and Huh-7/LR cells in a dose-dependent manner (Fig. 3B, D). Furthermore, siCAV1 synergised with both sorafenib and lenvatinib, as indicated by a combination index (CI) value < 1 on the isobolograms (Fig. 3C, E). Time-lapse microscopy confirmed this synergy in Huh-7/SR1 and Huh-7/LR cells (Fig. 3F, G).

To determine the mechanisms underlying this synergy, we performed apoptosis and survival analyses of Huh-7/SR1 and Huh-7/LR cells treated with or without siCAV1 in combination with sorafenib or lenvatinib, respectively. Our findings showed Huh-7/LR cells were more sensitive to very low doses of siCAV1 compared to Huh-7/SR1 cells (5 nM vs 10 nM, Fig. 3H). In Huh-7/SR1 cells, siCAV1 alone increased both early and late apoptotic cells, which was significantly enhanced in the presence of sorafenib (Fig. 3H, left panel). In contrast, in Huh-7/LR cells, siCAV1 alone caused a significant increase in early apoptotic cells. Interestingly, treatment with lenvatinib in siRNA control cells promoted survival in Huh-7/LR cells, leading to an increase in the percentage of live cells and a reduction in apoptotic cells. However, co-treatment with siCAV1 and lenvatinib abolished the survival benefit conferred by lenvatinib (Fig. 3H, right panel). These findings were also validated by cell counts, as measured by immunocytochemistry staining, to assess the overall cell survival in both Huh-7/SR1 and Huh-7/LR cells (Suppl. Fig. S5A-F).

Similarly, shRNA-mediated stable knockdown of CAV1 in the primary TKI-resistant SNU449 and SNU475 cells enhanced the cytotoxic effects of low-dose sorafenib, lenvatinib and cabozantinib (Fig. 3I-J, Suppl. Fig. S5G). Overall, these results underscore the critical role of CAV1 in regulating TKI sensitivity in HCC cells.

### CAV1 activates the P70S6K/STAT3 pathway via regulation of dormancy-associated factors E-cadherin, RAC1 and p21, and its inhibition promotes autophagy in acquired TKI-resistant cells

**Fig. 4:**
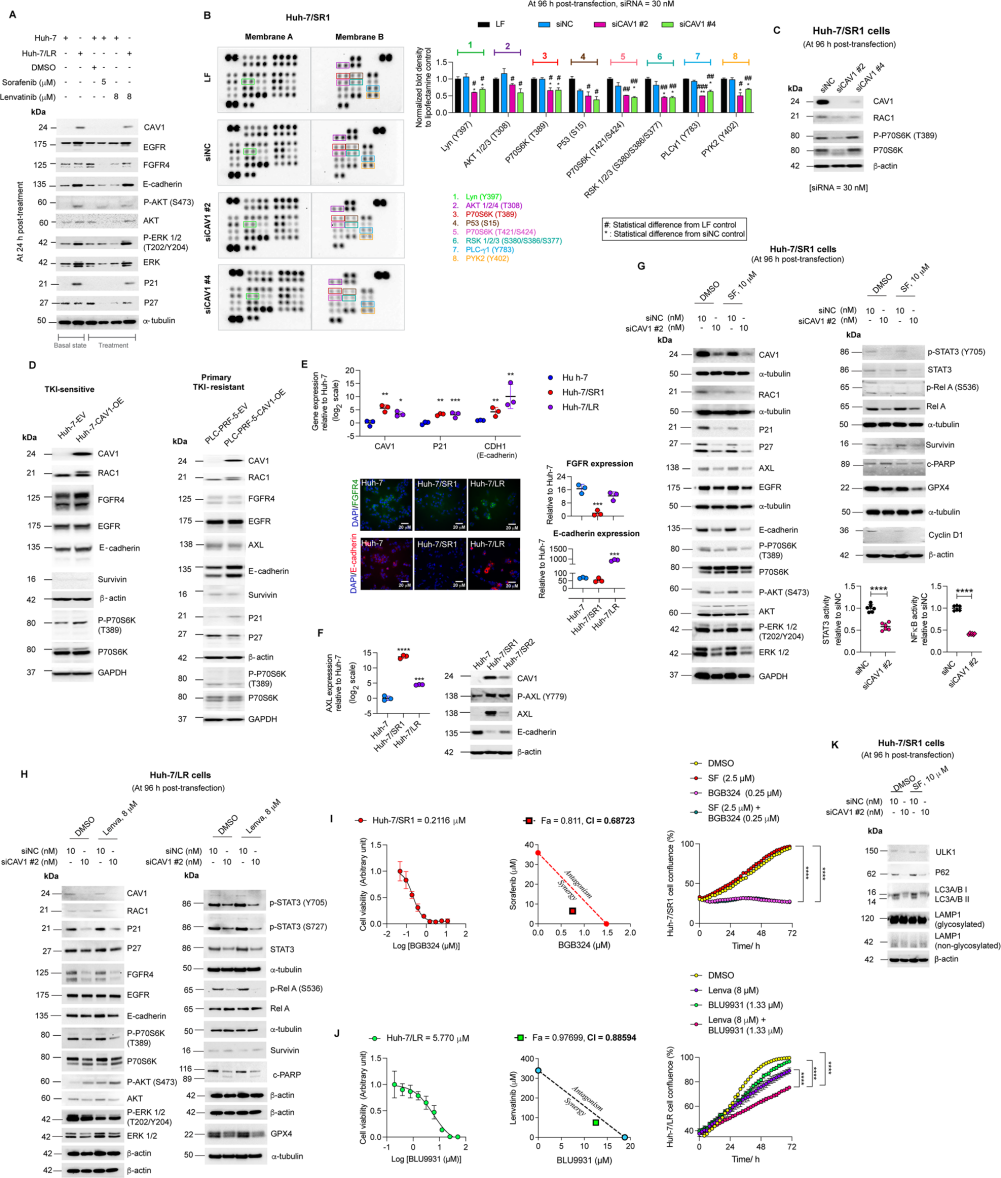
CAV1 regulates distinct molecular pathways in primary and acquired TKI resistance. **A** Western blot for key proteins (CAV1, EGFR, FGFR4, E-cadherin, p21 & p27) and pathways (AKT/ERK) in Huh-7 parental and Huh-7/LR cells under basal conditions and post-treatment (*n* = 3). **B** Pathway identification following CAV1 knockdown in Huh-7/SR1 cells using a proteome profiler array, quantified by band densitometry (*n* = 1). **C** Validation of P70S6K signalling in CAV1-depleted Huh-7/SR1 cells by Western blot (*n* = 3). **D** Western blot evaluation of pathways activated by CAV1 overexpression in TKI-naïve Huh-7 parental cells (Huh-7-CAV1-OE) and primary TKI-resistant PLC-PRF-5 cells (PLC-PRF-5-CAV1-OE) (*n* = 3). **E** RT-qPCR analysis of *CAV1*, *p21(CDKN1A)*, *CDH1* (E-cadherin) mRNA, with immunofluorescence detection of FGFR4 and E-cadherin in Huh-7, Huh-7/SR1 and Huh-7/LR cells (*n* = 3). **F** AXL profiling in Huh-7/SR cells by RT-qPCR and Western blot (*n* = 3). **G** Western blot validation of CAV1 dependence in CAV1-depleted Huh-7/SR1 cells treated with sorafenib or DMSO control, further confirmed by STAT3 and NFκB activity assays using luciferase reporters. **H** Western blot validation of CAV1 dependence in CAV1-depleted Huh-7/LR cells treated with lenvatinib or DMSO control. **I** Dose-response curve of AXL inhibitor; BGB324, in Huh-7/SR1 cells, assessed by cell viability assay, with synergy between sorafenib and BGB324 evaluated through combination index (CI) and isobologram analysis (Fa = 0.811, CI = 0.68723) and confirmed via IncuCyte growth curve assays (*n* = 3). **J** Dose-response curve of selective FGFR4 inhibitor; BLLU9931, in Huh-7/LR cells, with synergy between lenvatinib and BLU9931 assessed via CI and isobologram analysis (Fa = 0.97699, CI = 0.88594), and validated using IncuCyte growth curve assays (*n* = 3). **K** Western blot analysis for autophagy markers (P62, LC3A/B I (16 kDa), LC3A/B II (14 kDa), and LAMP1) in CAV1-depleted Huh-7/SR1 cells following sorafenib treatment. All experiments were performed on three independent days with at least three technical replicates. Error bars represent ± SD. Growth curves and time course studies were analysed by one-way repeated measure ANOVA, while all other data were evaluated by one-way ANOVA with multiple comparison ( > 2 groups) or an unpaired two-tailed student’s t test. Significance is denoted as follows: *p < 0.05, **p < 0.01, ***p < 0.001, ****p < 0.0001. CI = combination index, Fa = fractional inhibition, SF = sorafenib, Lenva = lenvatinib. *β*-actin and *α*-tubulin were used as loading controls.

In previous studies, we demonstrated that activation of the TYRO3/AKT signalling pathway contributes to sorafenib resistance in HCC [14]. Here, we compared the altered pathways between Huh-7 parental and Huh-7/LR cells and assessed their response to 24 h lenvatinib treatment (Fig. 4A) using sorafenib and DMSO as controls.

Under basal conditions, Huh-7/LR cells displayed elevated levels of CAV1, EGFR, E-cadherin and p21 compared to Huh-7 parental cells, along with activation of the downstream AKT and ERK pathways. Both cell lines showed comparable expressions of FGFR4 and p27 (Fig. 4A). Notably, in parental Huh-7 cells, 24 h lenvatinib treatment significantly reduced expression of its target, FGFR4, alongside E-cadherin and p27, and efficiently inhibited downstream ERK signalling compared to DMSO-treated controls. Consistent with previous findings, sorafenib, but not lenvatinib, activated the phospho-AKT pathway in Huh-7 parental cells (Fig. 4A). In contrast, lenvatinib-treated Huh-7/LR cells showed subtle changes in EGFR, FGFR4, CAV1, E-cadherin, p21, and p27 expression, while further activating the ERK pathway. These findings suggest that aberrant expression of these proteins may be advantageous and associated with acquired lenvatinib resistance.

Next, using a phosphokinase array, we found that CAV1 knockdown in Huh-7/SR1 cells suppressed signalling via the P70S6K, AKT, P53, RSK, PLCγ1, Lyn, and PYK2 pathways (Fig. 4B). Especially, no significant alterations in GSK3α/β and *β*-catenin levels were observed. Immunoblotting confirmed that CAV1 knockdown by siCAV1 #2 significantly inhibited the P70S6K pathway in Huh-7/SR1 cells (Fig. 4C). Interestingly, while lenvatinib inhibited the P70S6K pathway in both Huh-7 parental and Huh-7/LR cells, no difference in phosphorylated P70S6K levels was observed between the two cell types under basal or drug-treated conditions (Suppl. Fig. S6A).

We further investigated CAV1-mediated signalling, leveraging lentivirus-induced CAV1 overexpression models in TKI naïve Huh-7 (Huh-7-CAV1-OE) and inherently TKI-resistant PLC-PRF-5 (PLC-PRF-5-CAV1-OE) cells. In Huh-7-CAV1-OE cells, CAV1 overexpression significantly increased RAC1, FGFR4 and E-cadherin expression, activated the downstream P70S6K pathway (Fig. 4D, left panel), and promoted growth in 3D cultures (Suppl. Fig. S6B-C). In contrast, CAV1 overexpression in PLC-PRF-5-CAV1-OE cells increased RAC1, p21 and E-cadherin levels, but did not affect FGFR4 or the P70S6K levels, suggesting that FGFR4/P70S6K signalling may be specific to acquired TKI resistance (Fig. 4D, right panel and Suppl. Fig. S6D). Importantly, survivin, a marker of β-catenin activation in HCC, remained unchanged (Fig. 4D).

We next examined E-cadherin (CDH1) and p21 expression in Huh-7/SR1 and Huh-7/LR cells, finding both significantly elevated at mRNA levels compared to parental Huh-7 cells, although E-cadherin protein elevation was exclusive to Huh-7/LR cells (Fig. 4E, F). Additionally, we investigated EGFR and FGFR4 expression and found that while EGFR was significantly upregulated in both cell lines, FGFR4 expression remained unchanged in Huh-7/LR cells but was significantly downregulated at the protein level in Huh-7/SR1 (Suppl. Fig. S6E). To identify the growth factor receptor tyrosine kinase modulating survival via the CAV1 pathway in sorafenib-resistant cells, we examined AXL expression in Huh-7/SR cells, as indicated by RNA-seq data. AXL expression was significantly elevated in both Huh-7/SR1 and Huh-7/SR2 cells at both mRNA and protein levels (Fig. 4F). These findings suggest a link between AXL and sorafenib resistance, and FGFR4 and lenvatinib resistance.

Next, we explored the impact of CAV1 inhibition in Huh-7/SR1 cells. CAV1 knockdown in Huh-7/SR1 cells significantly reduced dormancy regulators RAC1, p21, p27, AXL, EGFR, and E-cadherin expression, along with decreased survival signalling via the P70S6K, AKT, ERK, STAT3, and NFκB pathways and suppressed levels of survivin, GPX4, and cyclin D1 (Fig. 4G). These effects were further amplified by sorafenib treatment. A similar patten was observed in Huh-7/LR cells, except for the AKT pathway, which was induced, potentially as a compensatory mechanism (Fig. 4H). Notably, ERK inhibition by CAV1 knockdown was limited to Huh-7/SR1 cells. Additionally, CAV1 inhibition in Huh-7/LR cells and inherently TKI-resistant SNU475 and SNU449 cells significantly downregulated FGFR4, implicating its role in lenvatinib resistance (Fig. 4H, Suppl. Fig. S6F, G). In inherently TKI-resistant cells, CAV1 depletion also reduced RAC1, p21 and p27 levels, inhibited the AKT pathway in SNU475 cells, and suppressed the STAT3 pathway in SNU449 cells, with negligible effect on P70S6K signalling. These findings suggest that P70S6K activation is specific to acquired TKI resistance (Suppl. Fig. S6F, G).

To investigate AXL’s role in HCC, we used selective siRNAs (siAXL #45 and #47, Suppl. Fig. S7A, B), which significantly reduced AXL expression at both mRNA and protein levels. AXL depletion selectively impaired Huh-7/SR1 cells growth but had no impact on AXL-null parental Huh-7 cells (Suppl. Fig. S7C). Knockdown of AXL restored sorafenib sensitivity in Huh-7/SR1 cells, as shown by IncuCyte growth assays (Suppl. Fig. S7D) and ATP-based viability assays (Suppl. Fig. S7E). Additionally, AXL depletion reduced invasiveness and chemotaxis of Huh-7/SR1 cells, confirmed by IncuCyte scratch and 2D transwell assays (Suppl. Fig. S7F, G).

Cell cycle analysis revealed that AXL downregulation decreased the G0/G1 population and increased G2/M-phase entry (Suppl. Fig. S7H). This effect was amplified by sorafenib, which further increased the proportion of G2/M-phase cells while reducing those in S-phase (Suppl. Fig. S7H). Apoptosis assays showed that AXL-depleted, sorafenib-treated Huh-7/SR1 cells exhibited significantly higher early and late apoptosis (Suppl. Fig. S7I). Immunocytochemistry confirmed these findings, as AXL knockdown increased both ki67 and c-CASP3 positive cells, while sorafenib reduced ki67 staining but sustained apoptotic cell populations (Suppl. Fig. S7J).

Pathway analysis revealed that AXL depletion inhibited its phosphorylation at Y779 residue, reducing downstream STAT3 activation (Suppl. Fig. S8A). Other survival signalling molecules (e.g., phospho-AKT, -ERK and -Rel, p21, and p27) showed variable responses, while CAV1 levels remained unchanged, supporting the hypothesis that AXL functions downstream of CAV1 (Suppl. Fig. S8A). In the combination treatment group, phospho-c-JUN signalling was also inhibited by siAXL and sorafenib (Suppl. Fig. S8B).

To validate these findings, we treated Huh-7/SR1 cells with BGB324, a selective phospho-AXL inhibitor. Western blot and ELISA confirmed its specificity, as it only inhibited the naïve form of phospho-AXL (Suppl. Fig. S8C, D). Like siAXL, BGB324 (4 h treatment) suppressed STAT3 and c-JUN signalling when combined with sorafenib, suggesting these pathways are AXL-dependent. Huh-7/SR1 cells were highly sensitive to BGB324 (EC50 of 0.212 μM) (Fig. 4I, left panel). BGB324 synergised with sorafenib (Fig. 4I, middle panel), but its cytotoxicity was independent of AXL expression, inhibiting both AXL-negative Huh-7 parental cells and AXL-positive SNU475 and SNU449 equally (Suppl. Fig. S8E–G). Time-lapse microscopy confirmed BGB324 as a potent single agent that did not further enhance sorafenib cytotoxicity in Huh-7/SR1 cells (Fig. 4I, right panel). Notably, differences in AXL-targeting siRNA efficacy were observed, with siAXL #47 performing better in well-differentiated AXL + PLC-PRF-5 cancer cells, while siAXL #45 was more effective in AXL+ high-grade SNU449 cells (Suppl. Fig. S8H, I).

We identified BLU9931 as a selective FGFR4 inhibitor (Fig. 4J and Suppl. Fig. S9A–C). Huh-7/SR1 cells, with low basal FGFR4 expression, exhibited a higher EC50 (13.57 μM) whereas FGFR4-expressing Huh-7/LR cells (EC_50_ = 5.77 μM), were more sensitive. Analysis of isobolograms showed BLU9931 synergised with lenvatinib, overcoming lenvatinib resistance in Huh-7/LR cells (Fig. 4J, middle and right panel).

Next, we explored the effect of CAV1 and AXL knockdown on autophagy in Huh-7/SR1 cells. CAV1 knockdown significantly reduced LC3 II/I ratios, with minimal alterations in P62 levels, that increased upon CQ treatment (Suppl. Fig. S10A, left panel). Importantly, CAV1-depleted cells accumulated higher levels of P62, indicative of induced autophagy (Suppl. Fig. S10A, left panel), which remained unaffected by sorafenib treatment (Fig. 4K). Mechanistically, CAV1 depletion decreased phospho-AMPKα activity, as evidenced by reduced phosphorylation of its downstream target acetyl-Co A carboxylase (Suppl. Fig. S10A, right panel). Conversely, Huh-7-CAV-OE cells exhibited elevated phospho-AMPKα activity (Suppl. Fig. S10B), suggesting CAV1 regulates AMPKα activity in TKI resistance. Lysotracker staining and lysosomal enzymatic activity assays confirmed that CAV1 knockdown increased lysosome numbers and heightened activity in Huh-7/SR1 cells, further accentuated by sorafenib treatment (Suppl. Fig. S10C, D).

AXL knockdown elicited similar effects, significantly reducing P62 and LC3 II/I ratios, indicative of autophagy induction (Suppl. Fig. S10E). CQ treatment increased both markers. In AXL-depleted cells, sorafenib further enhanced autophagy, evidenced by increased ULK1 and ATG7 expression- key regulators of autophagy initiation and execution (Suppl. Fig. S10F). Additionally, like CAV1 knockdown (Fig. 4G), AXL depletion induced c-PARP expression and reduced survival molecules such as cyclin D1 (Suppl. Fig. S10G), cell cycle regulators; p21 and p27 (Suppl. Fig. S10G), and phospho-AMPKα activity (Suppl. Fig. S10H). Lysotracker and DQ Red BSA assays confirmed increased lysosome numbers and enhanced lysosomal activity following AXL knockdown, mirroring the effects observed with siCAV1 (Suppl. Fig. S10I, J).

To evaluate the therapeutic potential of co-targeting CAV1 and AXL, we performed IncuCyte growth assays on SNU449 cells following dual knockout of AXL and CAV1 using siRNAs. This co-inhibition significantly reduced cell growth and restored sorafenib sensitivity (Suppl. Fig. S10K), reinforcing our hypothesis that CAV1 and AXL are key therapeutic targets in sorafenib resistance.

Collectively, these findings identify CAV1 as a central regulator of tumour dormancy-associated pathways and acquired TKI-resistance through modulation of E-cadherin, RAC1 and p21, alongside activation of the P70S6K and STAT3 pathways. CAV1 mediates sorafenib resistance through AXL signalling and lenvatinib resistance via FGFR4 signalling. In AXL-positive, inherently TKI-resistant cells lacking E-cadherin, CAV1 primarily modulates the AKT and STAT3 pathway, suggesting that P70S6K activation may partially depend on E-cadherin. Both AXL and CAV1 regulate autophagy, further contributing to TKI resistance. Overall, our findings position CAV1, AXL, and FGFR4 as promising therapeutic targets for overcoming pan-TKI resistance in HCC.

### miR-7 is an endogenous regulator of CAV1

**Fig. 5:**
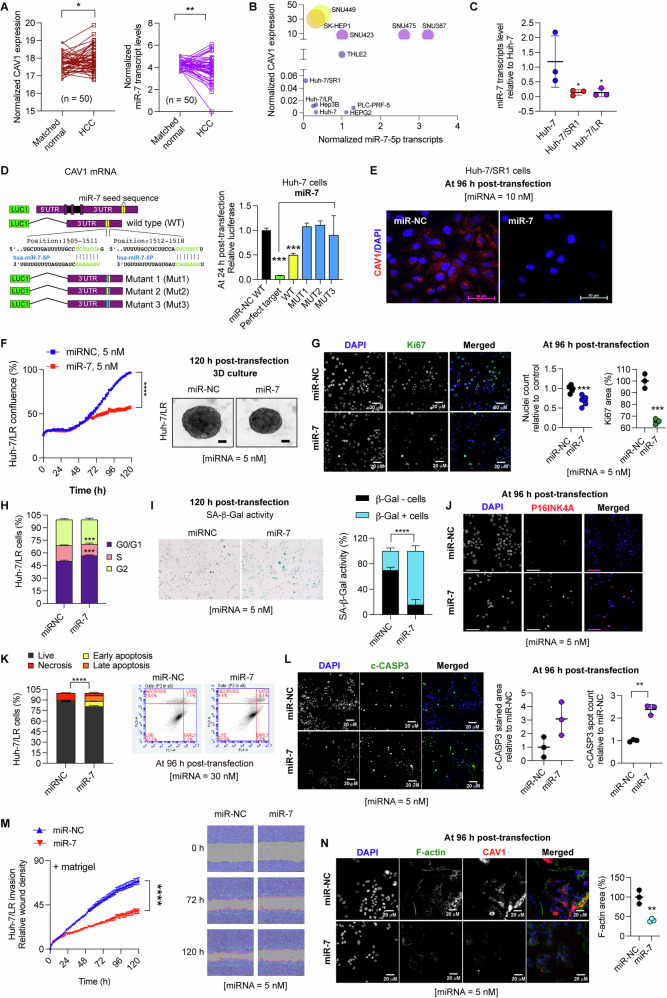
miR-7 functions as an endogenous inhibitor of CAV1. **A** Expression profile of CAV1 and miR-7 transcripts in the TCGA HCC cohort (tumour versus matched normal, *n* = 50). **B** RT-qPCR analysis of CAV1 and miR-7 levels in the HCC cell panel, normalised to a normal-like hepatocyte cell line, THLE2 (*n* = 3). **C** RT-qPCR measurement of miR-7 expression in acquired TKI-resistant Huh-7 cells (*n* = 3). **D** Schematic representation of predicted miR-7 seed-sequences in the 3’UTR of human CAV1 mRNA (TargetScan Human v7.1), with wild-type and mutant constructs used in dual-luciferase reporter assays in Huh-7 cells to assess the effect of miR-7 on CAV1 transcription. **E** Immunofluorescence detection of CAV1 protein in miR-7 transfected Huh-7/SR1 cells. **F–N** Assessment of miR-7’s anti-cancer effects on Huh-7/LR cells compared to miR-NC control: **F** Effect on growth using IncuCyte growth curve assay and 3D cultures (*n* = 3). **G** Effect on cell proliferation measured by immunofluorescence staining for ki67 (*n* = 3). **H** Effect on cell cycle (*n* = 3). Effect on senescence measured by **I** senescence associated *β*-galactosidase (SA-β-gal) activity (*n* = 3) and **J** immunofluorescence for p16 staining (*n* = 3). Effect on cell death measured by **K** Annexin V-FITC apoptosis assay (*n* = 3) and **L** immunofluorescence for c-CASP3 staining (*n* = 3). **M** Effect on Huh-7/LR cell invasion in Matrigel via IncuCyte Zoom scratch assay (*n* = 3). **N** Validation of the effect of miR-7 on CAV1 (red) and F-actin (green) expression using immunofluorescence staining (*n* = 3). All experiments were performed on three independent days with at least three technical replicates. Error bars represent ± SD. Growth curves and time course studies were analysed by one-way repetitive measure ANOVA., while all other data were evaluated by an unpaired two-tailed student’s t test. Significance is denoted as follows: *p < 0.05, **p < 0.01, ***p < 0.001, ****p < 0.0001.

To explore the intrinsic regulatory mechanisms of CAV1 in acquired TKI-resistant HCC cells, we utilised the TargetScan Human database (v7.1) and identified two putative miR-7 seed sequences in the CAV1 3’UTR. Analysis of the TCGA HCC cohort demonstrated that increased CAV1 mRNA levels were inversely correlated with miR-7 expression in matched HCC tissues (Fig. 5A). Consistently, aggressive HCC cell lines with high CAV1 expression exhibited negligible miR-7 levels, whereas those with retained miR-7 expression showed comparatively lower CAV1 level, suggesting a regulatory role for miR-7 (Fig. 5B).

Further analysis revealed that both sorafenib- and LR Huh-7 cells expressed significantly reduced levels of miR-7 transcripts (Fig. 5C), accompanied by elevated levels of miR-7 target genes, including EGFR, TYRO3, CAV1 and IL-8 in Huh-7/LR cells (Suppl. Fig. S11A). Treatment with miR-7, but not an anti-miR-7, significantly reduced the expression of these mRNA targets in Huh-7/LR cells (Suppl. Fig. S11B, C). Next, a luciferase reporter construct containing CAV1 wild-type (WT) or mutants 3’UTR binding sites was transfected into Huh-7 cells, along with miR-7 or miR-NC control (Fig. 5D). We found a significant downregulation of reporter activity of the CAV1 3’UTR by miR-7, which was rescued by mutations in the miR-7 binding sites, validating CAV1 as a direct target of miR-7 (Fig. 5D). Additionally, miR-7 overexpression also reduced CAV1 protein expression in Huh-7/SR1 cells (Fig. 5E).

Previously, we reported that miR-7 had anti-cancerous effects in sorafenib-resistant HCC cells [14]. Similarly, in Huh-7/LR cells miR-7 inhibited proliferation in both 2D and 3D cultures (Fig. 5F), partly through reduction in ki67 staining (Fig. 5G), and a decrease in the proportion of cells in the S phase (Fig. 5H). Additionally, miR-7 induced G1 arrest and senescence, as evidenced by increased senescence-associated β-galactosidase (SA-β-Gal) activity and p16 nuclear foci (Fig. 5I, J). Moreover, miR-7 induced apoptosis in Huh-7/LR cells, likely via its regulation of CAV1, as shown by Annexin V-FITC apoptosis assay and elevated c-CASP3 levels (Fig. 5K, L). Similar effects were observed in Huh-7/SR1 cells (Suppl. Fig. S11D–G). The growth inhibitory effects mediated by CAV1 downregulation were also observed in inherently TKI-resistant SNU449 and SNU475 cells (Suppl. Fig. S11H, I). Consistent with the results from siCAV1/shCAV1 experiments (Fig. 2, Suppl. Fig. S3-S4), miR-7 overexpression significantly reduced invasiveness of Huh-7/LR cells by targeting both CAV1 and F-actin (Fig. 5M, N).

Given miR-7’s potential as a CAV1-targeting therapy, we evaluated its delivery efficiency in TKI-resistant cells. We used the commercially available Cy3-labelled miRNA negative control mimic (Red) and unlabelled miR-7 mimic as controls to track cellular uptake and transfection efficiency. Reverse transfection of Huh-7/SR1 cells with Cy3-miR-NC demonstrated rapid intracellular delivery of miR-NC, detectable within 4 h and increasing significantly by 24 h (>90% uptake, Suppl. Fig. S12A).

Given that EGFR is a well-known miR-7 target, western blot analyses confirmed that despite using minimal stabilisation chemistry, miR-7 (Ambion) exerted sustained suppression of EGFR protein levels lasting up to 7 days (Suppl. Fig. S12B) suggesting it’s potential for therapeutic application.

Collectively, these data demonstrate that miR-7 acts as a direct inhibitor of CAV1 and other key genes, with the cumulative effect of these changes contributing to the observed phenotypes, including reduced proliferation, increased senescence, apoptosis and decreased metastasis. Thus, miR-7 ‘replacement therapy’ holds a promise as a potential strategy to reverse pan-TKI resistance in HCC patients by simultaneously targeting CAV1 and other oncogenic drivers.

### miR-7 reprograms selective pathways to overcome lenvatinib and sorafenib resistance via targeted inhibition of FGFR4/P70S6K and AXL-mediated signalling

**Fig. 6:**
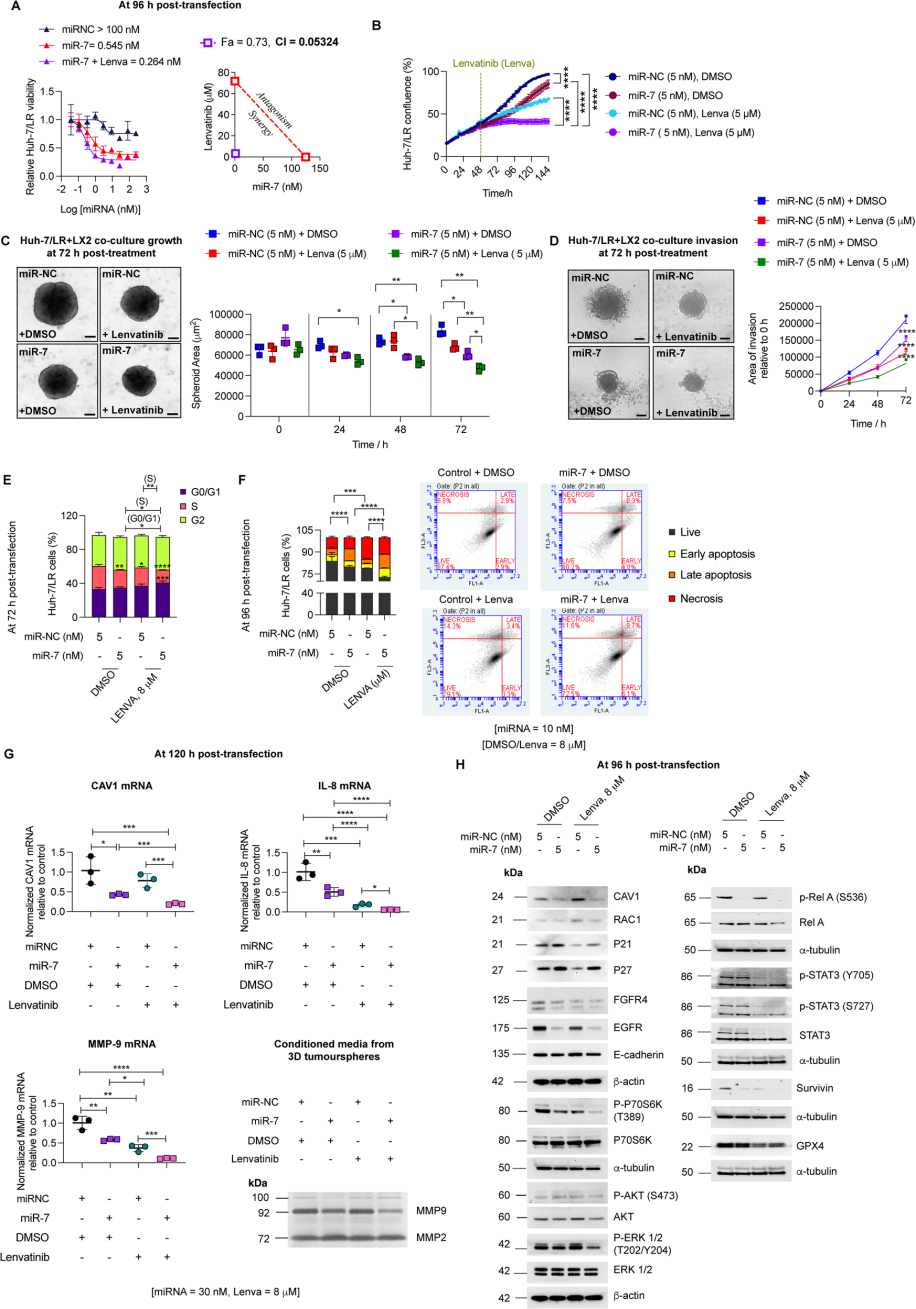
miR-7 and lenvatinib synergistically suppress FGFR4/P70S6K/ERK and NFκB-mediated survival signalling downstream of CAV1 in Huh-7/LR cells. **A** Dose-response effects of miR-7 alone and in combination with lenvatinib, with synergy assessed via CI and isobologram analysis, F_a_ = 0.73 and CI = 0.05324 (*n* = 3). **B** Validation of synergy using IncuCyte growth curve assays (*n* = 3). Effect of low dose miR-7 and lenvatinib on **C** growth and **D** invasion of 3D co-cultured tumour spheroids of Huh-7/LR, LX2 and Matrigel (*n* = 3). **E** Cell cycle analysis of Huh-7/LR cells under different treatment conditions by FACS (*n* = 3). **F** Annexin V-FITC assay to measure apoptosis in Huh-7/LR cells under different treatment conditions (*n* = 3). **G** RT-qPCR for *CAV1*, *IL-8* and *MMP-9* mRNA levels and gelatin zymography of conditioned media from 3D co-culture tumourspheres under different combination treatments (*n* = 3). **H** Western blot analysis for miR-7 targets and evaluation of downstream signalling pathways in Huh-7/LR cells treated with miR-7 and lenvatinib (*n* = 3). All experiments were performed on three independent days with at least three technical replicates. Error bars represent ± SD. Growth curves and time course studies were analysed by one-way repetitive measure ANOVA., while all other data were evaluated by one-way ANOVA with multiple comparisons ( > 2 groups). Significance is denoted as follows: *p < 0.05, **p < 0.01, ***p < 0.001, ****p < 0.0001. Fa= Fractional inhibition, Lenva = Lenvatinib.

We investigated the potential of combining miR-7 with lenvatinib to overcome TKI resistance. miR-7 synergised with lenvatinib, restoring its cytotoxicity in Huh-7/LR cells (Fig. 6A, B, Suppl. Fig. S13A). Additionally, miR-7, both alone and in combination with lenvatinib, exhibited significant anti-proliferative and anti-invasive effects in 3D tumour spheroids generated by co-culturing Huh-7/LR cells with hepatic stroma-derived LX2 cells and Matrigel (Fig. 6C, D, Suppl. Fig. S13B). These effects were mediated primarily through cell cycle arrest at the G0/G1 and S phases, and partly, via induction of apoptosis (Fig. 6E, F).

Pathway analysis of 3D tumour spheroids revealed miR-7, alone and in combination with lenvatinib, significantly reduced CAV1, IL8 and MMP-9 mRNA expression. This treatment also suppressed the inflammatory phenotype of LX2 cells, as demonstrated by decreased levels of *α*-SMA, collagen 1 (COL1A1) and periostin (POSTN) mRNA (Fig. 6G, Suppl. Fig. S13C). The combination also inhibited MMP-9 activity, as evidenced by gelatin zymography analysis of conditioned media from the 3D co-cultures (Fig. 6G). Treating Huh-7/LR cells with miR-7 and lenvatinib significantly inhibited the ERK and NFκB pathway, and induced p21 and p27 expression (Fig. 6H). Similar to siCAV1, miR-7 reduced CAV1, FGFR4 and E-cadherin expression, inhibiting downstream signalling via the P70S6 kinase pathway to overcome TKI resistance (Fig. 6H, Fig. 4).

Furthermore, miR-7 restored sorafenib sensitivity in Huh-7/SR1 cells and the combination was synergistic (Suppl. Fig. S13D). Similar to its action in Huh-7/LR cells, miR-7 mitigated the pro-tumorigenic capacity of Huh-7/SR1 cells predominately via arresting cell division at the G0/G1 and S phase of the cell cycle (Suppl. Fig. S13E) and inducing p21 and p27, while downregulating AXL (Suppl. Fig. S13F). In Huh-7/SR1 cells, miR-7 combined with sorafenib not only inhibited ERK and NFκB pathways but also suppressed survival signalling mediated via the AKT pathway, potentially, downstream of CAV1 and EGFR (Suppl. Fig. S13H).

miR-7 significantly increased glycosylated LAMP1 and LC3 II/I ratios in both Huh-7/SR1 and Huh-7/LR cells, indicating enhanced lysosome-phagosome fusion and autophagy progression (Suppl. Fig. S14A left panel, S15A). Western blot analysis showed P62 levels were significantly reduced in Huh-7/SR1 but remained comparable to miR-NC in Huh-7/LR cells, increasing upon CQ treatment, consistent with induced autophagy (Suppl. Fig. S14A left panel, S15A). These autophagy-promoting effects aligned with elevated SA-*β*-gal activity in miR-7 treated resistant cells (Fig. 5I, J, Suppl. Fig. S11E, F). Notably, miR-7 sustained autophagic flux even under sorafenib exposure in Huh-7/SR1 cells (Suppl. Fig. S14A, middle panel). While miR-7 alone variably affected phospho-AMPKα signalling, its combination with sorafenib consistently attenuated this pathway (Suppl. Fig. S14A, right panel), mirroring the effects of siCAV1 and siAXL (Suppl. Fig. S10). Lysotracker and DQ red BSA assays confirmed miR-7 treatment increased lysosome numbers and enhanced lysosomal enzymatic activity in both Huh-7/SR1 (Suppl. Fig. S14B, C) and Huh-7/LR (Suppl. Fig. S15B, C) cells.

Collectively, our data demonstrate that miR-7 reprograms selective pathways downstream to CAV1 in acquired TKI-resistant cells, specifically targeting the FGFR4/P70S6K /ERK/NFκB pathway in LR cells and the AXL/AKT/ERK/NFκB pathway in sorafenib-resistant cells leading to significant inhibition of cell viability, induction of autophagy and reversal of TKI resistance.

### Clinical implications of CAV1 in HCC

**Fig. 7:**
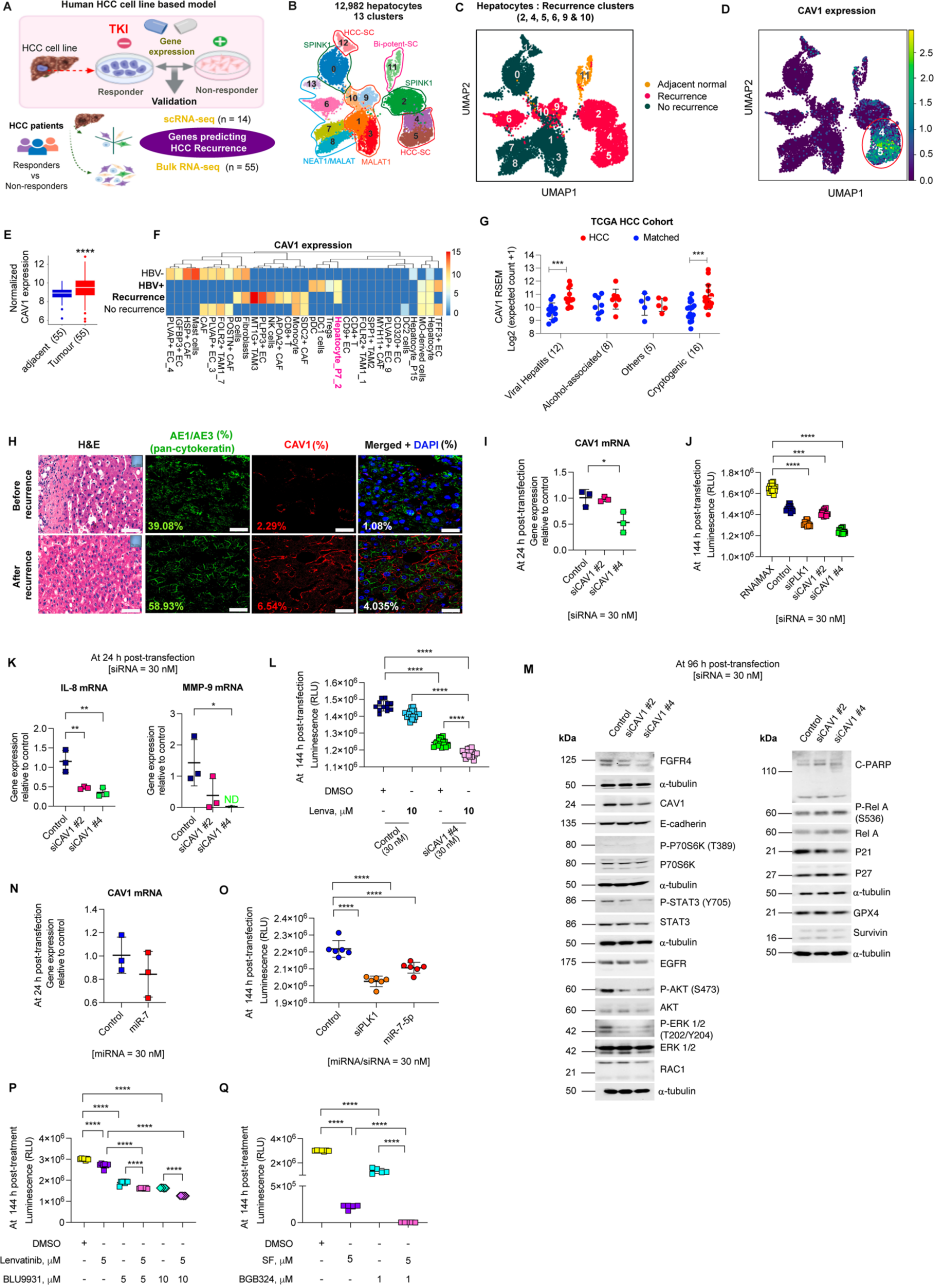
Therapeutic targeting of the CAV1 pathway in HCC. **A** Schematic of CAV1 validation in independent HCC cohorts. **B** Spatial distribution of hepatocyte clusters in HCC. **C** UMAP visualisation of hepatocytes, coloured by prognosis (recurrence and no recurrence). **D** CAV1 expression in recurrence-associated hepatocyte clusters. **E** Bulk RNA-seq analysis of CAV1 expression in HCC patients (*n* = 55). **F** Heatmap of CAV1 expression in HCC cell types, clustered by HBV status and recurrence. **G** CAV1 expression profile in HCC tissues with different etiologies from the TCGA HCC cohort (tumour versus matched normal, *n* = 50). **H** Representative histology sections showing CAV1 expression pre- and post-recurrence, with AE1/AE3 (green) as a pan-cytokeratin marker and CAV1 (red) by double-immunofluorescence immunocytochemistry. **I** RT-qPCR validation of CAV1 knockdown in HCC PDOs using siRNAs. **J** 3D cell viability assay showing the effect of CAV1 knockdown on HCC PDO growth, with siPLK1 to monitor transfection efficiency. **K** RT-qPCR of *IL-8* and *MMP-9* mRNA in CAV1 knockdown PDOs. **L** 3D cell viability assay assessing lenvatinib sensitivity after CAV1 knockdown in HCC PDOs. **M** Western blot of signalling pathways following CAV1 knockdown in HCC PDOs. **N** RT-qPCR of *CAV1* mRNA in miR-7 transfected PDOs. **O** 3D cell viability assay evaluating the effect of miR-7 on HCC PDO growth. **P** Effect of lenvatinib and BLU9931 combination treatment on HCC PDO growth. **Q** Effect of sorafenib and BGB324 combination treatment on HCC PDO growth. For siRNA experiments, RNAiMax was used as a lipid control, and non-targeting siRNA served as the lead control. All experiments were performed on three independent days with at least three technical replicates. Error bars represent ± SD. Data were analysed by one-way ANOVA with multiple comparisons ( > 2 groups) or unpaired two-tailed student’s t-test. Significance is denoted as follows: *p < 0.05, **p < 0.01, ***p < 0.001, ****p < 0.0001. Lenva = Lenvatinib, SF = Sorafenib.

To evaluate the clinical significance of CAV1 in HCC, we compared single-cell RNA-seq (scRNA-seq) data (*n* = 14) with bulk RNA-sequencing data obtained from a separate HCC cohort (*n* = 55) that included patient follow-up information (Fig. 7A). Among 73,589 sequenced cells, 12,982 hepatocytes (cancer cells) were identified and stratified into 13 clusters based on spatial organisation and recurrence risk (Fig. 7B, C). Hepatocytes located in clusters 2, 4, 5, 6, 9 and 10 were specifically associated with disease recurrence (Fig. 7C). Notably, CAV1 expression was confined to clusters 4 and 5, demonstrating a strong correlation with HCC recurrence (Fig. 7D). In contrast, CD47 was broadly expressed across clusters, while FLNC showed no association with recurrence (Suppl. Fig. S16A, B).

Further analysis of the bulk RNA-seq data (*n* = 55) showed significant upregulation of CAV1 in tumour tissues compared to matched normal tissues (Fig. 7E). Using cell specific gene signatures from the scRNA-seq data, we detected CAV1 expression within the reactive stromal constituents, particularly, several immune cell types including cytotoxic CD8 + T cells, natural killer cells (NK), plasmacytoid dendritic cells (pDC) and tumour-associated macrophages (TAM), and other cell types such as PLPP3+ endothelial cells (EC), and lipid metabolising APOA2+ and pro-tumorigenic SDC2+ (syndecan 2) cancer-associated fibroblasts (CAF) (Fig. 7F). Consistent with our previous findings, CAV1 was notably overexpressed in a specific hepatocyte subcluster (P7_2) in HBV+ patients who experienced recurrence (Fig. 7F, pink label).

Building on these findings, we investigated the association between CAV1 expression and HCC aetiology in the TCGA HCC cohort. CAV1 was significantly overexpressed in HCC tissues from patients with viral hepatitis or cryptogenic origin (Fig. 7G). These findings were validated in a larger HBV + HCC cohort (GSE121248, Suppl. Fig. S16C) and in non-alcoholic steatohepatitis-associated HCC, NASH-HCC (GSE164760, Suppl. Fig. S16D), confirming the link between CAV1 overexpression and viral aetiology in HCC.

Next, we stratified HCC patients in the TCGA cohort using CAV1 Youden index and viral aetiology, assessing the impact on clinical outcomes (Suppl. Fig. S16E, F, Table S4). High CAV1 levels were independently associated with poor overall survival (hazard ratio = 1.744, *p* = 0.044), with the worst prognosis observed patients with viral hepatitis HCC (HBV: hazard ratio = 2.039, *p* = 0.025, HCV: hazard ratio = 1.768, *p* = 0.039) (Fig. 7F). Among the viral serology groups, HCV+ serology emerged as an independent predictor of the poorest outcome (hazard ratio = 2.916, *p* < 0.001) (Fig. 7F). Finally, consistent with our cell line data, elevated CAV1 expression in human HCC tissues was accompanied by increased expression of RAC1, CDH1, p21, FGFR4, MMP-9 and AXL compared to healthy liver tissues (Suppl. Fig. S17).

Immunofluorescence analysis of sequential tumour biopsies of HCC patients further confirmed increased CAV1 expression in liver cancer cells following disease recurrence (Fig. 7H).

We also explored the impact of CAV1 inhibition in HCC PDO models. Strikingly, in contrast to our HCC cell-based models, siCAV1 #4 was more potent in downregulating CAV1 mRNA and inhibiting growth in HCC PDOs compared to siCAV1 #2 (Fig. 7I, J, M & Suppl. Fig. S18A, B). CAV1 downregulation restored lenvatinib sensitivity in HCC PDOs by suppressing FGFR4-mediated STAT3/AKT/ERK signalling, reducing IL-8 and MMP-9 mRNA, and p21 protein levels, while inducing c-PARP expression, a marker of apoptosis (Fig. 7K–M, Suppl. Fig. S16G, Suppl. Fig. S18C). A subtle decrease in E-cadherin and EGFR expression was also observed. This HCC PDO exhibited very low basal levels of phospho-P70S6K protein.

Further, we investigated the endogenous regulation of CAV1 using miR-7 in HCC PDOs. miR-7 inhibited PDO growth by reducing CAV1 mRNA levels (Fig. 7N, O, Suppl. Fig. S16H, Suppl. Fig. S18D). Western blot analysis revealed miR-7 exerted its inhibitory effect primarily through suppression of EGFR/AKT signalling and induction of p21, and partially via inhibition of the FGFR4 and ERK pathway (Suppl. Fig. S16I).

Further, we tested the efficacy of BGB324 and BLU9931 in overcoming TKI resistance in HCC PDOs. The PDOs were more sensitive to sorafenib and BGB324, and less sensitive to lenvatinib and BLU9931 (Suppl. Fig. S16J). Notably, the combination of lenvatinib with BLU9931, and sorafenib with BGB324, demonstrated strong synergy in HCC PDOs (Fig. 7P, Q, Suppl. Fig. S18E, F).

These observations strongly support the pivotal role of CAV1 in HCC progression and recurrence. The upregulation of CAV1 in tumour tissues and its association with specific stromal constituents underscore its potential as a therapeutic target. The encouraging results from testing siCAV1, miR-7 and BGB324 and BLU9931 in HCC PDOs independently validated our cell line data, emphasising the importance of stabilising chemistries for siRNA/miRNA-based therapies and revealing potential strategies to overcome TKI resistance in HCC.

**Fig. 8:**
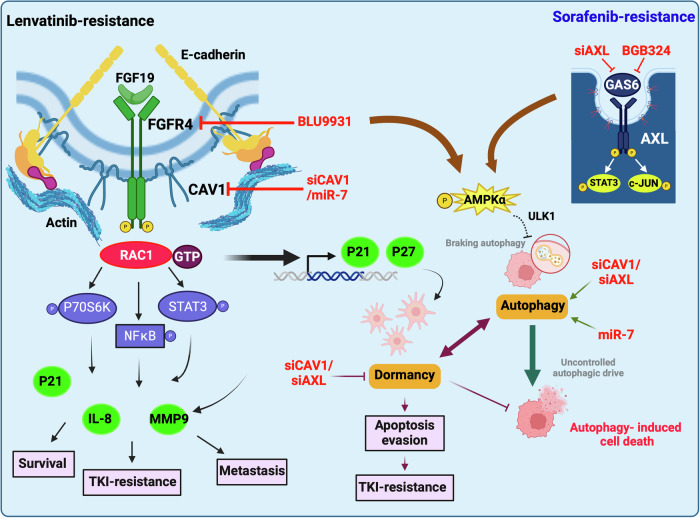
A proposed model for overcoming TKI resistance via targeting the CAV1 and AXL pathways. HCC patients with elevated CAV1 and AXL expression exhibit poor responses to targeted therapies. Both CAV1 and AXL are essential for survival, invasion and chemotaxis of cancer cells. Mechanistically, CAV1 primarily acts via the STAT3/P70S6K/NFkB pathway, while AXL regulates STAT3/c-Jun axis in TKI-resistant cells. CAV1 via its interaction with E-cadherin can modulate FGFR4 expression and activity in lenvatinib-resistant cells, as well as AXL in sorafenib-resistant cells. In addition, CAV1 overexpression enhances RAC1 activity, leading to increased transcription of P21 and concurrent activation of the AMPKα pathway. This, in turn, induces a slower proliferative rate (dormancy), suppresses autophagy, and promotes evasion of apoptosis. AXL can also indirectly influence the AMPKα pathway. Targeting both CAV1 and AXL using selective siRNAs or miR-7 disrupts this protective autophagic brake, triggering excessive, uncontrolled autophagy, which ultimately drives rapid cancer cell death and overcomes resistance to sorafenib and lenvatinib.

As summarised in Fig. 8, we propose a mechanistic model in which dual targeting of CAV1 and AXL impairs the pro-survival signalling via STAT3, AMPKα, and RAC pathways, thereby promoting excessive autophagy and inducing cancer cell death to overcome TKI resistance in HCC.

## Discussion

Aberrant CAV1 expression in HCC is consistently associated with poor prognosis and rapid disease progression [24, 26, 32–36]. Several studies have shown that CAV1 plays a significant role in promoting migration, invasion, metastasis, metabolism and angiogenesis in HCC. For instance, CAV1 inhibits autophagy [28], and drives metabolism via a hexokinase 2-dependent mechanism [37]. However, its role in driving TKI resistance in HCC remains less well understood.

In this study, we demonstrate for the first time that multiple TKI-resistant HCC cell lines, which recapitulate the molecular features of in vivo resistance, share a common mechanism of pan-TKI resistance driven via the CAV1 signalling pathway. This pathway modulates key regulators of cellular dormancy, such as RAC1, E-cadherin, and p21, influencing critical downstream survival pathways including P70S6K, STAT3 and NFκB and autophagy. Importantly, we show that targeting FGFR4 and AXL, both downstream of the CAV1 pathway, overcomes resistance to lenvatinib and sorafenib. Additionally, we identify *CAV1* as a direct target of the tumour suppressor miRNA, miR-7-5p, and this regulatory axis is conserved in clinical HCC samples. Elevated *CAV1* expression correlates strongly with disease recurrence and poor response to sorafenib therapy, particularly in HBV+ HCC patients, where high *CAV1* levels predict worse clinical outcomes. These findings elucidate a new mechanism of pan-TKI resistance mediated by CAV1, offering new avenues for overcoming therapeutic resistance in HCC.

Several mechanisms have been implicated in TKI resistance in HCC, including activation of EGFR, PI3K/AKT, MAPK, and JAK/STAT3 pathways, EMT, hypoxia, cancer cell stemness and metabolic reprogramming [38, 39]. Our study adds to this body of work by showing that *CAV1* expression is negatively correlated with TKI sensitivity across 785 human cancer cell lines, regardless of origin or mutation burden, suggesting a broader oncogenic role of CAV1 in promoting TKI resistance. These observations were validated clinically in sorafenib non-responder HCC patients, further strengthening our findings.

We found that inducing CAV1 expression in both TKI-naïve and TKI-resistant HCC cells altered levels of E-cadherin, p21 and RAC1/P70S6K/STAT3 pathway, promoting survival, invasiveness and chemoresistance. Previous studies demonstrated that CAV1 regulates E-cadherin expression by inhibiting *β*-catenin-TCF/LEF1-dependent transcription of Snail [40] and exploits E-cadherin to inhibit colorectal cancer cell growth by sequestering β-catenin at the plasma membrane, which prevents transcription of survival genes such as survivin [41]. Additionally, E-cadherin anchors to the F-actin cytoskeleton via its interaction with *β*- and *α*-catenin, maintaining cell-cell contact and modulating signalling via RAC1 kinases [42].

In stark contrast, our data show that in TKI-resistant cells, CAV1 functions as an oncogene by stimulating E-cadherin expression independently of inhibition of the *β*-catenin pathway and activated signalling via RAC1/P70S6K and p21 axes. Consistent with this, recent research highlighted the role of E-cadherin in promoting the collective dissemination and survival of breast cancer cells at distant sites [43] as well as its contribution to chemotherapy resistance in ovarian and colon cancer cells [44–46]. In addition, RAC1 has been implicated in inducing tumour dormancy and conferring resistance to chemotherapies in environments characterised by a stiff matrix. This is achieved through the nuclear translocation of RAC1 and the subsequent transcriptional activation of the epigenetic modifier TET2 leading to increased levels of p21 and p27 [47], which significantly influence tumour behaviour.

In our experimental models, Huh-7 and PLC-PRF-5 cells were used, both of which harbour p53 mutations (Y220C and R249S, respectively) that lead to their reduced DNA binding capacity and impaired p21 activation [48, 49]. This means in the absence of functional p53, CAV1 may augment the oncogenic activity of p21, thereby extending its role beyond merely regulating tumour dormancy. Consistent with this notion, recent studies indicate that in p53-deficient cells, p21 suppresses apoptosis by inhibiting caspase cleavage by TRAIL death receptor DR4, Fas- and prostaglandin A2-mediated pathways [50]. The dual role of p21 in cancer may also depend on its subcellular localisation, with elevated cytoplasmic levels favouring tumour growth and survival. This complex interplay underscores the sophisticated mechanisms cancer cells employ to resist therapeutic strategies, presenting potential targets for novel treatments.

Exploring the survival mechanism in TKI-resistant cells, we observed a marked decelerated growth rate, attributable to EMT and the establishment of dormancy. These cells relied on autophagy to sustain their metabolic requirements and survival. We found that both sorafenib and lenvatinib activated the AMPKα pathway in our TKI-resistant cells, reflecting their low energy states. Intriguingly, CAV1 overexpression also activated the AMPKα pathway, suggesting that CAV1 upregulation could be a regulatory factor enhancing AMPKα activity in resistant cells. Known for its critical role in cellular metabolism, CAV1 in the context of pan-TKI resistance appears to negatively regulate and sustain basal autophagy via its effect on AMPKα signalling. Supporting this, new evidence suggests that under adverse conditions, activation of AMPKα suppresses initiation of autophagy by inhibiting ULK1, while concurrently safeguarding other autophagy regulators from degradation, thereby moderating autophagy levels and promoting cell survival [51].

Our findings reveal, CAV1 knockdown led to a reduction in expression of E-cadherin and p21, increased ki67 positivity, and enhanced cancer stemness markers, alongside diminished AMPKα signalling. We think this modulation provoked uncontrolled autophagy, inhibiting critical survival signalling pathways and initiating apoptosis, with partial induction of ferroptosis, as evidenced by reduced GPX4 levels. These molecular alterations appear to reactivate dormant tumour cells and restore TKI sensitivity in resistant cells. These results emphasise the crucial role of CAV1 in mediating TKI resistance, primarily through its regulation of autophagy and tumour dormancy, coordinated via the E-cadherin and RAC1/P70S6K /p21 axes.

AXL expression in HCC has been strongly associated with advanced disease stage [52], poor prognosis [52] and resistance to sorafenib [53, 54]. Several oncogenic pathways, including YAP/ERK [1] and TGFβ/AKT(52), had been shown to promote tumorigenic and metastatic behaviours in HCC via AXL signalling. Our group, along with others, has demonstrated that AXL contributes to resistance against targeted therapies across various solid tumours, including HCC [53, 55, 56]. Moreover, elevated levels of soluble AXL have been detected in the serum of sorafenib non-responders [53] and cirrhotic HCC cases with progressive disease [57]. Consistent with previous studies [53, 54], we observed a significant upregulation of AXL expression in both acquired and inherently sorafenib-resistant HCC cells. AXL promotes survival, chemotaxis, and the invasive potential of these cells, in part by modulating autophagy, thereby facilitating resistance to sorafenib. Notably, we identified that CAV1, via its interaction with E-cadherin, stabilises AXL at the surface of Huh-7/SR1 cells. Targeting AXL with selective siRNAs or silencing CAV1 (siCAV1) led to diminished AXL expression, accelerated autophagic cell death,h and restored sensitivity to sorafenib. Importantly, we found that HCC cells exhibit a high degree of sensitivity to the selective AXL inhibitor BGB324, independent of AXL expression levels, and combining siCAV1 or BGB324 with sorafenib resulted in a significant reduction in the growth of HCC cells and PDOs.

Recent work by Xie et al. demonstrated that elevated AXL expression in TKI-resistant murine HCC models induces an immunosuppressive microenvironment, reducing the efficacy of anti-PD1 therapy, and was reversed by combining BGB324 with sorafenib and anti-PD1 therapy [54]. Further, high AXL expression in HCC patients could predict poor response to immunotherapy [54]. Similarly, early data from a phase I/II trial investigating bemcentinib (BGB324) with the PD-1 blocker pembrolizumab in resistant non-small cell lung cancer showed a 24% response rate, rising to 40% in AXL positive patients, with manageable toxicity, underscoring both the potential for efficacy in general and the value of AXL expression as a predictive marker [58].

Beyond its role in HCC, AXL signalling has been implicated in promoting short-term dormancy in prostate cancer cells, thereby prolonging tumour latency before disease recurrence [59]. In multiple myeloma, AXL enhances both chemotherapy resistance and dormancy in drug-resistant cells [60]. In prostate cancer, AXL operates in conjunction with other dormancy-associated factors to maintain cellular quiescence [59]. Our findings indicate that AXL depletion facilitates the transition of resistant cells from GO/G1 phase to the G2/M phase of the cell cycle, as evidenced by an increase in the proliferation marker ki67. However, similar to the effects of siCAV1, these cells fail to progress into mitosis, coinciding with a decline in cyclin D1 levels and induction of autophagy-mediated apoptosis. These findings suggest that AXL and CAV1 may collaborate to sustain HCC dormancy, potentially contributing to tumour relapse and underscoring their multifaceted roles during HCC progression and therapeutic resistance.

FGFR4 is the predominant FGFR isoform in hepatocytes, and the FGF19/FGFR4 axis promotes EMT through GSK3β phosphorylation and activation of the β-catenin/TCF4 pathway in HCC [61]. Emerging evidence indicates that high FGFR4 expression and Treg infiltration contribute to an immunosuppressive microenvironment. Yi et al. [62] demonstrated in murine HCC models that lenvatinib blocks FGFR4/GSK3β signalling, suppresses tumour growth, and promotes ubiquitination and proteasomal degradation of PDL-1. Lenvatinib also inhibits STAT5 signalling, preventing CD4 + T cell differentiation into Tregs, and synergises with pembrolizumab, an anti-PD1 therapy [62]. However, despite promising in phase II LEAP-002 trials, this combination showed reduced efficacy in phase III studies [3].

Our data reveal that during the acquisition of lenvatinib resistance, Huh-7/LR cells lose their response to lenvatinib-mediated FGFR4 suppression, in contrast to the parental Huh-7 cells, which remain sensitive. Notably, CAV1 overexpression in parental Huh-7 cells reinforced FGFR4 expression, conferring lenvatinib resistance, whereas CAV1 depletion in Huh-7/LR cells reduced FGFR4 levels, restoring sensitivity to the drug. Both CAV1 and E-cadherin are known regulators of RTK activity at the plasma membrane. Our results suggest that in Huh-7/LR cells, CAV1 knockdown, destabilises the plasma membrane, leading to loss of E-cadherin and FGFR4, thereby disrupting downstream signalling cascades critical for survival, ultimately inducing cell death. Furthermore, selective FGFR4 inhibition with BLU9931 synergised with lenvatinib, enhancing its anti-tumour efficacy in both HCC cell lines and PDOs. These findings imply that the reduced efficacy of lenvatinib and pembrolizumab in the phase III LEAP-002 study may be attributed to lenvatinib resistance caused by impaired FGFR blockade. We propose that combining BLU9931 or siCAV1 with lenvatinib and pembrolizumab may offer more durable clinical benefits for HCC patients.

Our clinical analysis using scRNA RNA-seq and bulk RNA-seq data showed that CAV1+ cancer cells in HCC tissues are associated with higher recurrence rates. CAV1 expression was also enriched within the stroma of recurrent disease implying CAV1’s role in establishing a tumour-permissive microenvironment. Indeed, HCC recurrence often occurs in regions of stiff liver due to underlying cirrhosis [63]. Our study found under drug treatment, CAV1 expression is elevated in TKI-resistant cells, which remodels their plasma membrane, increases cellular stiffness, and alters responses to survival signals. Conversely, CAV1 expression can also be regulated by matrix stiffness [64] as caveolae play a crucial role in mechano-adaptation by rapidly adjusting plasma membrane tension in response to mechanical stimuli. Thus, the elevated CAV1 expression we observed in stromal cells within areas of recurrence may arise as an adaptive response to the stiff microenvironment or harsh conditions such as hypoxia, further activating cellular crosstalk via mechano-signalling pathways [65]. Accumulating evidence suggests that CAFs and TAMs secrete cytokines like IL-8 and TGFβ1, and extracellular matrix (ECM) remodelling enzymes, generating an immunosuppressive microenvironment. This milieu traps CD8 + T cells and NK cells, rendering them dysfunctional and prompting tumour progression [66]. For instance, in gastric cancer, SDC2 + CAFs exhibit increased cross-talk with neighbouring CD4 + /CD8 + T cells and NK cells, establishing immunosuppressive signalling pathways, and stromal SDC2 correlates with advanced stage and poor prognosis [67]. In fact, in cirrhosis, mechanical cues from the rigid matrix increase CAV1 activity in CAFs, promote caveolae formation, and facilitate secretion of tissue inhibitors of metalloproteinases (TIMP) to remodel the ECM [65]. *PLPP3*, which encodes lipid phosphate phosphatase, helps maintain vascular barrier integrity during inflammation by dephosphorylating membrane lipids of ECs [68]. While T-lymphocytes express low CAV1, its redistribution in polarised membrane rafts is necessary for coupling between T-cell receptors and co-stimulators upon encountering antigen-presenting dendritic cells [69]. The antigen engagement triggers F-actin polymerisation to form immune synapses, enhancing cytotoxicity and selectively transducing NFAT-mediated signals (e.g., IFNγ, IL-2 and TNF-*α*) over NFκB. T-lymphocytes deficient in CAV1 are defective, highlighting its essential role in CD8 + T cell immunity [69]. Additionally, pDC cells are known to promote the differentiation of CD4 + T cells to Tregs [66]. Our data suggest that downregulating CAV1 in HCC PDOs targets IL-8 and MMP-9, both critical players in immune evasion. Given CAV1’s role in both tumour cells and stroma, therapeutic targeting of CAV1 in HCC may have dual benefits: inhibiting cancer cells while modulating the pro-carcinogenic microenvironment, potentially sensitising tumour cells to immunotherapy.

HBV + HCC has one of the worst prognoses among HCC subtypes. In the Asia Pacific trial, sorafenib was less effective in HBV + HCC patients [1], and the SHARP trial showed rapid radiological progression in sorafenib-treated HBV + HCC cases [1]. Notably, CAV1 expression is significantly increased in archival tissues from HBV + HCC patients [25]. HBx, an HBV protein, is a major driver of EMT and HCC metastasis in HBV + HCC, and its mutant form was recently shown to stimulate CAV1 transcription and function in HCC [33, 70]. Our findings confirm this elevation of CAV1 in HBV + HCC, along with its strong association with unfavourable clinical outcomes. Furthermore, we observed that CAV1 expression increases in patients progressing from NASH to NASH-HCC; however, high expression was also noted in non-tumour peripheral tissues. Importantly, our observation of APOA2+ CAFs in recurrent HCC patients demonstrated a gene signature linked to enhanced lipid metabolism. Consistent with our observations, a recent study showed that CAV1 promotes cancer cell survival in the fatty-acid-rich microenvironment of NAFLD [71], suggesting CAV1 also aids the adaptation of non-cancerous cells in such environments. This is particularly significant, as NAFLD is predicted to become the leading cause of HCC in the future due to advances in disease surveillance, immunisation and anti-viral therapies reducing virus-related HCC incidence.

Although no commercially available small molecule inhibitors of CAV1 exist, the recent development of RNA-based therapies, such as the FDA-approved siRNA targeting PCSK9, inclisiran, heralds a new therapeutic era [72]. Our study identifies CAV1 as a key determinant of TKI resistance and HCC recurrence. We have discovered novel approaches to downregulate CAV1, potentially overcoming TKI resistance and offering a new therapeutic strategy for HCC patients. This discovery aligns with the latest advances in medicinal therapies, providing hope for more effective treatments in the future.

Supplementary information is available at Cell Death and Disease’s website.

## Supplementary information

Supplementary Materials, Methods and Figures

Supp Table 1

uncropped membranes

Biorender Publication license

## Data Availability

RNA sequencing data are deposited in GEO (GEO accession number: GSE200098).
