# Supplementary material for: Inhibition of the Caveolin-1 pathway promotes apoptosis and overcomes pan-tyrosine kinase inhibitor resistance in hepatocellular carcinoma
Source: Cell Death Dis. 2025 Jul 25;16(1):561. doi: 10.1038/s41419-025-07887-4 (PMC12297225; doi:10.1038/s41419-025-07887-4)
Supplement: Supplementary file 1 — Supplementary Materials, Methods and Figures [file 41419_2025_7887_MOESM1_ESM.pdf]

Inhibition of the Caveolin-1 pathway promotes apoptosis and overcomes pan-tyrosine kinase inhibitor resistance in hepatocellular carcinoma

Tasnuva D. Kabir[1], Samuel Beck[2], Lisa M. Stuart[1], Ji Li[1], Rui Hou[1], Peiwen Liu[3], Shelby Margolis[1], Claire Kim[4], Yu Suk Choi[4], Edward R. Bastow[1], Dianne J. Beveridge[1], Lisa Spalding[5,6], Ziyi Li[7], Florent Ginhoux[7,8,9], Pierce Chow[10], Michael Phillips[1], Andrew D. Redfern[1,5,6], The Liver Cancer Collaborative*, George C. Yeoh[1], Alistair Forrest[1], Andrew J. Woo[1,11], Ankur Sharma[12], Jacob George[13], Geoffrey McCaughan[14] and Peter J. Leedman[1,4].

[1]Harry Perkins Institute of Medical Research, QEII Medical Centre, Nedlands and Centre for Medical Research, The University of Western Australia, Crawley, WA, 6009, Australia. [2]Department of Dermatology, Boston University School of Medicine & Boston Medical Center, Boston, MA, USA. [3]School of Science, Zhejiang Sci-Tech University, Hangzhou, China. [4]School of Human Sciences and [5]Medical School, The University of Western Australia, Crawley, WA, 6009, Australia. [6]Department of Medical Oncology, Fiona Stanley Hospital, Murdoch, WA, 6150, Australia. [7]Shanghai Institute of Immunology, Department of Immunology and Microbiology, Shanghai Jiao Tong University School of Medicine, Shanghai, 200025, China. [8]Singapore Immunology Network (SIgN), Agency for Science, Technology and Research (A*STAR), 138648, Singapore. [9]Translational Immunology Institute, SingHealth Duke-NUS Academic Medical Centre, 169856, Singapore. [10]National Cancer Center Singapore, 11 Medical Drive, Singapore. [11]Centre for Precision Health, Edith Cowan University, Joondalup, WA, 6027, Australia. [12]Translational Genomics Program, Garvan Institute of Medical Research, Darlinghurst, NSW, 2010, Australia. [13]Storr Liver Centre, Westmead Institute for Medical Research, Westmead Hospital and University of Sydney, NSW, Australia, [14]Liver Injury and Cancer, Centenary Institute, Sydney, Australia.

*A list of authors and their affiliations appears at the end of paper.

Corresponding author: Peter J Leedman, Harry Perkins Institute of Medical Research, 6 Verdun Street, Nedlands, 6009, Western Australia, Australia, tel: +61861510704, fax: +61861510701, peter.leedman@perkins.org.au

Supplementary Materials and Methods:

Human archival HCC specimens: Formalin fixed paraffin embedded HCC tissues and corresponding coded patient information were obtained from Royal Perth Hospital (n = 23). The study was approved by Royal Perth Hospital Human Research Ethics Committee: application number 2016-090.

Cell Culture: The human HCC cell lines Huh-7 and Hep3B were kindly provided by Professor Nicholas Shackel, Ingham Institute at Liverpool Hospital, Sydney, Australia. LX2 cell line was provided by Professor Jacob George, Westmead Institute of Medical Research, Sydney, Australia. All other cell lines were from ATCC. Cell culture media and growth supplements were sourced from Perkins Media Lab. Huh-7 and Hep3B cells were cultured in DMEM low glucose with 10% FBS, 4 mM L-glutamine; SNU449 and SNU475 and SNU423 cells were cultured in RPMI (high glucose) and 10% FBS (heat-inactivated); SNU387 cells were cultured in RPMI (high glucose) and 10% FBS; SK-HEP1, PLC-PRF-5 and HepG2 cells were cultured in EMEM and 10% FBS; HEK293T cells were cultured in DMEM high glucose supplemented with 2 mM L-glutamine and 10% FBS. LX2 cells were cultured in DMEM (high glucose) supplemented with 2% FBS. All cells were cultured at 37° C and 5% CO_2. Mycoplasma contamination was excluded via a PCR-based method ensuring that only mycoplasma-free cells were used in subsequent experiments. Cell identifications were confirmed by short tandem repeat profiling.

Generation of sorafenib-resistant and lenvatinib-resistant HCC cells: To generate sorafenib-resistant HCC cells: Huh-7/SR1 (SR1), Huh-7/SR2 (SR2), Hep3B/SR1 and Hep3B/SR2, and lenvatinib-resistant Huh-7/LR cells the HCC cell lines were cultured with increasing concentrations of sorafenib (BAY 43-9006, Selleckchem, #S7397) or lenvatinib (E7080) mesylate, (Selleckchem, #S5240), respectively as described previously (1). The resistant cells were constantly maintained with sorafenib ranging from 7-10 μM and lenvatinib at 8 μM.

Transfection with siRNA and miRNAs: Synthetic silencer human CAV1 siRNA #1 (siCAV1 #1, ID#10297), #2 (siCAV1 #2, ID#10479), #3 (siCAV1 #3, ID#145951) and #4 (siCAV1 #3, ID#145953) (Cat#AM16708), silencer select human AXL siRNA #45 (siAXL #45, ID# s1845) and #47 (siAXL #47, ID# s1847), Ambion silencer negative control #1 siRNA (siNC, cat#AM4611), miRNA precursor hsa-miR-7-5p (miR-7, ID#PM10047, Cat# AM17100) and miRNA mimic negative control #1 (miR-NC, ID#4464058) were sourced from Thermofisher Scientific and resuspended in nuclease free water at 50 μM stock, aliquoted and stored at -

20° C. HCC cell lines were seeded at a density of 5 X 10^5 per well in a 6-well plate. Both miRNAs and siRNAs were transfected into the cells using reverse transfection method using lipofectamine 2000 (Invitrogen, #11668019) reagent in OptiMEM I reduced serum medium (Gibco, #31985070) to a final concentration of 30, 10 and 5 nM, respectively, according to manufacturer's instruction. For validation of siRNA-mediated knockdown efficacy or miRNA overexpression with synthetic mimics, total RNA was extracted at 24 h post-transfection. For pathway analysis at gene expression level, RNA extraction was performed at 72 h post-transfection using 30 nM siRNAs. All protein analyses were carried out at 96 h and 168 h post-transfection using siRNAs or miRNAs.

Luciferase reporter assay: The pmiR-REPORT-CAV1 3'UTR wild type (henceforth WT) and pmiR-REPORT-CAV1 3'UTR mutants (henceforth Mut) were synthesized by GenScript Inc. (Piscataway, NJ) to include the full length WT 3'UTR sequence of human CAV1 (Ensembl ID: ENST00000341049.7, nt 565 – 2456, refseq accession number NM001753.5) and predicted miR-7 target sites modified at nt 1505 -1511 and 1512-1518 of CAV1 3"UTR (TargetScan Human 7.1) to 'AGAAGG' with sequences confirmed prior use. The perfect miR-7 target construct was also generated by GenScript. Briefly, Huh-7 cells were reverse transfected with 250 ng of either pmiR-REPORT-CAV1 3'UTR WT or mutant constructs in combination with either miR-NC or miR-7 (30 nM) using lipofectamine 3000 and P3000 reagent ((Invitrogen, #L3000015) in OptiMEM I reduced serum medium following manufacturer's instructions. Luciferase activity was measured 24 h after transfection using the Dual Luciferase Reporter Assay System (Promega, #E1910) according to manufacturer's protocol. Firefly luciferase activity was normalised to Renilla luciferase, and the ratio of each sample was further normalized to miR-NC-WT control.

To assess the effects of miR-7 and siCAV1 #2 on STAT3 and NFκB signalling in Huh-7/SR1 cells, cells were transiently co-transfected with either miR-7/miR-NC (5 nM) or siCAV1 #2/siNC (5 nM) and either Cignal STAT3 reporter (luc) vector (100 ng, Qiagen, CCS-9028L)) or a Cignal NFκB reporter (luc) vector (100 ng, Qiagen,CCS-013L) using lipofectamine 2000. Luciferase activity was measured 48 h post-transfection following manufacturer's protocol and was normalized to protein concentration. Statistical significance was determined using unpaired student's t-test.

Transformation and plasmid extraction: Chemocompetent *E. coli* were transformed using heat shock and seeded on ampicillin plates. Single clones were picked the next day and propagated in selective LB broth. Plasmids were harvested according to manufacturer's instructions (Qiagen plasmid plus midi kit).

Generation of stable over-expression and knockdown human HCC cell lines: To stably overexpress CAV1 V5 tagged 3rd generation lentiviral vector PLX304-Blast containing an empty backbone (Empty-V5-PLX304-Blast, #25890) and human CAV1 ORF (CAV1-V5-PLX304-Blast, ENTREZGENE ID 857, Insert ID: BC009685.1, insert size: 956 bp) were purchased from Dharmacon (Millennium Science Pty Ltd., Australia). For stable knockdown of CAV1, pre-designed shRNA clones (TRCN0000007999; shCAV1 #7999, TRCN0000008000; shCAV1 #8000, and TRCN0000008002; shCAV1 #8002) in the pLKO.1 backbone were purchased from Sigma Aldrich. A non-targeting shRNA control served as negative control. Lentiviral particles were produced by co-transfecting HEK293T cells with desired plasmids (2 μg) and the packaging elements [p123 gagpol (2.6 μg), p124 gagpol (0.95 μg) and p125 VSVG (4 μg)] using Fugene HD (Promega, E2311) in optiMEM I reduced serum media. Viral particles were harvested 48 h post-transfection. HCC cells were seeded at a density of 4 X 10^5/well of a 6-well plate followed by infection with a single dose of the lentivirus supernatant in polybrene (1:1000) for 48 h, and subsequent selection with blastocidin (10 μM 48 hourly, InvivoGen, #ant-bl-05) for 2 weeks or puromycin (10 μM; SNU449 and 2.5 μM; SNU475, 72 hourly, Sigma). Gene overexpression and knockdown were confirmed by quantitative RT-qPCR and western blot.

RNA extraction: Total RNA extraction was extracted using the mirVana miRNA isolation kit (Ambion, #AM1560) for miRNA expression studies and the RNeasy Mini Kit (Qiagen, #74104) for all other gene expression analyses, as per manufacturer's instructions. RNA integrity was routinely assessed using a Nanodrop One spectrophotometer (Thermo Fisher Scientific). For RNA-sequencing, total RNA was extracted from HCC cell lines using the mirVana miRNA isolation kit, and purity was verified using the 2100 Bioanalyzer instrument (Agilent) at the Australian Genome Research Facility (AGRF, Perth, Western Australia). Only samples with an RNA Integrity number (RIN) 10/10 were used for library preparation.

RNA sequencing: Total RNA (1 μg) from Huh-7/SR1 and Huh-7/SR2 cells (in triplicate) was sent to AGRF for library preparation and strand specific RNA-sequencing (RNA-seq) using the Illumina HiSeq 2500 system with an HT chemistry platform, yielding 100 million 50 bp single-end reads. Raw RNA-seq data underwent quality control and were aligned to the GRCh37 human genome using StringTie tool. Transcript abundance was estimated in *R* using EdgeR package, and differentially expressed genes were identified by K-means clustering using med-centroid $\log_2$ fold change approach. Gene expression changes with a FDR<0.01 were considered statistically significant (Suppl. table S1 and S5).

Single cell RNA-sequencing: HCC patient description and a detail of single cell RNA-sequencing method is described elsewhere(2).

Deconvolution of bulk RNA-seq data: Bulk RNA-sequencing data was deconvoluted using CIBERSORTx method and NanoString digital spatial profiling(3). First, a gene signature matrix was generated from single cell RNA-seq data, merging cells with similar expression profiles into specific clusters. The gene signature matrix was used to estimate cell type composition for each sample (parameters: "– single_cellTRUE-fraction 0.2"). To determine accuracy of CIBERSORTx method, one third of single cells were used to train algorithm and the remaining two third cells were used for testing. Testing cells were split into three replicates to create a pseudo-bulk mixture. Pearson's correlations were calculated between CIBERSORTx-inferred proportions and actual cluster abundances. Additionally, MuSiC (v0.2.0)(4) was used for deconvolution, and cosine similarity between MuSiC- and CIBERSORTx-inferred proportions was using Lsa package.

Pathway analysis: Gene set enrichment analysis (GSEA) was performed using GSEA software (Broad Institute, v4.1.0) with the C5 (GO gene sets) and H (hallmark gene sets) collections from MSigDB, v7.0. Only significantly enriched categories were considered, excluding those fewer than 15 genes, nom-p-value ≤ 0.05, and a FDR q-value ≤ 0.25. GSEA was conducted with Signal2noise metric, 1000 permutations, permutation type set to gene set, and weighted enrichment statistics.

Retrieval of gene expression data from public databases and bioinformatics analysis: To compare the differentially expressed gene signature of Huh-7/SR1 and Huh-7/SR2 cells with previously reported datasets, we downloaded Affymetrix human transcriptome array (v2.0) data of Huh-7 and its sorafenib-resistant clone A7 (GSE94550), Affymetrix human gene (v1.0) ST array data of HepG2 and its sorafenib-resistant subline HepG2S1 (GSE62813), RNA-seq data of HepG2 and its sorafenib-resistant derivative (GSE128683), and Huh-7 and its lenvatinib-resistant derivative (GSE211850) from ncbi.nlm.nih.gov/GEO database. Normalized expression data were analysed using the integrated iDEP.90 platform(5). Differentially expressed genes were identified by DESeq2 using a 2-fold change cut-off point and FDR<0.01. Overlapping genes were identified with VENNY 2.1, and statistical significance was determined by hypergeometric test calculator from the Graeber lab (copyright 2009).

To validate *CAV1* expression in HCC cases, normalized gene expression data were downloaded from the GDC portal for the TCGA LIHC cohort and from GEO database (GSE164760 for NASH-HCC, GSE121248 for HBV-HCC and GSE109211 for BIOSTORM

HCC cohort). Clinical data from the TCGA cohort were analysed using Cox Regression by Breslow method of Ties to evaluate *CAV1* expression and clinical outcomes. Receiver operating characteristic (ROC) curves were generated in GraphPad Prism to assess the predictive value of *CAV1* for HCC outcome, using *CAV1* expression profile in GTEX healthy liver tissue and TCGA HCC samples. Youden index (J) was calculated using the formula J = sensitivity+specificity-1. A Chi^2 test was used to determine statistical significance. Additionally, *CAV1* expression in various HCC cohorts of different etiology was compared by unpaired student's t-test. Furthermore, ROC curves were generated for *CAV1* and *CD47* using gene expression data from sorafenib responders and non-responders to evaluate their predictive value in sorafenib resistance.

Correlation of genes with TKI sensitivity: To assess the relationship between basal gene expression and TKI sensitivity, normalized mRNA expression data (log_2 scale) of 791 human cancer cell lines, including 22 human HCC cell lines, were extracted from the Cancer Cell Line Encyclopaedia (CCLE; Broad Institute). Corresponding area under the curve (AUC) for sorafenib, lenvatinib, cabozantinib and regorafenib, were sourced from the Cancer Therapeutic Response Portal 2.0 (CTRP v2.0; Broad Institute). Drug target expression and sensitivity values were extracted for each cell lines, with sensitivity defined as [1-(AUC/30)], following established methods(6). Spearman correlations were calculated between gene expression and drug sensitivity across 823 cell lines, with significance determined by randomizing expression data and reanalysing 1,000, times. Drug-target pairs were considered significant when observed correlation coefficients exceeded those from randomized datasets(6).

Gene expression assays: RNA (500-750 ng) was reverse transcribed into cDNA using a High-Capacity cDNA Reverse transcription kit (Applied Biosystem, #4368814), according to manufacturer's instructions. Real-time quantitative PCR was performed on the ViiA7 Real-time PCR system (Life Technologies) using TaqMan Fast Advanced Master Mix (Applied Biosystem, #4444557) and PowerUp SYBR Green Master Mix (Applied Biosystem, #A25742), according to manufacturer's protocol. Primer efficiency was checked by melt curve analysis. Gene expression was normalized to *GAPDH* (Thermofisher Scientific, #4333764F) or *RNU6B* (Thermofisher Scientific, #4427975), and relative expression of the target genes was calculated using the $2^{-\Delta\Delta CT}$ method. Reagents and primer sequences are detailed in the table S6.

Protein lysate preparation and western blot: Whole cell lysates were prepared by 1X cell lysis buffer (Cell Signaling, #9803) supplemented with 1 mM PMSF (Sigma, #10837091001),

1X cOmplete EDTA-free protease inhibitor cocktail (Roche, #11873580001) and 1X PhosSTOP (Roche, #4906845001). Total protein was quantified using Bradford assay (BioRad, #500-0006). Lysates (20 -50 µg) were resolved on NuPAGE 4-12% Bis-Tris protein gels (Invitrogen) and transferred to PVDF membranes (Merck) at 25 V overnight at 4° C. Membranes were blocked (5% skimmed milk/TBST) for 90 minutes and probed with primary antibodies to various targets, including anti-phospho-AKT (Ser473; CST, 4060S), anti-AKT (CST, 4691S), anti-AXL (CST, 8661S), anti-EGFR (abcam, 52894), anti-FGFR4 (CST, 8562S), anti-E-cadherin (CST, 3195S), anti-phospho-ERK p44/p42 (Thr389, CST, 4377S), anti-ERK1/2 (CST, 9102S), anti-phospho-P70S6K (Thr389, CST, 9234S), anti-P70S6K (CST, 2708S) anti-CAV1 (CST, 3238S), anti-vimentin (CST, 3390S), anti-RAC1/CDC42 (CST, 4651S), anti-phospho-Rel A(Ser536, CST, 3033S), anti-Rel A (CST, 8242S), anti-phospho-STAT3 (Tyr705, CST, 9145S and Ser727, 9134T), anti-STAT3 (CST, 4909S), anti-p21/cip1 (CST, #2947S), anti-p27/kip1 (CST, #3686S), anti-c-PARP (Millipore, AB3565), anti-GPX4 (CST, 52455), anti-α-tubulin (Abcam, ab4074), anti-LAMP1 (CST, 9091S), anti-ULK1 (CST, 6439S), anti-ATG7 (CST, 2631S), anti-cyclin D1 (CST, 55506T), anti-P62 (CST, 8025S), anti-LC3A/B-I/II (CST, 12741S), anti-phospho-AMPKa (Thr172, CST, 50081S), anti-AMPKa (CST, 5831S), anti-phospho-acetyl-CoA carboxylase (Ser79, CST, 11818S), anti-acetyl-CoA carboxylase (CST, 3676S), anti-GAPDH (Santa Cruz, sc47724), and anti-β-actin (abcam, ab6276). Secondary horseradish peroxidase linked anti-rabbit IgG (GE healthcare, NA934V) and anti-mouse IgG (GE healthcare, NA931V) were used to detect specific protein bands using Luminata Crescendo Western HRP Substrate (Merck Millipore, Billerica, MA, #WBLUR0500). Bands were visualized and analysed using iBright FL1000 imaging system (Thermo Fisher).

Human phospho-kinase antibody array: Cell lysates from Huh-7/SR1 cells transfected for 96 hours with 30 nM of either siNC or siCAV1 (#2 and #4) or lipofectamine 2000 control were analysed using the Human Phospho-Kinase Antibody Array (R&D, ARY003B) according to manufacturer's instructions. A total of 200 ug of protein was loaded per sample. Candidate molecules were validated using western blot.

Enzyme-linked immunosorbent assay (ELISA): The PathScan Sandwich Phospho-Axl (panTyr) ELISA kit (Cell Signaling, Cat#7042) was used to detect phosphorylated levels of Axl (p-Axl). Cell lysates extracted as per protein extraction protocol previously mentioned were diluted with sample diluent and 0.5 µg of protein was plated into the kit's provided microwells and incubated at 37°C. After 2 h of incubation detection antibody was added for 1 h followed by HRP-Linked secondary antibody for 30 min at 37°C, according to the manufacturer's

protocol. Addition of TMB Substrate and STOP solution allowed for spectrophotometric determination of absorbance readings at 450 nm using the FLUOstar OPTIMA plate reader. Relative p-Axl expression was determined by normalising absorbance values to the Huh-7 DMSO control group.

Immunofluorescence immunocytochemistry (ICC) and immunohistochemistry (IHC): For ICC, cells (1.5 X 10^4 per well) were seeded in a 96-well plate overnight, washed with PBS and fixed with 4% paraformaldehyde for 15 minutes. After permeabilization (0.1% Triton X-100, 10 min) and blocking (1%BSA/10%NGS, 90 minutes), cells were incubated overnight with primary antibodies. For IHC, tissue sections from formalin fixed paraffin embedded samples were excised into 4 μM sections, dewaxed, and subjected to microwave mediated antigen retrieval in citrate buffer (pH = 6.0) for 20 min and thereafter processed similarly. For detection of CAV1 and pan-cytokeratin (AE1/AE3,) the cells or tissue sections were incubated overnight with either anti-CAV1 (CST 3238S, 1 in 400, rabbit) or anti-pan-keratin (CST 67306, 1 in 200, mouse) antibody in blocking buffer containing 3% BSA/PBS at 4° C. Alexa Fluor 594/488 conjugated secondary antibodies were used, followed by DAPI nuclear staining. Additional markers including F-actin by phalloidin-iFluor 488 reagent (abcam, #ab176753, 1 in 2000), α-smooth muscle actin (Sigma F3777, 1 in 100, mouse), vimentin (CST 3390S, 1 in 100, mouse), EGFR (Abcam ab52894, 1 in 100, rabbit), FGFR4 (CST 8562S, 1 in 100, rabbit), Snail (CST 3879S, 1 in 50, rabbit), Twist (CST, #46702S, 1 in 50, rabbit), E-cadherin (CST 3195S, 1 in 100, rabbit), ki67 (CST 9449S, 1 in 1000, mouse), P16INK4a (Abcam, ab211542, 1 in 100, rabbit), c-CASP3 (CST 9661, 1 in 100) and α-tubulin (CST #3873S, 1 in 1000, mouse), were stained according to manufacturer's instructions. Secondary antibodies included Alexa Fluor conjugated anti-rabbit IgG (Alexa Fluor 488 anti-rabbit IgG, A11008 and Alexa Fluor 594 anti-rabbit IgG, A11012) and anti-mouse IgG (Alexa Fluor 488 anti-mouse IgG, A11001 and Alexa Fluor 594 anti-mouse IgG, A11005) antibodies. The nuclei were stained with 4',6-diamino-2-phenylindole (DAPI; Sigma, #10236276001) in PBS. Automated imaging was performed using Cell Insight CX7 LZR High Content Analysis Platform captured at 20X objective (Thermo Fisher Scientific) and analysed by the built in HCS studio Cellomic Scan software 6.6.2 (Thermo Fisher Scientific). Additionally high-resolution images were captured at 60X magnification using C2+ confocal microscope (Nikon). Statistical significance was determined by unpaired student's t-test and one-way ANOVA.

Image analysis using HSC studio Cellomic Scan software: Images were captured at 20X magnification across 16 fields per well using Cell Insight CX7 LZR High Content Analysis Platform captured at 20X objective (Thermo Fisher Scientific) and analysed by the built in HCS

studio Cellomic Scan software 6.6.2 (Thermo Fisher Scientific). DAPI staining was used to identify nuclei, and cell segmentation was performed using the shape tool accounting for the differences in cell intensity and background. The Circ tool was applied to define individual cell boundaries, and the isodata tool, under general intensity measurement application, was selected to quantify the stained area of target protein. The stained area was calculated as the product of the average spot intensity and the total stained area per well. Data were normalised to the experimental control.

Cell Stiffness measurement: Atomic force microscopy (AFM, MFP-3D Origin, Asylum Research) (7) was used to assess cell stiffness in untransfected and miRNA/siRNA-transfected HCC cells. Cells were transfected with miRNA or siRNA (5 nM) and seeded at 2 X 10^4 cells/well on fibronectin-coated coverslips in triplicate in 12-well plates, then cultured for 72 h. After media removal, coverslips were transferred into petri dishes and covered with 1X PBS (Gibco). AFM indentations were applied by 200 μM chromium/gold-coated silicone-nitride triangular cantilever tips (17-kHz, 0.08N/m; Nano World PNP-TR-50). Triple indentations per cell at 2 μm/s were made until a 2-nN trigger force was reached with tip retraction at 10 μm/s. Three force curves per measurement were analysed using custom Igor Pro code to determine Young's moduli (7). At least 10 cells per well were analyzed. Statistical significance was assessed by unpaired student's t-test.

Fibronectin adhesion assay: 96-well plates were coated with fibronectin (10 µg/ml, Sigma, #ECM001-200) for 1 h at 37° C and rinsed with PBS. Cells (5 X $10^{4)}$ transfected with 5 nM of siRNAs were seeded in triplicate and incubated at 37° C for 20-25 minutes to allow adhesion. Non-adherent cells were removed, and complete growth media (100 µl) were added before incubation for 2 hours at 37° C. At the endpoint, the growth media were aspirated, and cells were incubated with CyQuant NF cell proliferation assay reagent (100 µl, Thermo Fisher Scientific, #C35006) for 1 h at 37° C. Fluorescence (excitation 485 nm, emission 530 nm) was measured using a spectrophotometer (Clariostar, BMG, Labtech). Statistical significance was determined by unpaired student's t-test was.

2D chemotaxis and invasion assay: Transfected cells (0.5 X 10^6/ml, 5 nM) were trypsinized and resuspended in DMEM plus 0.1% BSA. A total of 200 µl of the cell suspension was pipetted on to the top of the Boyden's chamber with/without Matrigel (BD Biosciences, #356234) coating, while fibronectin (10 µg/ml) in serum free DMEM was used as a chemoattractant in the bottom chamber. Chemotaxis assays were incubated for 48 and invasion assays for 72 h, at 37° C. Cells adhering to the underside of the upper chamber were

fixed in methanol (100%, v/v) and stained with crystal violet (0.1%). Cells were imaged at 20X magnification (Olympus IX71) and counted using Image J (1.52k, NIH, USA). Statistical significance was determined by either unpaired student's t-test or two-way ANOVA (>2 groups).

3D cell culture & collagen I invasion assay: Huh-7/SR1 cells were transfected with siNC/siCAV1 #2 (5 nM) or miR-NC/miR-7 (5 nM) for 24 h, then trypsinized and seeded (5 X 10^3 cells/well) in a round-bottom ultra-low attachment 96-well plate (Corning Costar, #7007) in complete growth medium. After 96 h, spheroids were photographed using a 10X objective (Olympus IX71). Bovine Collagen I (2mg/ml, Cultrex, Trevigen, #3443-005) was prepared on ice in EMEM (1X), L-glutamine (4 mM) and FBS (10%) and neutralized with sodium bicarbonate in PBS. Approximately half of the culture media was gently removed from each well, and to each 100 µl of collagen I gel (1 mg/ml) was added and then incubated at 37° C. After 2 h, once the gel solidified, 100 µl of growth media was added. Spheroids were imaged at 0, 24, 48 and 72 h under bright field using a 4X objective. Statistical significance was determined by unpaired student's t-test.

Cell titre assay, drug synergy and EC_{50} determination: To calculate the EC_{50} of sorafenib, lenvatinib, BGB324 (S2841, Selleck) and BLU9931(HY-12823, MedChemExpress), cells (5 x 10^3 /well) were seeded in 96-well plates and treated in triplicate with increasing concentration of sorafenib (0.1 – 100 µM) for 48 h, and lenvatinib (0.1 - 100 µM), BLU9931 (0.1 - 50 µM) or BGB324 (0.1 – 12 µM) for 72 h. DMSO-treated cells served as controls.

For determination of EC_{50} of miR-7, cells (5 X 10^3 /well) were reverse transfected with increasing doses (0.4 nM to 100 nM) of miR-7 or miR-NC control for 96h.

Cell viability was assessed using a CellTitre 96 AQueous One Solution Assay (Promega, #G3581), and absorbance was measured at 490 nm using spectrophotometer (Clariostar). EC_{50} values were calculated using four-parameter non-linear regression method in GraphPad Prism Software (v8.3).

Synergy between siRNAs/miRNAs and sorafenib/lenvatinib, as well as between sorafenib and BGB324 or lenvatinib and BLU9931, was assessed using Chou and Talaylay method (8). Cells were transfected with four high, four low, and EC_{50} doses of miRNAs or siRNAs for 48 h, followed by treatment with serial dilutions of sorafenib (48 h) or lenvatinib (72 h) at constant ratios. For small molecule inhibitor combinations, drugs were added simultaneously, with a 72-h treatment duration. Cell viability was measured at 490 nm using a spectrophotometer (Clariostar), and the fraction of dead cells (Fa) was calculated as Fa = 1 - OD. Data were input into CompuSyn (v3.0.1) to generate combination index plots and

isobolograms. Synergy was defined as a combination index (CI) < 1, additive effects as CI = 1, and antagonism as CI > 1 (8).

Cell viability assessment using the CellTitre Glo 2.0 assay: The CellTiter Glo 2.0 assay (Promega, #G9241) is a luminescence-based viability assay that quantifies ATP levels, which are primarily generated through glycolysis in cancer cells.

For viability assessment, cells were seeded at 5 X 10^3 per well in solid white flat-bottom 96-well plates (Corning, #3917). To evaluate the effects of siRNAs as single agents in resistant cells, reverse transfection was performed directly in white 96-well plates for 96 h. At endpoint, 10 uL of CellTiter Glo 2.0 reagent was added to 150 ul of cell culture media per well, without media replacement, and incubated in the dark for 10 minutes at room temperature to allow stabilization. Luminescence was then measured using a Clariostar spectrophotometer.

To assess synergy between siRNAs and TKIs, cells were reverse transfected for 48h, followed by drug treatment with sorafenib (48 h) or lenvatinib (72 h). The luminescence measurement process was repeated as described.

For drug-only treatment experiments, cells were plated at the same density, incubated overnight and treated with 10 μM TKI or DMSO control for 72 h. Luminescence was measured using Clariostar, as described above.

Drug dosing for synergy studies in pathway analysis: To assess synergy between sorafenib and siCAV1 #2 the following doses were used for viability assay and pathway analysis. The sorafenib-resistant cells were transfected with 10 nM siCAV1 #2 or siNC for 48h, followed by treatment with 10 μM sorafenib for 48 h. The same workflow was used for lenvatinib-resistant cells and the doses used for drug combinations were: 5 nM siCAV1 #2 or siNC and 8 μM lenvatinib. For evaluation of synergy between siAXL #47 and sorafenib in Huh-7/SR1 cells the following drug combinations were used: 10 nM siAXL #47 and 10 μM of sorafenib. To assess the synergistic effect between sorafenib and BGB324 in Huh-7 and Huh-7/SR1 cells, the cells were treated with 8 μM sorafenib and 1.5 μM BGB324 for 24 hours for ELISA assay and 4 hours for pathway analysis.

IncuCyte ZOOM system: The IncuCyte ZOOM system, featuring a Nikon phase contrast and fluorescence microscope (Nikon Plan Fluor 10X/0.30; cat no. 4464, OFN25 DIC L/NT), is housed within a humidified incubator and employs proprietary software (version 2018A, Essen BioScience) for automated acquisition and analysis of live cell images at regular intervals.

Cell proliferation and drug synergy validation: Using previously described experimental workflow, specific combinations of siRNAs/miRNAs or shRNAs and TKIs (sorafenib or lenvatinib or cabozantinib) were tested. Cells were plated at 5.0 X 10^3 per well in a 96-well plate and monitored bi-hourly over 4-7 days via time-lapse microscopy to assess growth rates under various treatment conditions. Growth metrices included percentage confluence and population doubling time, analysed using one-way repeated measure ANOVA for statistical significance.

IncuCyte scratch assay with Matrigel: Cells were seeded at 1X10^5 per well in an incucyte imagelock 96-well plate (Sartorius) and synchronized by 10 h serum starvation. A uniform scratch was made using the Wound-Maker (Essen BioSciences). After aspirating the media, 50 μl of Matrigel (1 mg/ml) was added to each well and incubated at 37° C for 30 mins. Once the Matrigel has solidified, 150 μl of media/chemoattractant were added to each well. The IncuCyte ZOOM then captured images every 2 h to monitor cell spreading across the scratch, measuring wound density over 3-7 days. Statistical significance was analysed by one-way repeated measure ANOVA.

Cell cycle analysis: HCC cells (5 X 10^5) were reverse transfected with either miR-7/miR-NC (5 nM) or siAXL#47/siNC (10 nM) for 48 h, followed by treatment with sorafenib (5 μM with miRNAs or 10 μM with siAXL) or lenvatinib (8 μM with miRNAs) or DMSO vehicle control for 24 h. Cells were fixed with ice cold 75% ethanol in FACs buffer, treated with RNAse and stained with propidium iodide (PI, BD pharmingen) for 30 minutes at room temperature. PI fluorescence (100,000 events) was analysed using an Accuri flow cytometer. Cell cycle distribution was quantified using FlowJo software (v7.6.1, BD Bioscences). Experiments were performed in triplicate on three independent days. Statistical significance was determined by one-way ANOVA.

Analysis of apoptosis: To determine the effect of siCAV1 #2 or miR-7 on apoptosis in Huh-7/SR1 and Huh-7/LR cells, HCC cells (5 X 10^5) were reverse transfected with either miR-7/siCAV1 #2 or miR-NC/siNC control (30 nM) for 72 h. To evaluate synergy with lenvatinib, Huh-7/LR cells were transfected with 5 nM siCAV1 #2/siNC or 10 nM miR-NC/miR-7 for 48 h, then treated with lenvatinib (8 μM) for an additional 48 h. For combination treatments in Huh-7/SR1, cells were transfected with 10 nM of siCAV1 #2/siAXL #47 or siNC for 48 h, followed by sorafenib (10 μM) treatment for 48 h. Both floating and live cells were collected and stained with Annexin V-FITC / PI according to manufacturer's protocol (BD pharmingen). Apoptotic cells were quantified by analysing 100,000 events using Accuri flow cytometer and FlowJo

software (v7.6.1, BD Biosciences). Experiments were done in triplicate on three independent days. Statistical significance was determined by one-way ANOVA.

Senescence-associated β-galactosidase activity: To assess the effect of miR-7 on senescence induction in Huh-7/SR1 and Huh-7/LR cells, HCC cells were transfected with miRNAs (5 nm) as described. Senescence-associated β-galactosidase activity was measured at 120 h post-transfection using the Senescence Detection Kit (Abcam, ab65351) according to manufacturer's instructions. Cells were photographed at 20X magnification, and the percentage of senescent cells was determined by counting cells with turquoise precipitate relative to unstained cells using Image J software. Statistical significance was determined by unpaired student's t-test.

FACs analysis to isolate CAV1+ cancer cells in sorafenib and lenvatinib treated Huh-7 cells: Huh-7 cells (2 X 10^6) were treated with sorafenib (10 μM) or lenvatinib (8 μM) for 72 h and 2 weeks, with drug replenished every 72 h for during the extended treatment. The treatment regimen followed a reverse order, starting with the 2-week treatment, followed by the 72-h treatment, and concluding at Day 0. Untreated cells from Day 14 (0 h) served as baseline controls. After treatment, cells were incubated with anti-CAV1 antibody on ice for 30 mins. Negative controls were prepared by omitting the primary antibody. Cells were then washed with PBS (4° C), fixed with 4% paraformaldehyde for 15 minutes, and incubated with Alexa Flour 488 anti-rabbit secondary antibody (1:1000) for 30 minutes at room temperature. After a final PBS wash, the cells were analysed by FACs.

Development of 3D mini tumour spheroids to investigate drug synergy: Huh-7/LR cells and LX2 cells were pre-transfected with 5 nm of miR-7 or miR-NC control for 24 h, then mixed at a 1:3 ratio (Huh-7/LR: LX2). The mixed cells were seeded in triplicate at a density of 5 X 10^3 cells per well in round-bottom ultra-low attachment 96-well plates (Costar #7007) or at 20 X 10^3 cells per well in flat-bottom ultra-low attachment 24-well plates (Costar #3473). Cells were cultured for 72 h to allow formation of mini tumour spheroids. After 72 h, Matrigel (1 mg/ml) was added to the 96-well plates. The spheroids were then treated with DMSO or lenvatinib (5 μM) for 72 h, and growth and invasion were monitored at 24 h intervals using the CellInsight CX7 spheroid assay. For gene expression analysis, both Huh-7/LR and LX2 cells were pre-transfected with 30 nM of miRNAs and the same workflow described above was used to establish tumour spheroids in 24-well plates. After 72 h, they were treated with DMSO or lenvatinib (8 μM) for 24 h and subsequently used for RNA extraction and gene expression analysis.

Gelatin Zymography: FITC-conjugated gelatin was synthesized to create gelatin-based gels. Conditioned media from 3D co-cultured tumour spheroids were pooled from three technical replicates to assess soluble factors, with protein concentration normalized to respective cell lysates. Equal amounts of protein (25 μg) were loaded to monitor MMP2 and MMP9 activity. Gels were visualised using iBright FL1000 imaging system (Thermo Fisher Scientific). Each experiment was performed on three independent days with three technical replicates.

Experiments using HCC patient-derived organoids (PDOs): The research was conducted with the ethical guidelines and standards according to Australian National Health and Medical Research Council and approved by the Sir Charles Gairdner Osborne Park Health Care group human research ethics committee approval number RGS0000000919. Informed consent was obtained from all participants involved in the study, and their anonymity and confidentiality were ensured throughout the research process. The study adhered to the ethical principles outlined on the Declaration of Helsinki (2008) and was undertaken in accordance with Australian Code for the responsible Conduct of Research (2018). Human HCC PDOs (PCB000087_02 & PCB000027_02) were sourced from Perkin's Biobank as described previously (9). To assess the efficacy of miRNA and siRNAs on PDO growth, organoids were dissociated into a single-cell suspensions using Tryple Express, counted and seeded at 5 x 10^3 per well in 20 μl of BME2 in round-bottom ultra-low attachment 96-well plates. Plates were incubated at 37° C for 30 minutes to allow BME2 to solidify into domes. Transfection cocktail was prepared by diluting miRNAs or siRNAs or controls (30 nM) in basal media (Advanced DMEM/F12 (Gibco) supplemented with 1X L-glutamine and 1X HEPES buffer sourced from Perkins Media Lab) and complexing with lipofectamine RNAiMAX (1 μl per well, Thermo Fisher Scientific) for 15 minutes at room temperature. The transfection cocktail (50 μl) was then added to each dome. The 96-well plates were then centrifuged at 500 rpm for 60 minutes at 25° C. After centrifugation, 80 μl of complete growth media was added, and organoids were incubated at 37° C with 5% CO_2. Growth was monitored daily, and viability was assessed on day 6 post-transfection using the ATP-based Cell Titer Glo 3D cell viability assay (Promega), according to manufacturer's instruction.

For drug treatment studies, the same workflow was followed, with drugs added 72 h post-dome formation, followed by centrifugation at 500 rpm for 30 minutes at 25° C. Growth and viability were assessed as above on day 6 post- treatment.

For RNA and protein analyses, multiple domes were prepared in 6-well plates, and miRNA/siRNAs were delivered using the same protocol in a final volume of 4 ml. Organoids

were harvested 24 h post-transfection for RNA extraction and gene expression analysis, and 96 h post-transfection for western blot analysis.

Evaluation of autophagy in TKI-resistant cells: Western blot analysis for autophagic markers: Western blotting was performed using to assess key markers of autophagic flux, including p62, LC3A/B-I/II, LAMP1, ULK1, ATG7 and the AMPKα pathway, in TKI-resistant cells under basal conditions, with comparisons to parental Huh-7 cells. To determine whether autophagic flux was inhibited or induced, the resistant cells were treated with chloroquine (CQ, 10 μM) for 24 h, followed by western blot analysis of the same autophagy-related markers.

To evaluate the effect of miR-7 overexpression and CAV1/AXL knockdown on autophagy, sorafenib- and lenavtinib-resistant cells were transfected with miRNAs or siRNAs or controls (10 nM) for 48 h, followed by 48 h of TKI treatment (10 μM) or 24 h CQ treatment (10 μM).

Lysosome visualization using LysoTracker Deep Red: Lysosomal content was visualized in parental Huh-7 and its resistant derivatives using LysoTracker Deep Red (Thermo Fisher Scientific, L7528) and live cell imaging. Both untransfected cells and cells transfected with 10 nM of siRNAs/miRNAs (10 nM, 120 h) were analysed. Following incubation, the culture media was removed, and cells were stained with LysoTracker Deep Red (60 nM) and Hoechst 33342 (1 µg/ml, Invitrogen) in phenol red-free complete culture medium to label lysosomes and nuclei, respectively. Cells were incubated for 30 minutes in a humidified incubator prior to imaging.

Measurement of lysosomal enzymatic activity by DQ Red BSA hydrolysis: To assess lysosomal enzymatic activity, DQ Red BSA (Thermo Fisher Scientific, D12051) was employed. This is a derivative of bovine serum albumin (BSA) conjugated to the BODIPY TR-X dye which selectively accumulates in endo-lysosomes and emits red fluorescence upon cleavage by lysosomal acid hydrolases, serving as a functional readout of lysosomal enzymatic activity.

To study the influence of miR-7 overexpression and CAV1/AXL knockdown on autophagic activity of lysosomes, Huh-7/SR1 and Huh-7/LR cells were transfected with miRNAs/siRNAs (10 nM, 120 h). Post-transfection, cells were washed once with DPBS and incubated with 100 µl of DQ-Red-BSA (10 µg/ml) in phenol red-free complete growth media for 12 hours at 37 °C with 5% CO2. After incubation, the substrate was removed, and the cells were fixed with 4% PFA in PBS, followed by DAPI staining for 30 minutes to visualise the nuclei.

Imaging and quantification: Imaging was performed at 20X magnification using the CellInsight CX7 high-content screening platform (Thermo Fisher Scientific). Image analysis was conducted using HCS Studio v. 6.6.2 Cell Analysis Software. Nuclear staining was used to identify cells. LysoTracker Deep Red and DQ-Red-BSA stained areas were visualised using red channel (594 nm). The lysosomal content and the lysosomal enzymatic activity (DQ-Red-BSA positive area) were calculated as the product of average red spot intensity and total stained area. Each experiment was performed on three independent days, with at least three replicates.

Assessment of intra-cellular delivery efficiency of miR-7 in TKI-resistant cells: To evaluate the cellular uptake efficiency of miR-7, Cy3-labelled-miRNA control mimic and Ambion miR-7 mimic (5 nM) were reverse transfected in Huh-7/SR1 cells. Delivery of miRNAs were assessed by visualisation of the cytoplasmic distribution of Cy3-labelled mimics. Hoechst 33342 (1 µg/ml, Invitrogen) was used to stain the nuclei. Cells were imaged at 4 h and 24 h using the High-Content Cell Insight CX7 system (Thermo Fisher Scientific). Fluorescence signals were detected using red channel (594 nM) for Cy3 and blue channel (350 nM) for Hoechst.

Statistical analysis: All *in vitro* experiments were carried out on three independent days with at least three technical repeats. For gene expression the bars represent average of relative target gene expression as fold changes ± standard deviation of one representative experiment. All other numerical data is represented as average ± standard deviation. Statistical significance was determined by unpaired student's t-test, one-way ANOVA, RM-ANOVA and two-way ANOVA as described under the result section by GraphPad prism software (v8.3). Significance was indicated as *$p<0.05$, **$p<0.01$, ***$p<0.001$ and ****$p<0.0001$.

References:

1. Kabir TD, Ganda C, Brown RM, Beveridge DJ, Richardson KL, Chaturvedi V, et al. A microRNA-7/growth arrest specific 6/TYRO3 axis regulates the growth and invasiveness of sorafenib-resistant cells in human hepatocellular carcinoma. Hepatology. 2018;67(1):216-31.
2. Sharma A, Seow JJW, Dutertre CA, Pai R, Bleriot C, Mishra A, et al. Onco-fetal Reprogramming of Endothelial Cells Drives Immunosuppressive Macrophages in Hepatocellular Carcinoma. Cell. 2020;183(2):377-94 e21.
3. Newman AM, Steen CB, Liu CL, Gentles AJ, Chaudhuri AA, Scherer F, et al. Determining cell type abundance and expression from bulk tissues with digital cytometry. Nat Biotechnol. 2019;37(7):773-82.
4. Wang XR, Park J, Susztak K, Zhang NR, Li MY. Bulk tissue cell type deconvolution with multi-subject single-cell expression reference. Nature Communications. 2019;10.
5. Ge SX, Son EW, Yao R. iDEP: an integrated web application for differential expression and pathway analysis of RNA-Seq data. BMC Bioinformatics. 2018;19(1):534.
6. Roy R, Winteringham LN, Lassmann T, Forrest ARR. Expression Levels of Therapeutic Targets as Indicators of Sensitivity to Targeted Therapeutics. Mol Cancer Ther. 2019;18(12):2480-9.
7. Chin IL, Hool L, Choi YS. Interrogating cardiac muscle cell mechanobiology on stiffness gradient hydrogels. Biomater Sci. 2021;9(20):6795-806.
8. Chou TC. Drug combination studies and their synergy quantification using the Chou-Talalay method. Cancer Res. 2010;70(2):440-6.
9. Nuciforo S, Fofana I, Matter MS, Blumer T, Calabrese D, Boldanova T, et al. Organoid Models of Human Liver Cancers Derived from Tumor Needle Biopsies. Cell Rep. 2018;24(5):1363-76.

Supplementary figures:

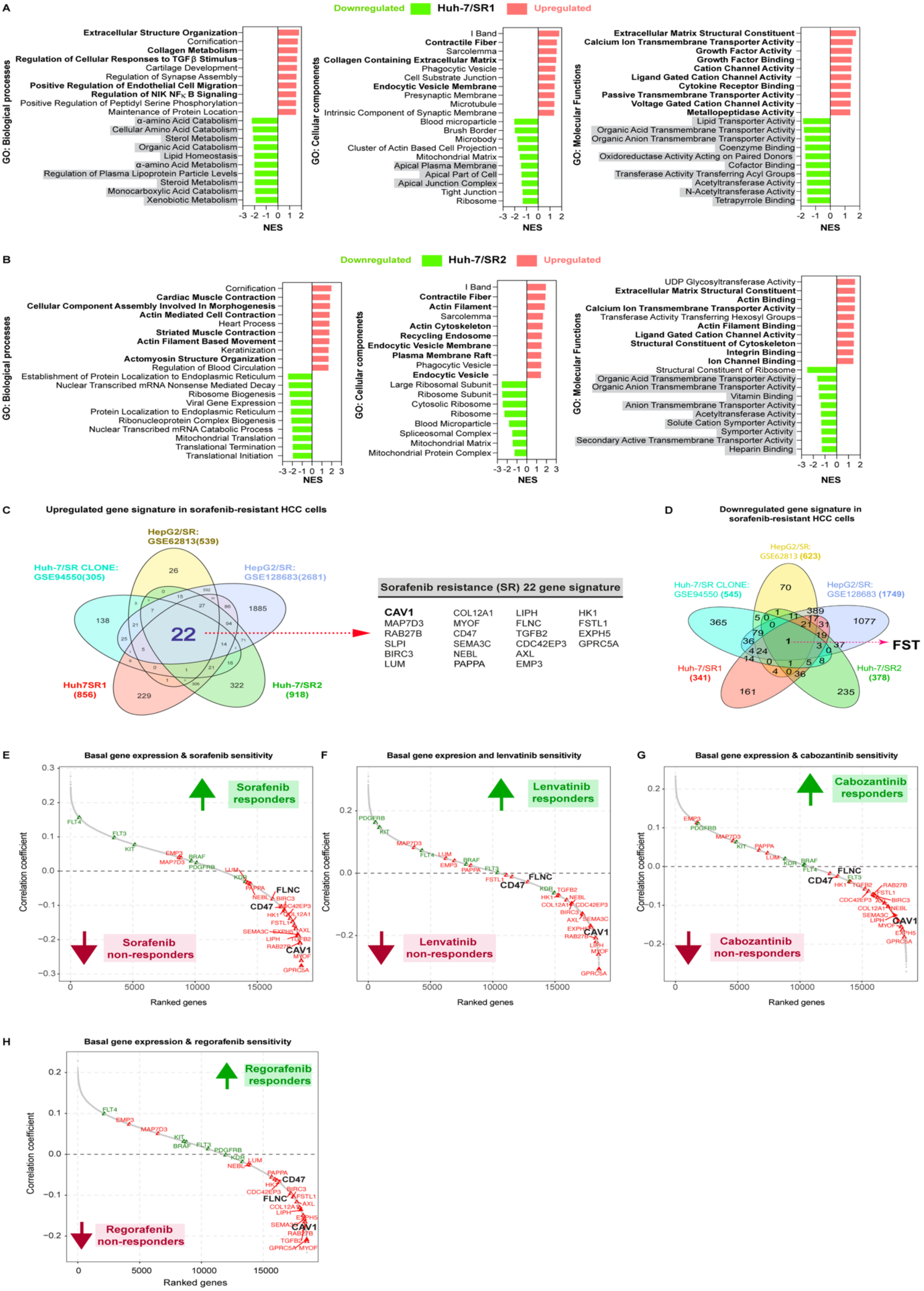

Suppl. Fig. S1: Evaluation of genes and pathways associated with the development of Pan-tyrosine kinase inhibitor resistance. Gene Ontology (GO) gene set enrichment analysis of biological processes, cellular components and molecular functions in Huh-7/SR1 **(A)** and Huh-7/SR2 **(B)** cells, displaying normalized enrichment scores (NES) at FDR q-value <0.25 (n = 3 per group). Gene overlap analysis of **(C)** upregulated and **(D)** downregulated genes in Huh-7/SR1, Huh-7/SR2, Huh-7 A7 clone (GSE94550) and sorafenib-resistant HepG2 cells (GSE62813 and GSE128683). Distribution of correlation coefficients between basal mRNA expression of 18,515 genes in 785 treatment naïve human cancer cell lines and drug sensitivity including **(E)** sorafenib, **(F)** lenvatinib, **(G)** cabozantinib and **(H)** regorafenib. Highlighted in green are validated drug targets. The genes associated with sorafenib resistance are highlighted in red and the 3 genes associated with pan-tyrosine kinase inhibitor resistance are highlighted in black.

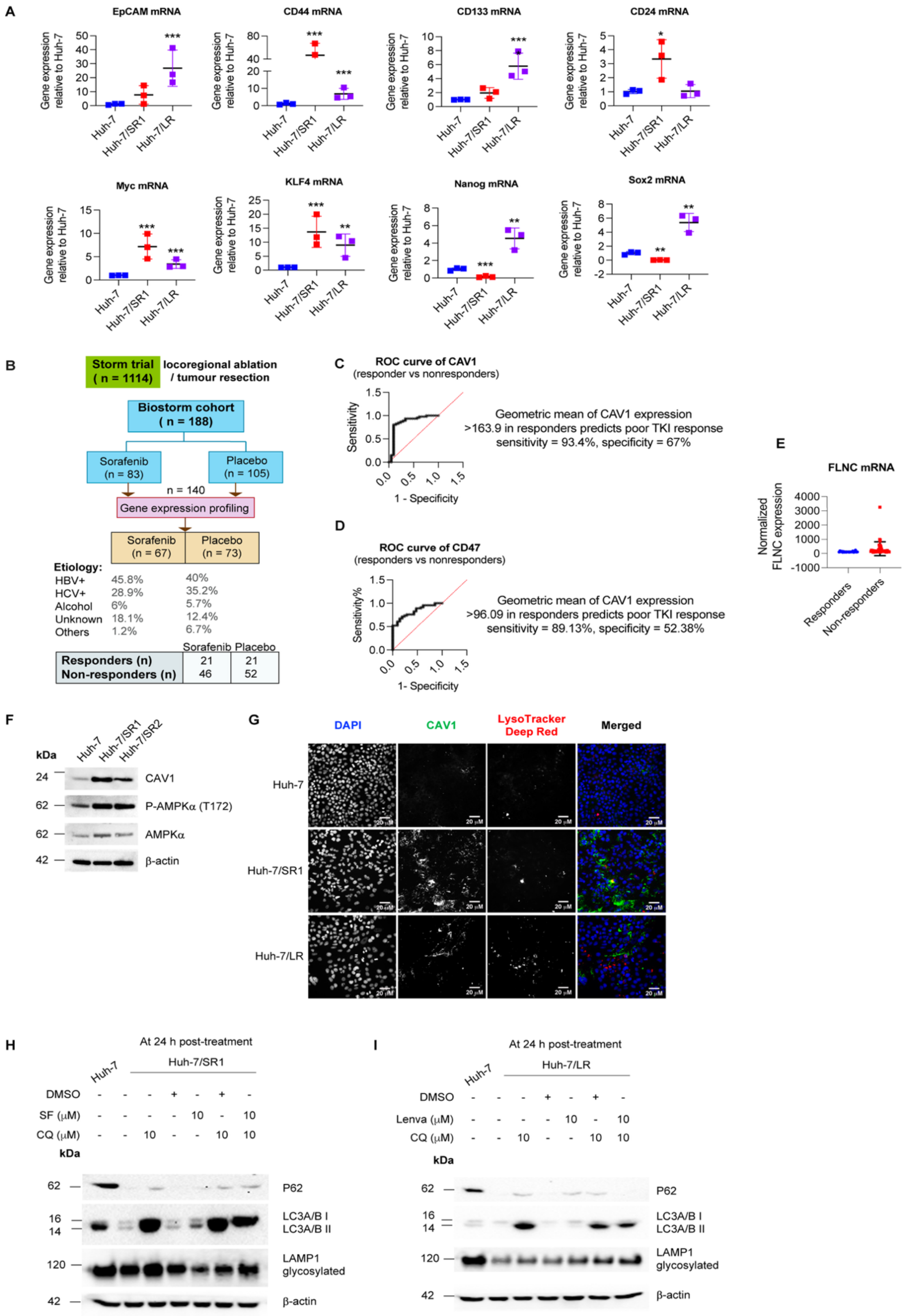
A
EpCAM mRNA
CD44 mRNA
CD133 mRNA
CD24 mRNA
Myc mRNA
KLF4 mRNA
Nanog mRNA
Sox2 mRNA
Gene expression relative to Huh-7
Huh-7
Huh-7/SR1
Huh-7/LR
B
Storm trial (n = 1114)
locoregional ablation / tumour resection
Biostorm cohort (n = 188)
Sorafenib (n = 83)
Placebo (n = 105)
n = 140
Gene expression profiling
Sorafenib (n = 67)
Placebo (n = 73)
Etiology:
HBV+ 45.8% 40%
HCV+ 28.9% 35.2%
Alcohol 6% 5.7%
Unknown 18.1% 12.4%
Others 1.2% 6.7%
Sorafenib Placebo
Responders (n) 21 21
Non-responders (n) 46 52
C
ROC curve of CAV1
(responder vs nonresponders)
Sensitivity
1 - Specificity
Geometric mean of CAV1 expression
>163.9 in responders predicts poor TKI response
sensitivity = 93.4%, specificity = 67%
D
ROC curve of CD47
(responders vs nonresponders)
Sensitivity%
1- Specificity
Geometric mean of CAV1 expression
>96.09 in responders predicts poor TKI response
sensitivity = 89.13%, specificity = 52.38%
E
FLNC mRNA
Normalized FLNC expression
Responders
Non-responders
F
kDa
Huh-7
Huh-7/SR1
Huh-7/SR2
CAV1
P-AMPKα (T172)
AMPKα
β-actin
G
DAPI
CAV1
LysoTracker Deep Red
Merged
Huh-7
Huh-7/SR1
Huh-7/LR
H
At 24 h post-treatment
Huh-7
Huh-7/SR1
DMSO
SF (μM)
CQ (μM)
kDa
P62
LC3A/B I
LC3A/B II
LAMP1 glycosylated
β-actin
I
At 24 h post-treatment
Huh-7
Huh-7/LR
DMSO
Lenva (μM)
CQ (μM)
kDa
P62
LC3A/B I
LC3A/B II
LAMP1 glycosylated
β-actin

Supp. Fig S2: Evaluation of cancer stemness, autophagy, and association of *CAV1*, *CD47* and *FLNC* gene expression in TKI resistance. **(A)** q-RT-PCR analysis of cancer stemness-associated markers (*EpCAM*, *CD44*, *CD133*, *CD24*, *Myc*, *KLF4*, *Nanog* and *Sox2*) in Huh-7/SR1 and Huh-7/LR cells (n = 3). GAPDH served as the internal control. Each data point represents the mean of three technical replicates per sample, with error bars representing ± SD. **(B)** Schematic representation of the Biostorm HCC cohort from the STORM trial, detailing patient selection for gene expression analysis. **(C, D)** ROC curve analysis of (C) *CAV1* and (D) *CD47* expression in sorafenib recipients. Gene expression values from responders (n = 21) were compared to non-responders (n = 46). The geometric mean values with the highest sensitivity and specificity are shown, demonstrating the predictive potential of *CAV1* and *CD47* for therapeutic response. **(E)** Differential expression of FLNC mRNA in sorafenib-treated HCC patients from the Biostorm HCC cohort; responders (n=21) vs non-responders (n=46). Statistical significance was determined by unpaired student's t-test. **(F)** Western blot analysis of the AMPKα signalling pathway in sorafenib-resistant Huh-7 cells. **(G)** Immunofluorescence immunocytochemistry analysis of CAV1 (green) and lysosomes (red, LysoTracker Deep Red) in Huh-7 parental and resistant derivatives. Western blot analysis for autophagy markers (P62, LC3A/B-I (16 kDa), LC3A/B-II (14 kDa), and LAMP1) in **(H)** Huh-7/SR1 and **(I)** Huh-7/LR cells following treatment with different drug combinations. Basal expression levels in sorafenib-resistant cells were compared to parental Huh-7 cells. β-actin was used as a loading control.

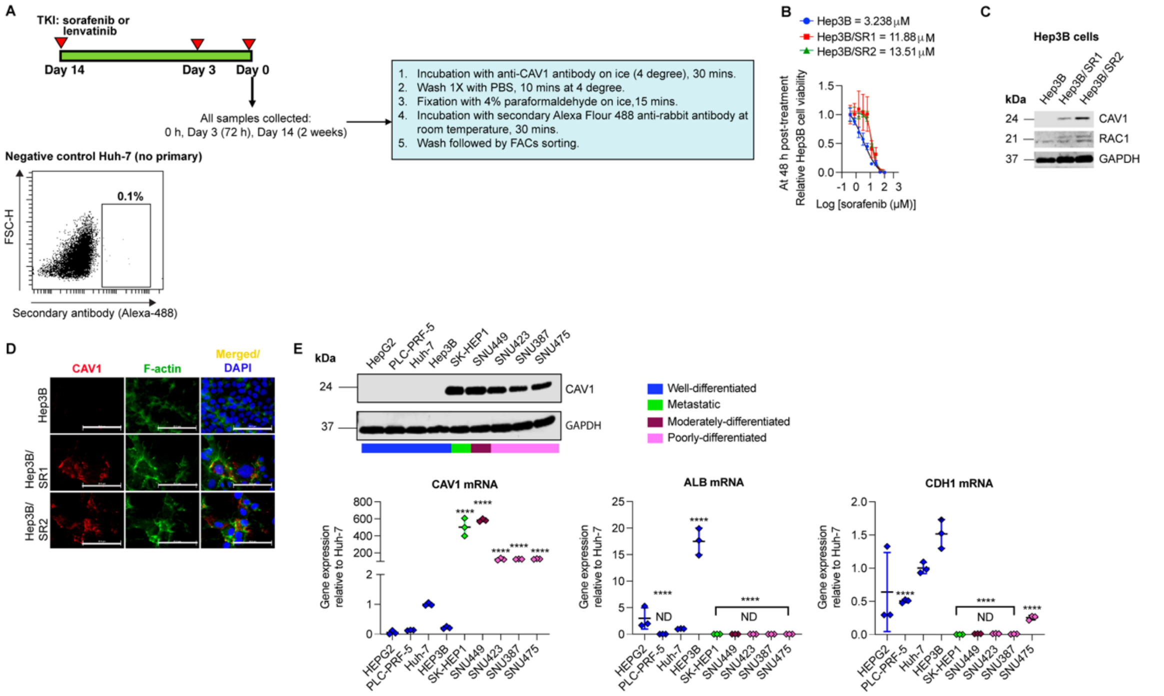

F

Association between high basal expression of CAV1 and pan-TKI resistance

HCC Cell line	Charateristics	Viral status	ALB mRNA	CDH1 mRNA	Sorafenib EC_{50} (μM)	Lenvatinib EC_{50} (μM)
HepG2	Well-differentiated (hepatocyte-like) [30]	-	+	+	4.214	25.86
PLC-PRF-5	Well-differentiated (hepatocyte-like) [30]	HBV+	+/-	+	7.067	15.95
Huh-7	Well-differentiated (hepatocyte-like) [14,30]	-	+	++	6.792	0.7751
Hep3B	Well-differentiated (hepatocyte-like) [14, 30]	HBV+	+++	++	3.238	7.698
SK-HEP1	Metastatic (endothelial-like) [31]	-	-	-	8.28	22.08
SNU449	Moderate (mesenchymal) [30]	HBV+	-	+/-	10.42	12.36
SNU423	Poorly-differentiated (mesenchymal) [30]	HBV+	-	+/-	10.22	10.96
SNU387	Poorly-differentiated (mesenchymal) [30]	HBV+	-	-	10.73	25.32
SNU475	Poorly-differentiated (mesenchymal) [30]	HBV+	-	+/-	15.18	20.14

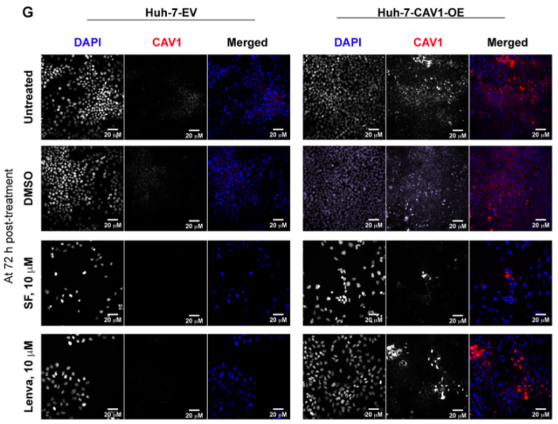

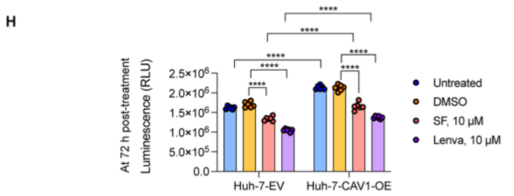

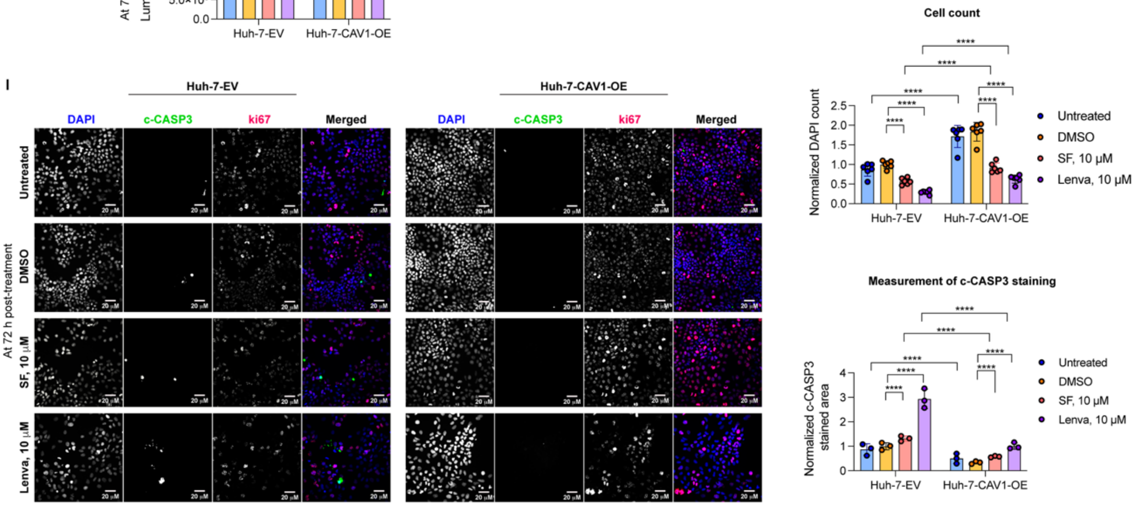

Suppl. Fig. S3: CAV1 overexpression promotes evasion of apoptosis in TKI-resistant cells. (A) Schematic of FACS analysis for CAV1+cancer cells in Huh-7 following chronic treatment with sorafenib or lenvatinib. Unstained control cells (no primary antibody) were included to account for background signal and autofluorescence. **(B)** Dose response curve for sorafenib in Hep3B cells and their resistant derivatives using a cell titer assay (n=3). **(C)** Western blot analysis of CAV1 and RAC1 signaling in sorafenib-resistant Hep3B cells (n=3). **(D)** Immunofluorescence staining for CAV1 (red) and F-actin (green) in Hep3B, Hep3B/SR1 and Hep3B/SR2 cells (n=3). **(E)** Western blot analysis of CAV1 and RT-qPCR analysis of *CAV1*, albumin (*ALB*) and e-cadherin (*CDH1*) mRNA, in HCC cell panel (n=3). **(F)** Overview of key phenotypic characteristics and TKI sensitivity across different HCC cell lines. **(G-I)** CAV1 expression, viability, proliferation and apoptosis analysis in Huh-7-EV (empty vector) vs Huh-7-CAV1-OE (CAV1 overexpression) cells following TKI treatments. Untreated cells and DMSO vehicle served as negative controls. **(G)** Immunofluorescence staining of CAV1. **(H)** CellTiter Glo viability assay measuring ATP production in drug-treated cells. **(I)** Immunofluorescence staining of ki67 (red, proliferation) and cleaved-caspase-3 (c-CASP3, green, apoptosis) in Huh-7-EV vs Huh-7-CAV1-OE cells. Images were acquired at 20X magnification across 16 fields per well using Cell Insight CX7 system. DAPI staining identified nuclei and cells segmentation was performed using the Shape tool. The isodata tool was applied to quantify c-CASP3 stained spots. c-CASP3-stained area was calculated as the product of the average spot intensity and the total stained area per well. Data were normalised to the control (siNC + DMSO). Each experiment was performed on three independent days with at least three technical replicates. RT-qPCR data were analysed using one-way ANOVA (vs. Huh-7 cells), while viability, DAPI count and c-CASP3 expression were analysed using two-way ANOVA with multiple comparisons. Statistical significance: ****$p<0.0001$. GAPDH served as the loading control. SF = sorafenib, Lenva = lenvatinib.

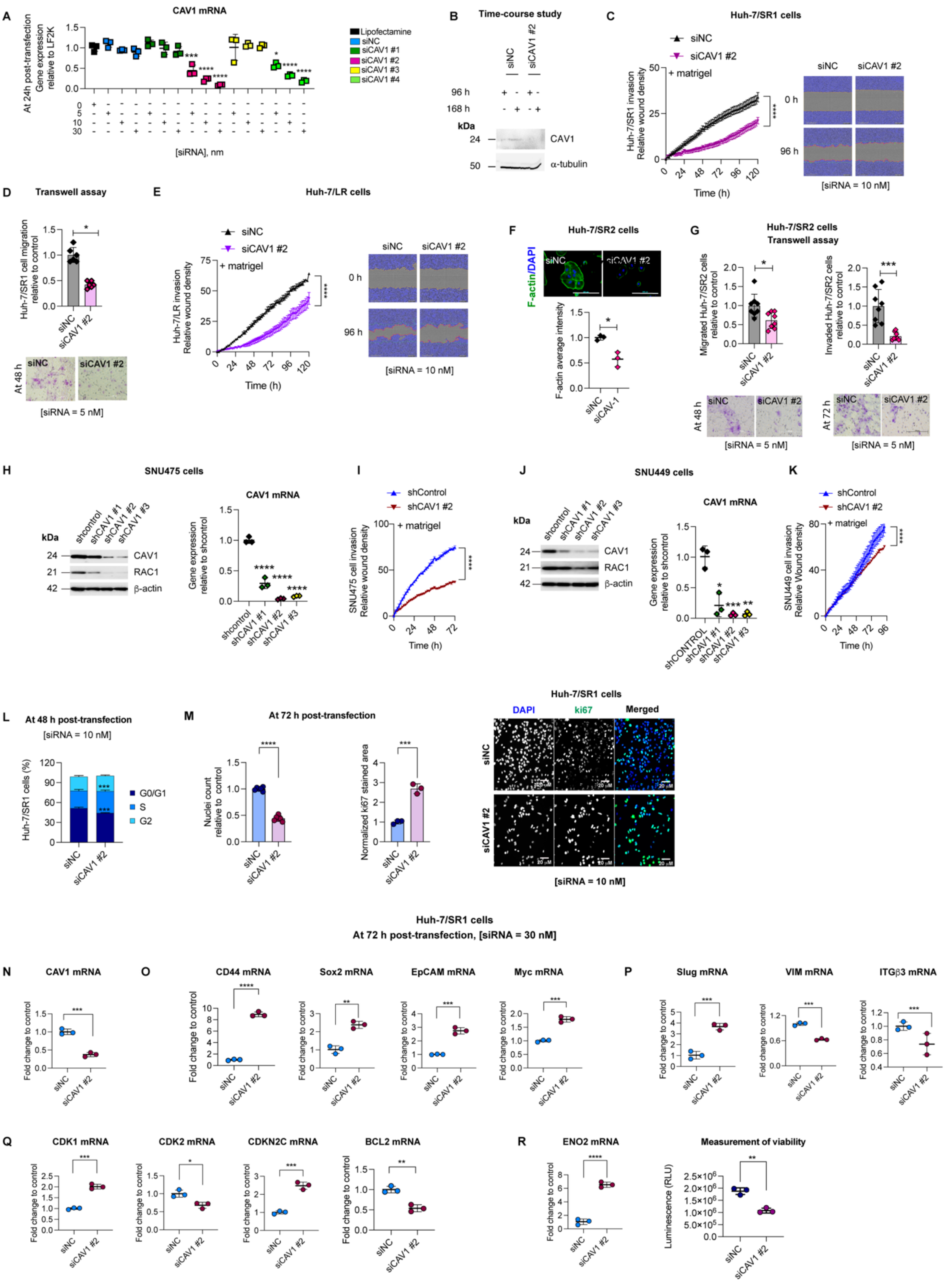

Suppl. Fig S4: Functional analysis of CAV1 signaling in TKI-resistant HCC cell lines. (A) RT-qPCR analysis of CAV1 knockdown efficiency using four different siRNAs in Huh-7/SR1 cells (n=3) at 24-h post-transfection. **(B)** Western blot analysis confirming sustained CAV1

knockdown in Huh-7/SR1 cells up to 168 h. α-tubulin served as the loading control. **(C)** Effect of CAV1 knockdown on Huh-7/SR1 cell invasion in Matrigel assessed by IncuCyte Zoom scratch assay (n=3). **(D)** Effect of CAV1 knockdown on chemotaxis of Huh-7/SR1 cells (n=3). **(E)** Effect of CAV1 knockdown on Huh-7/LR cell invasion in Matrigel evaluated by IncuCyte Zoom scratch assay (n=3). **(F-G)** Functional consequences of siCAV1 #2 in Huh-7/SR2 cells: **(F)** F-actin expression (n=3), **(G)** chemotaxis and invasion assessed via 2D transwell assays (n=8). **(H-K)** Effect of stable CAV1 knockdown in SNU475 and SNU449 cells (n=3): **(H, J)** Knockdown efficiency validated by western blot and RT-qPCR, and (**I, K)** effect on invasion in Matrigel using IncuCyte Zoom scratch assay. **(L)** Cell cycle analysis of CAV1-depleted Huh-7/SR1 cells at 48 h post-transfection. **(M)** Immunofluorescence staining for ki67 to assess cell proliferation. Images were captured at 20X magnification across 16 fields per well using Cell Insight CX7 system. DAPI staining identified nuclei, and cells segmentation was performed using the Shape tool. The isodata tool quanitified ki67 stained spots. Ki67-stained area was calculated as the product of the average spot intensity and the total stained area per well. Data were normalised to siNC control. **(N-R)** Analysis of dormancy-associated cancer stemness markers, EMT markers, G2/M checkpoint regulators and glycolysis markers in CAV1-depleted Huh-7/SR1 cells at 72 h post-siRNA transfection (n=3). **(N)** RT-qPCR validation of CAV1 knockdown. **(O)** RT-qPCR for cancer stem cell markers (*CD44, Sox2, EpCAM, Myc*). **(P)** RT-qPCR for EMT markers (Slug and its downstream targets *VIM* and *ITG*β*3*). **(Q)** RT-qPCR for G2/M checkpoint regulators (*CDK1, CDK2, CDKN2C, BCL2*). **(R)** RT-qPCR for glycolytic enzyme *ENO2* and functional glycolysis assessment via ATP production measured using the CellTiter Glo assay. Each experiment was performed on three independent days with at least three technical replicates. Time-course studies were analysed using one-way repetitive measure ANOVA. RT-qPCR studies comparing multiple conditions were analysed by one-way ANOVA, while comparisons between two groups were analysed by unpaired two-tailed student's t-test. Statistical significance: *<p0.05, **p<0.01, ***p<0.001, ****p<0.0001.

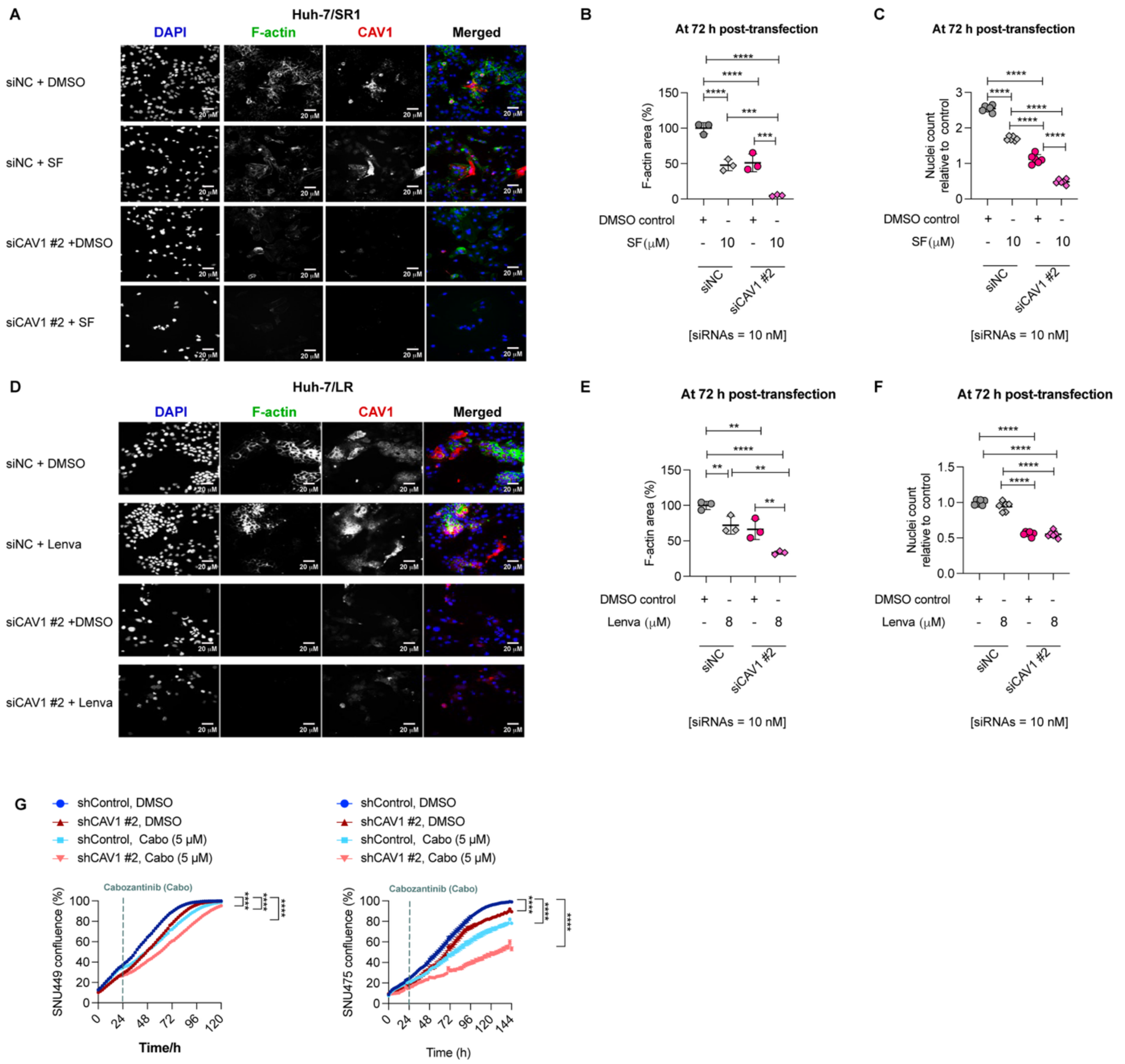

Suppl. Fig. S5: CAV1 knockdown enhances the cytotoxic effects of TKIs in TKI-resistant HCC cells. (A) Validation of CAV1 knockdown using immunofluorescence staining for nuclei, CAV1 and F-actin in Huh-7/SR1 cells treated with siCAV1 #2 alone or in combination with sorafenib (n=3). **(B)** Quantification of the effect of CAV1 knockdown on F-actin-stained area (%) in Huh-7/SR1 cells with or without sorafenib. Images were captured at 20X magnification using the Cell Insight CX7 high content analysis system with at least 8 fields per well. DAPI staining identified nuclei. F-actin-stained area was calculated as the product of the average F-actin intensity and the total stained area per well. Data were normalised to the control (siNC + DMSO vehicle). **(C)** Cell viability assessed by total nuclei count per well in Huh-7/SR1 cells treated with siCAV1 #2 alone or with sorafenib. Images were captured at 10X magnification across 16 fields per well using Cell Insight CX7 system. Data were normalised to the control (siNC plus DMSO vehicle). **(D)** Validation of CAV1 knockdown using immunofluorescence staining for nuclei, CAV1 and F-actin in Huh-7/LR cells treated with siCAV1 #2 alone or in

combination with lenvatinib (n=3). **(E)** Quantification of effect of CAV1 knockdown on F-actin-stained area (%) in Huh-7/LR cells with or without Lenvatinib, analysed as described above. **(F)** Cell viability monitored by total nuclei count per well in Huh-7/LR cells treated with siCAV1 #2 alone or with Lenvatinib, analysed as described above. **(G)** Growth curves illustrating the impact of shRNA-mediated CAV1 down-regulation in primary TKI-resistant HCC cell lines SNU449 and SNU475 on sensitivity to cabozantinib (Cabo) (n=3). Each experiment was performed on three independent days with at least three technical replicates. Growth curves and time course studies were analysed by one-way repetitive measure ANOVA. All other data was analysed by one-way ANOVA with multiple comparisons. **p<0.01, ***p<0.001, ****p<0.0001. SF = sorafenib, Lenva = lenvatinib, Cabo = cabozantinib.

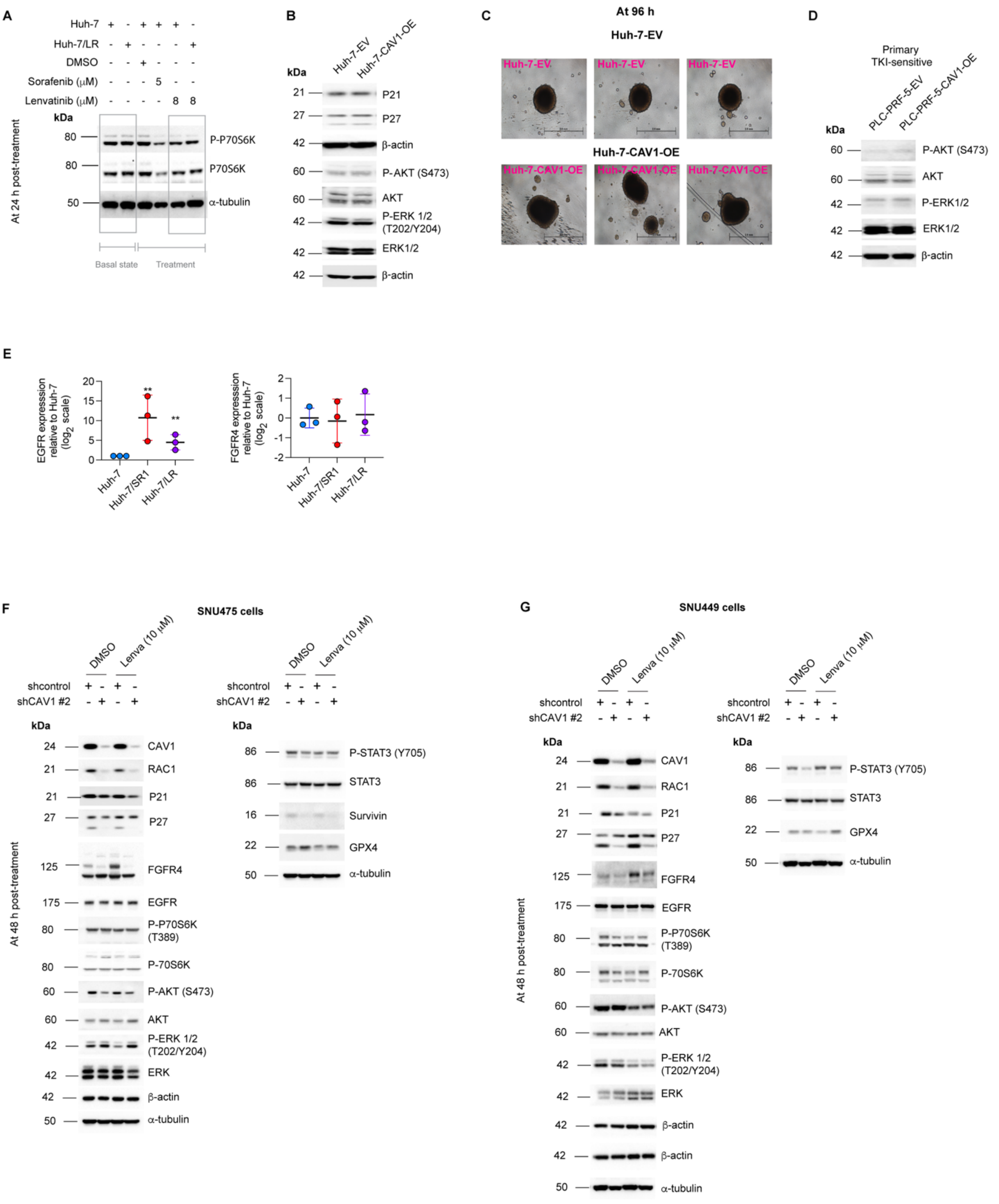

Suppl. Fig. S6: Exploring mechanisms driven by CAV1 in TKI-resistance: (A) Western blot analysis of P70S6K pathways in Huh-7 parental and Huh-7/LR cells under basal conditions and post-treatment (n=3). **(B)** Western blot showing the effect of CAV1 overexpression on p21, p27 and AKT/ERK signalling in Huh-7-CAV1-OE cells (n=3). **(C)** Impact of CAV1 overexpression on the growth of Huh-7-CAV1-OE cells in 3D culture assays (n=3). **(D)** Western blot evaluation of AKT/ERK signalling in PLC-PRF-5-CAV1-OE cells following CAV1 overexpression (n=3). **(E)** RT-qPCR analysis of *EGFR* and *FGFR4* mRNA

expression in Huh-7, Huh-7/SR1 and Huh-7/LR cells (n=3). **(F-G)** Western blot validation of CAV1 dependence in CAV1-depleted **(F)** SNU475 and **(G)** SNU449 cells treated with lenvatinib or DMSO control. All experiments were performed on three independent days with at least three technical replicates. Error bars represent ± SD. RT-qPCR data were evaluated by one-way ANOVA with multiple comparison (>2 groups). Significance is denoted as follows: **$p<0.01$. Lenva = lenvatinib. β-actin and α-tubulin were used as loading controls.

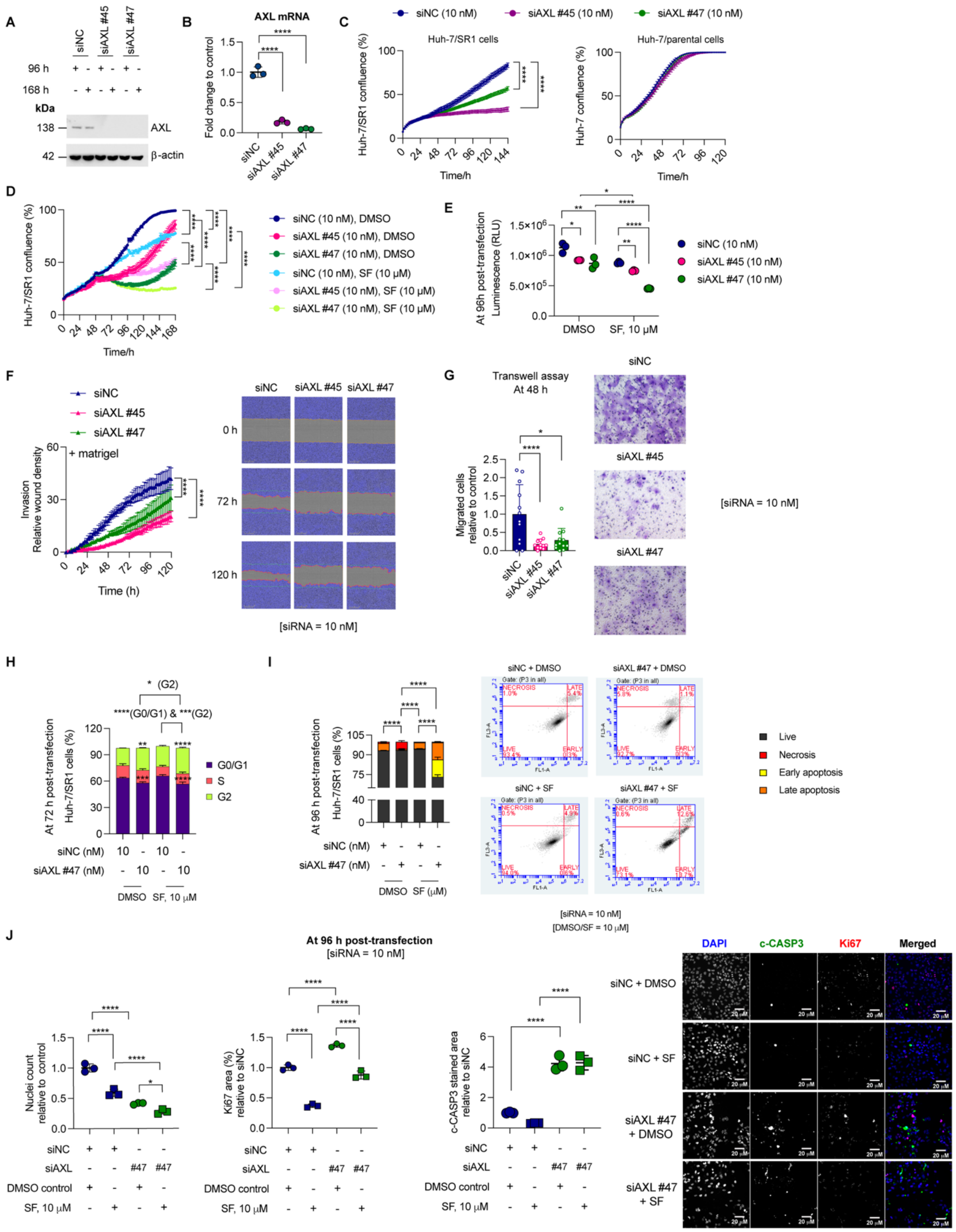

Suppl. Fig S7: Functional analysis of AXL signalling in sorafenib resistance. (A) Western blot analysis confirming sustained AXL knockdown in Huh-7/SR1 cells using two selective AXL targeting siRNAs (siAXL #45 and siAXL #47) up to 168 h. α-tubulin served as the loading

control. **(B)** RT-qPCR analysis of AXL knockdown efficiency in Huh-7/SR1 cells at 24-h post-transfection (n=3). **(C-D)** IncuCyte growth curve assays assessing **(C)** the impact of AXL inhibition on Huh-7/SR1 and parental Huh-7 cell proliferation (n=3) and **(D)** the restoration of sorafenib sensitivity following AXL knockdown in Huh-7/SR1 cells (n=3). **(E)** CellTiter Glo viability assay confirming synergistic inhibition of Huh-7/SR1 cell growth following combined AXL knockdown and sorafenib treatment (n=3). **(F-G)** Effect of AXL knockdown on **(F)** Huh-7/SR1 cell invasion in Matrigel, assessed by IncuCyte Zoom scratch assay (n=3), and **(G)** chemotaxis, measured in transwell assays (n=3). **(H)** Cell cycle analysis of Huh-7/SR1 cells under different treatment conditions by FACS (n=3). **(I)** Annexin V-FITC assay to measure apoptosis in Huh-7/SR1 cells under different treatment conditions (n=3). **(J)** Immunofluorescence staining for ki67 and c-CASP3 to assess cell proliferation and apoptosis in Huh-7/SR1 cells treated with siAXL ± SF. Images were captured at 20X magnification across 16 fields per well using Cell Insight CX7 system. DAPI staining identified nuclei, and cells segmentation was performed using the Shape tool. The isodata tool quanitified ki67 and c-CASP3 stained spots. Ki67/c-CASP3-stained area was calculated as the product of the average spot intensity and the total stained area per well. Data were normalised to control (siNC + DMSO). Each experiment was performed on three independent days with at least three technical replicates. Error bars represent ± SD. Time-course studies were analysed using one-way repetitive measure ANOVA. All other studies comparing multiple conditions were analysed by one-way ANOVA. Statistical significance: *<p0.05, **p<0.01, ***p<0.001, ****p<0.0001.

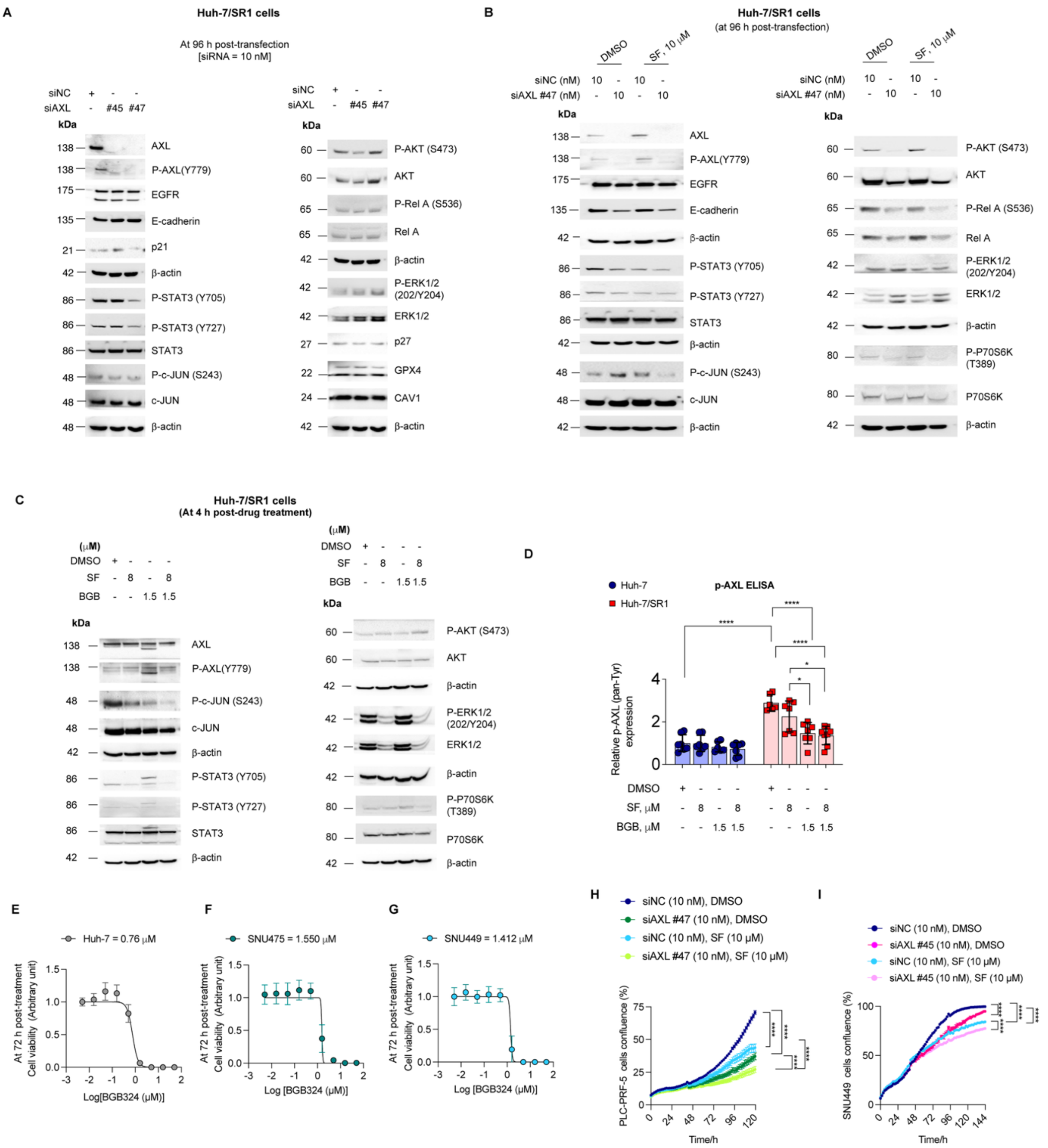

Suppl. Fig. S8: Combined AXL blockade and sorafenib treatment inhibits STAT3 and c-Jun signalling to overcome sorafenib resistance. (A) Western blot analysis of key signalling pathways following AXL knockdown in Huh-7/SR1 cells at 96 h post-transfection. Expression levels of AXL, EGFR, E-cadherin, cell cycle inhibitors (p21, p27) and survival pathways (phospho-STAT3, phospho-ERK, phospho-c-JUN, phospho-AKT and phospho-Rel) were assessed. β-actin served as the loading control. **(B)** Western blot analysis of AXL-depleted Huh-7/SR1 cells under different treatment conditions at 96 h post-transfection, with drug treatments administered for 24 h. Expression levels of growth factors, cell cycle regulators, and survival pathways, including phospho-P70S6K, were evaluated. β-actin

served as the loading control. **(C)** Western blot analysis of Huh-7/SR1 cells treated with different drug combinations for 4 h to identify pathways synergistically inhibited by BGB324 and sorafenib. **(D)** Pan-tyrosine kinase phospho-AXL ELISA demonstrating BGB324-mediated inhibition of naïve phosphorylated AXL. **(E-G)** Dose-response curves of the AXL inhibitor; BGB324, in **(E)** AXL-null Huh-7 cells, **(F)** AXL+ SNU475 and **(G)** AXL+ SNU449 cells, assessed by cell viability assay (n=3). **(H-I)** IncuCyte growth curve assays demonstrating the restoration of sorafenib sensitivity following AXL knockdown in AXL+ inherently sorafenib-resistant cells from (H) PLC-PRF-5 (well-differentiated HCC, n=3) and **(I)** SNU449 (moderately-differentiated HCC, n=3). Each experiment was performed on three independent days with at least three technical replicates. Error bars represent ± SD. Time-course studies and growth curve assays were analysed using one-way repetitive measure ANOVA. Statistical significance: *<p0.05 and ****p<0.0001.

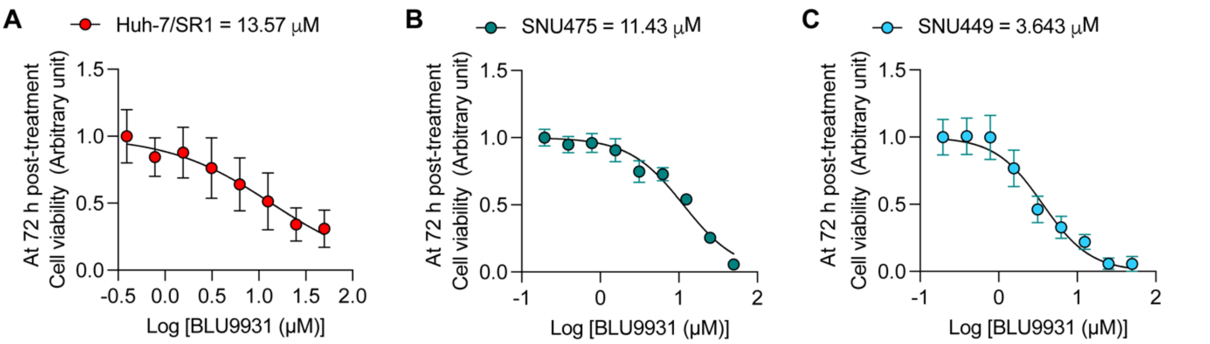

Suppl. Fig. S9: BLU9931 inhibits HCC cell growth in dose-dependent manner. (A-C) Dose response curves for BLU9931, a selective FGFR4 inhibitor, in **(A)** Huh-7/SR1, **(B)** SNU475 and **(C)** SNU449 cells following 72 h of drug treatment. Cell viability was assessed using a cell titer assay (n=3). Each experiment was performed on three independent days with at least three technical replicates. Error bars represent ± SD.

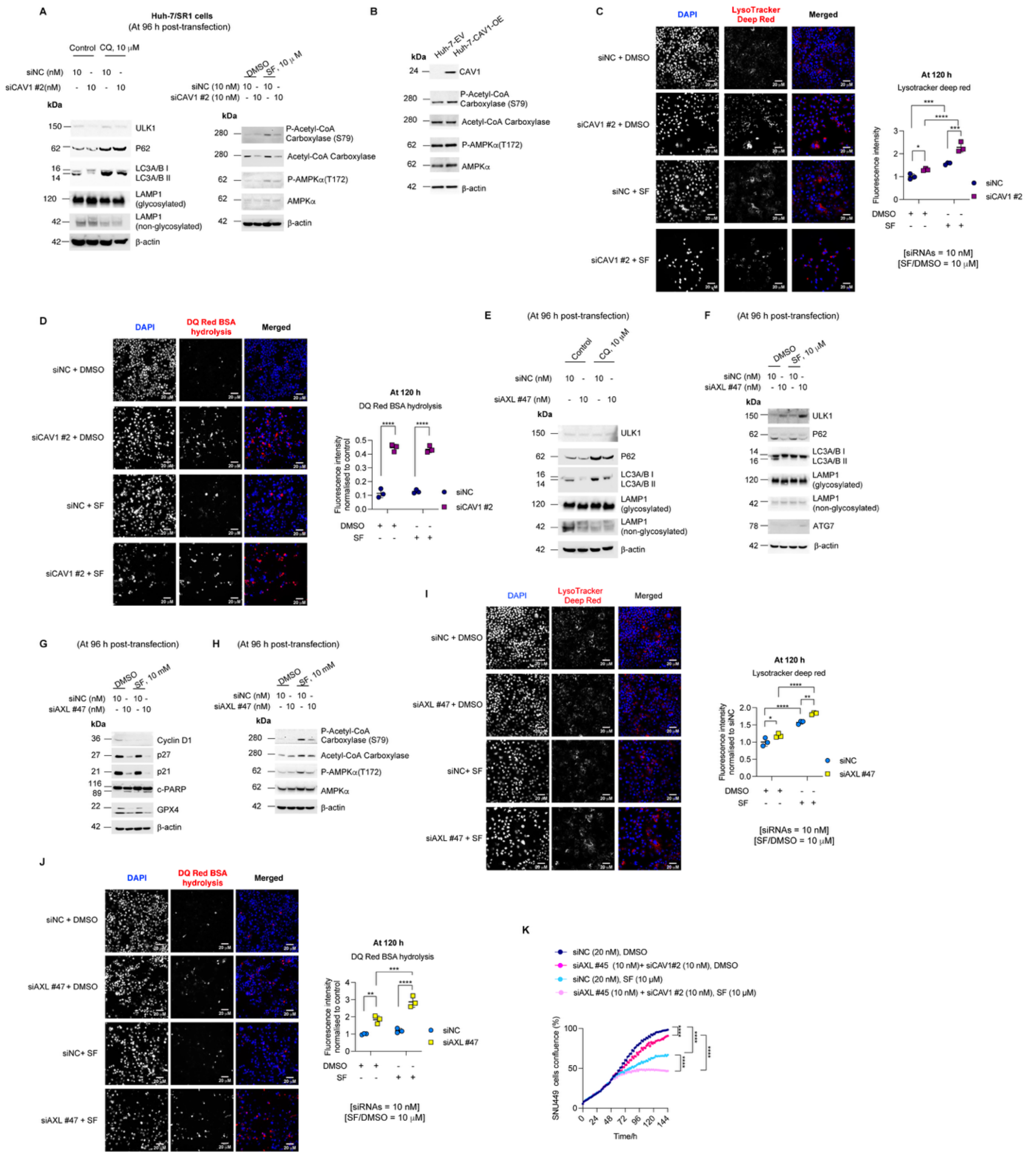

Suppl. Fig S10: Impact of CAV1 and AXL inhibition on autophagy in Huh-7/SR1 cells. **(A)** Western blot analysis of autophagy markers (P62, LC3A/B-I, LC3A/B-II, and LAMP1) and AMPKα signalling in CAV1-depleted Huh-7/SR1 cells under basal conditions and following 24 h chloroquine treatment (n=3). **(B)** Western blot analysis of AMPKα signalling following CAV1 overexpression in Huh-7-CAV1-OE cells. **(C)** Immunofluorescence analysis of lysosomes using LysoTracker Deep Red in CAV1-depleted Huh-7/SR1 cells with/without sorafenib. Images were acquired at 20X magnification across 16 fields per well using the Cell Insight CX7 system. DAPI staining identified nuclei, and segmentation was performed using the

Shape tool. The Isodata tool identified individual Lysotracker-positive puncta. The lysosomal content per cell was calculated as the product of average puncta intensity and total stained area per well. Data were normalised to control (siNC + DMSO). **(D)** Lysosomal enzymatic activity assessed by immunofluorescence for DQ Red BSA hydrolysis in CAV1-depleted Huh-7/SR1 cells treated with/without sorafenib. DQ Red BSA selectively enters the endo-lysosomes of live cells and fluoresces upon degradation by lysosomal acid hydrolases. Images were captured at 20X magnification across 16 fields per well using Cell Insight CX7 system. Lysosomal enzymatic activity was quantified as the product of average fluorescence intensity and total stained area. Data were normalised to control (siNC + DMSO). **(E)** Western blot analysis of autophagy markers and AMPKα signalling in AXL-depleted Huh-7/SR1 cells under basal conditions and following 24 h chloroquine treatment (n=3). **(F)** Western blot analysis of autophagy markers (P62, LC3A/B-I, LC3A/B-II, LAMP1, ULK1 and ATG7) in AXL-depleted Huh-7/SR1 cells with/without sorafenib treatment. **(G-H)** Western blot analysis of **(G)** survival factors (cyclin D1, p21, p27, c-PARP and GPX4) and **(H)** AMPKα signalling in AXL-depleted Huh-7/SR1 cells treated with/without sorafenib. **(I)** Immunofluorescence analysis of lysosomes using LysoTracker Deep Red in AXL-depleted Huh-7/SR1 cells with/without sorafenib. **(J)** Lysosomal enzymatic activity assessed by DQ Red BSA hydrolysis in AXL-depleted Huh-7/SR1 cells with/without sorafenib treatment. **(K)** IncuCyte growth curve assays assessing the effect of dual AXL and CAV1 inhibition using siRNAs on sorafenib sensitivity in SNU449 cells (n=3). Each experiment was performed on three independent days with at least three technical replicates. Error bars represent ± SD. Time-course studies and growth curve assays were analysed using one-way repetitive measure ANOVA. All other studies were analysed by one-way ANOVA with multiple comparisons. Statistical significance: *<p0.05, **p<0.01, ***p<0.001, ****p<0.0001.

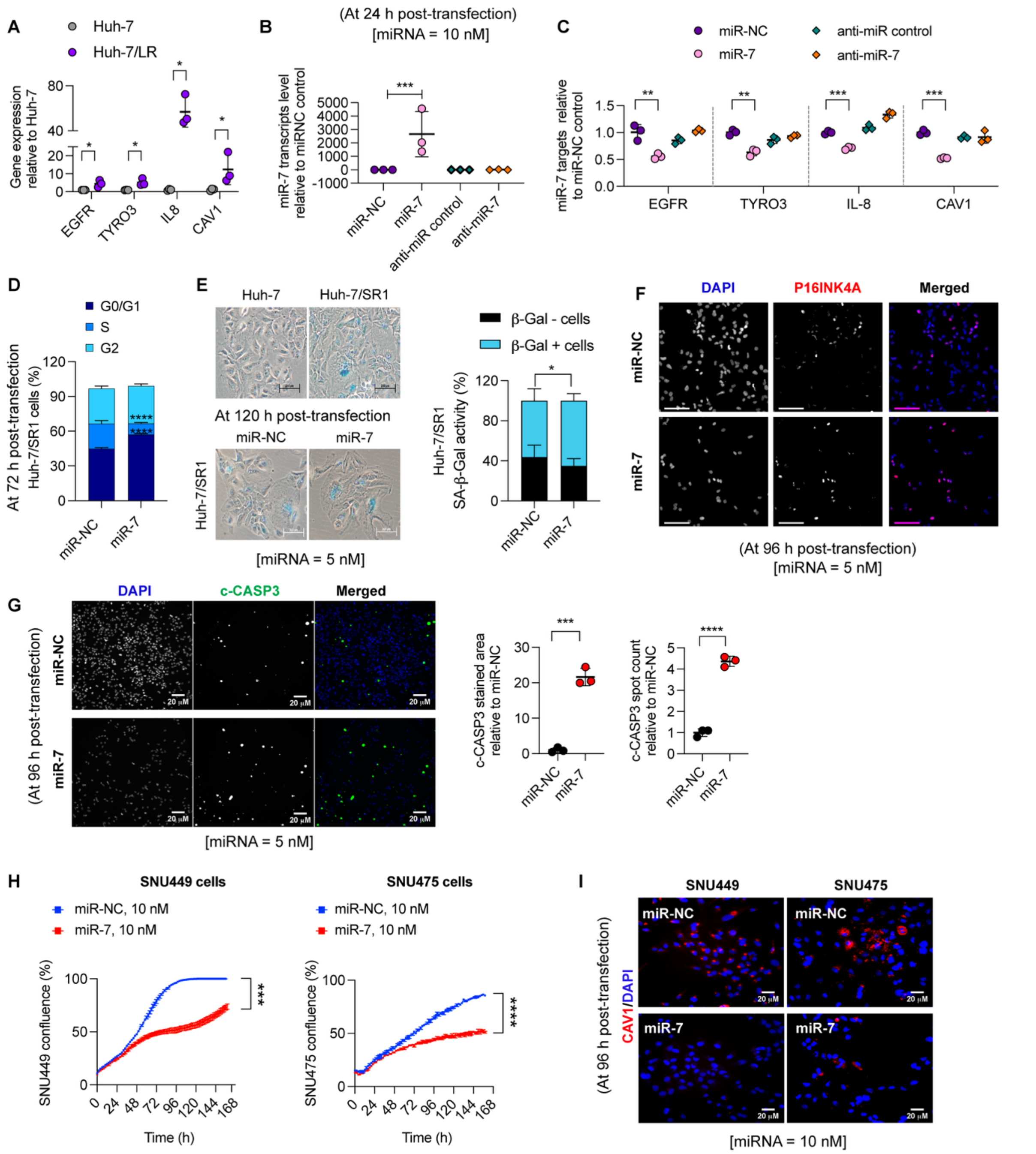

Suppl. Fig. S11: Impact of miR-7 on TKI-resistant HCC cell lines. (A) RT-qPCR analysis of basal expression of miR-7 targets (EGFR, *TYRO3*, *IL-8* and *CAV1* mRNA) in Huh-7/LR cells compared to Huh-7/parental. **(B)** Overexpression and under-expression of miR-7 transcripts in Huh-7/LR cells following transfection with synthetic miR-7 mimics or anti-miR-7. **(C)** Analysis of miR-7 mRNA targets in Huh-7/LR cells transfected with synthetic miR-7 mimics or anti-miR-7. **(D)** Cell cycle analysis to determine the effect of miR-7 on Huh-7/SR1 cells. **(E)** Evaluation of senescence in Huh-7/SR1 and parental Huh-7 cells under basal conditions and the impact of miR-7 on senescence in Huh-7/SR1 cells (n=3). **(F)** Immunofluorescence detection of p16 in miR-7 treated Huh-7/SR1 cells (n=3). **(G)** Apoptosis analysis of Huh-7/SR1

cells treated with miR-7 by immunofluorescence staining for c-CASP3 (n=3). **(H)** Growth curves showing the miR-7's growth inhibitory effect on SNU449 and SNU475 cells (n=3). **(I)** Immunofluorescence analysis of CAV1 protein expression in SNU449 and SNU475 cells treated with miR-7 (n=3). All experiments were performed on three independent days with at least three technical replicates. Error bars represent ± SD. Growth curves and time course studies were analysed by one-way repetitive measure ANOVA., while all other data were evaluated by an unpaired two-tailed student's t test. Significance is denoted as follows: *p<0.05, **p<0.01, ***p<0.001, ****p<0.0001.

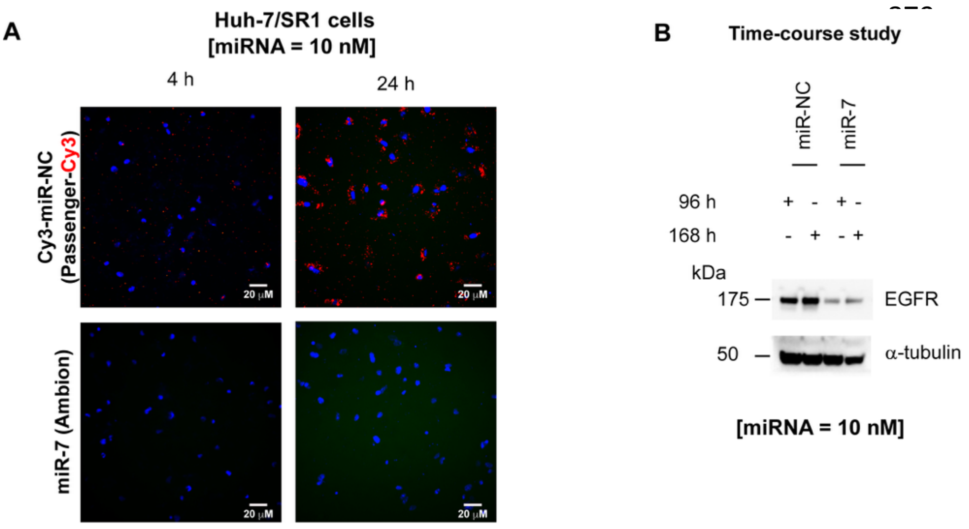

Suppl. Fig. S12: Evaluation of intracellular delivery Cy3 labelled miRNA control to demonstrate miRNA delivery in Huh-7/SR1 cells. (A) Immunofluorescence **analysis of** Cy3- labelled miRNA control mimics in Huh-7/SR1 cells at 4 h and 24 h time-points to assess the intracellular uptake. **(B)** Western blot confirming sustained suppression of EGFR protein levels in Huh-7/SR1 cells transfected with commercially available miR-7 (Ambion) up to 168 h. α-tubulin served as the loading control.

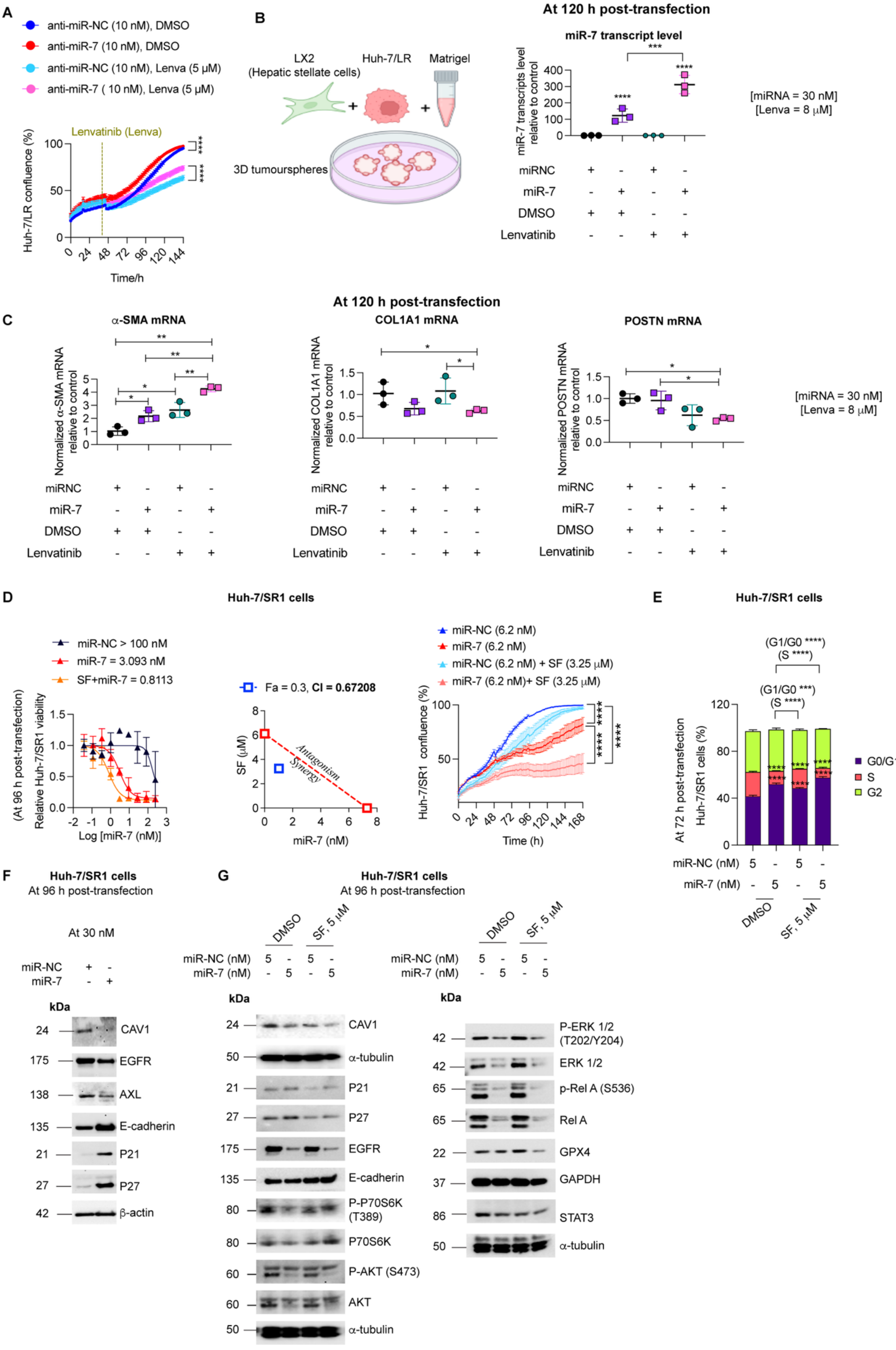
A
anti-miR-NC (10 nM), DMSO
anti-miR-7 (10 nM), DMSO
anti-miR-NC (10 nM), Lenva (5 μM)
anti-miR-7 (10 nM), Lenva (5 μM)
Lenvatinib (Lenva)
Huh-7/LR confluence (%)
Time/h
B
LX2
(Hepatic stellate cells)
Huh-7/LR
Matrigel
3D tumourspheres
At 120 h post-transfection
miR-7 transcript level
miR-7 transcripts level relative to control
miRNC
miR-7
DMSO
Lenvatinib
[miRNA = 30 nM]
[Lenva = 8 μM]
C
At 120 h post-transfection
α-SMA mRNA
Normalized α-SMA mRNA relative to control
COL1A1 mRNA
Normalized COL1A1 mRNA relative to control
POSTN mRNA
Normalized POSTN mRNA relative to control
miRNC
miR-7
DMSO
Lenvatinib
[miRNA = 30 nM]
[Lenva = 8 μM]
D
Huh-7/SR1 cells
miR-NC > 100 nM
miR-7 = 3.093 nM
SF+miR-7 = 0.8113
(At 96 h post-transfection) Relative Huh-7/SR1 viability
Log [miR-7 (nM)]
Fa = 0.3, CI = 0.67208
Antagonism
Synergy
SF (μM)
miR-7 (nM)
miR-NC (6.2 nM)
miR-7 (6.2 nM)
miR-NC (6.2 nM) + SF (3.25 μM)
miR-7 (6.2 nM)+ SF (3.25 μM)
Huh-7/SR1 confluence (%)
Time (h)
E
Huh-7/SR1 cells
(G1/G0 ****)
(S ****)
(G1/G0 ***)
(S ****)
At 72 h post-transfection Huh-7/SR1 cells (%)
G0/G1
S
G2
miR-NC (nM)
miR-7 (nM)
DMSO
SF, 5 μM
F
Huh-7/SR1 cells
At 96 h post-transfection
At 30 nM
miR-NC
miR-7
kDa
CAV1
EGFR
AXL
E-cadherin
P21
P27
β-actin
G
Huh-7/SR1 cells
At 96 h post-transfection
DMSO
SF, 5 μM
miR-NC (nM)
miR-7 (nM)
kDa
CAV1
α-tubulin
P21
P27
EGFR
E-cadherin
P-P70S6K (T389)
P70S6K
P-AKT (S473)
AKT
α-tubulin
P-ERK 1/2 (T202/Y204)
ERK 1/2
p-Rel A (S536)
Rel A
GPX4
GAPDH
STAT3
α-tubulin

Suppl. Fig. S13: Synergistic effects of miR-7 and lenvatinib/sorafenib in HCC cell lines. (A) IncuCyte growth curve assay showing the effects of anti-miR-7 alone and in combination with lenvatinib in Huh-7/LR cells (n=3). **(B)** Schematics for 3D tumour spheroid generation by co-culturing Huh-7/LR cells with LX2 hepatic stellate cells and Matrigel, and validation of miR-7 overexpression by RT-qPCR (n=3). **(C)** RT-qPCR analysis of LX2 cell activation markers; *α-SMA*, *COL1A1* and *POSTN* mRNA, under different treatment conditions. **(D)** Dose-response analysis of miR-7 alone and in combination with sorafenib, with synergy assessed via CI and isobologram analysis, F_a 0.3, CI = 0.67208 (n=3) and validation of synergy by incuCyte growth curve assay (n=3). **(E)** Cell cycle analysis of Huh-7/SR1 cells under various treatment conditions by FACS (n=3). **(F)** Western blot for miR-7 targets in Huh-7/SR1 cells (n=3). **(G)** Western blot analysis of the effect of miR-7 and sorafenib combination therapy on downstream signalling pathways in Huh-7/SR1 cells (n=3). . β-actin and α-tubulin were used as loading controls. All experiments were performed on three independent days with at least three technical replicates. Error bars represent ± SD. Growth curves and time course studies were analysed by one-way repetitive measure ANOVA., while all other data were evaluated by one-way ANOVA with multiple comparisons (>2 groups). Significance is denoted as follows: *p<0.05, **p<0.01, ***p<0.001, ****p<0.0001. Abbreviations: Fa= Fractional inhibition, SF = sorafenib.

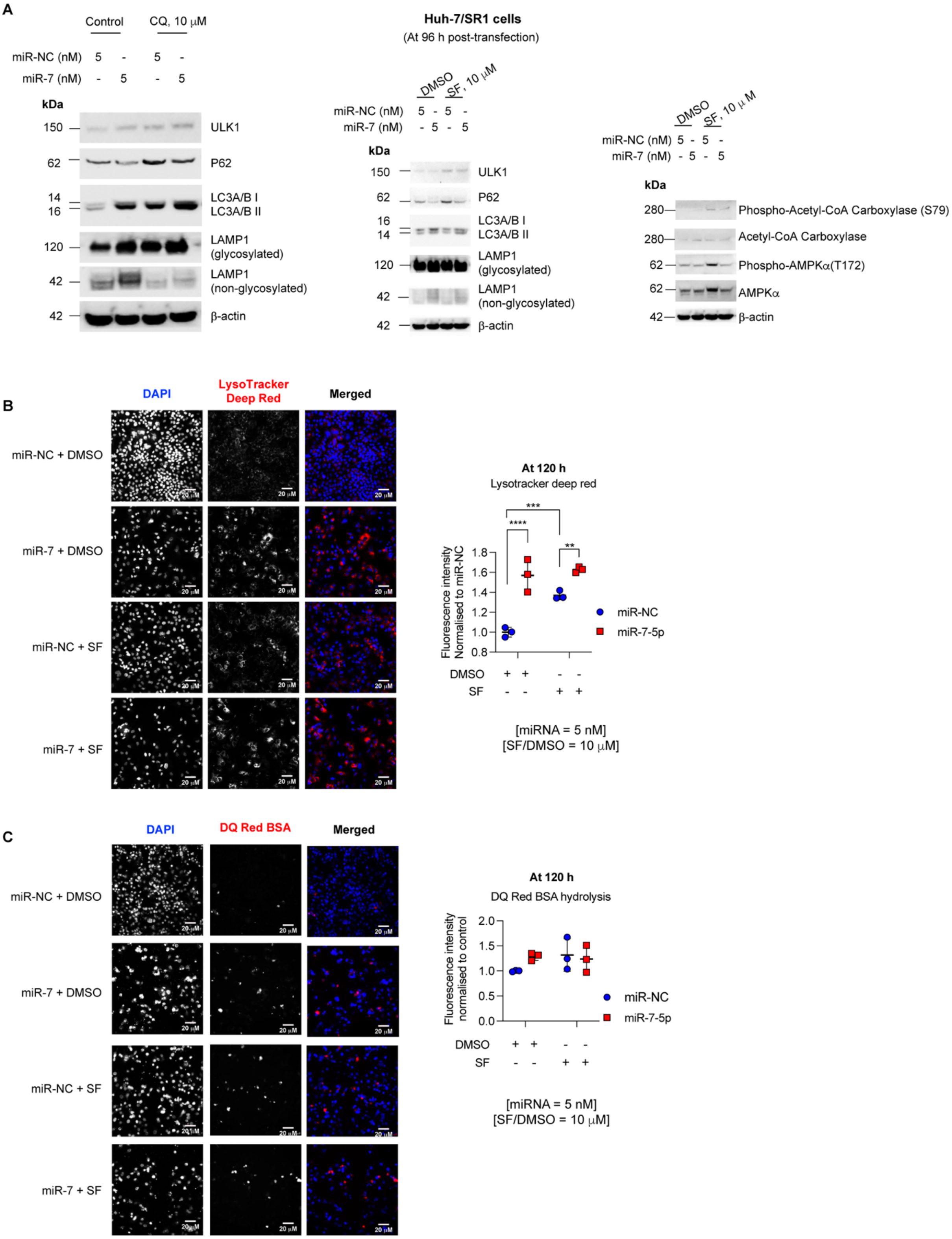

Suppl. Fig. S14: miR-7 mediated regulation of autophagy in Huh-7/SR1 cells. (A) Western blot analysis of autophagy markers (P62, LC3A/B-I, LC3A/B-II, and LAMP1) and AMPKα signalling in miR-7 transfected Huh-7/SR1 cells under basal conditions (n=3), after 24 h chloroquine treatment (n=3), and in combination with sorafenib (n=3). **(B)**

Immunofluorescence analysis of lysosomes using LysoTracker Deep Red in miR-7 treated Huh-7/SR1 cells with/without sorafenib. Images were acquired at 20X magnification across 16 fields per well using the Cell Insight CX7 system. DAPI staining identified nuclei and lysosomal content per cell was calculated as the product of average puncta intensity and total stained area per well. Data were normalised to control (miR-NC + DMSO). **(C)** Lysosomal enzymatic activity assessed by DQ Red BSA hydrolysis in miR-7 transfected Huh-7/SR1 cells treated with/without sorafenib. Lysosomal enzymatic activity was quantified as the product of average fluorescence intensity and total stained area. Data were normalised to control (miR-NC + DMSO). β-actin was used as loading controls. All experiments were performed on three independent days with at least three technical replicates. Error bars represent ± SD. Data were analysed by one-way ANOVA with multiple comparisons. Statistical significance: *<p0.05, **p<0.01, ***p<0.001, ****p<0.0001.

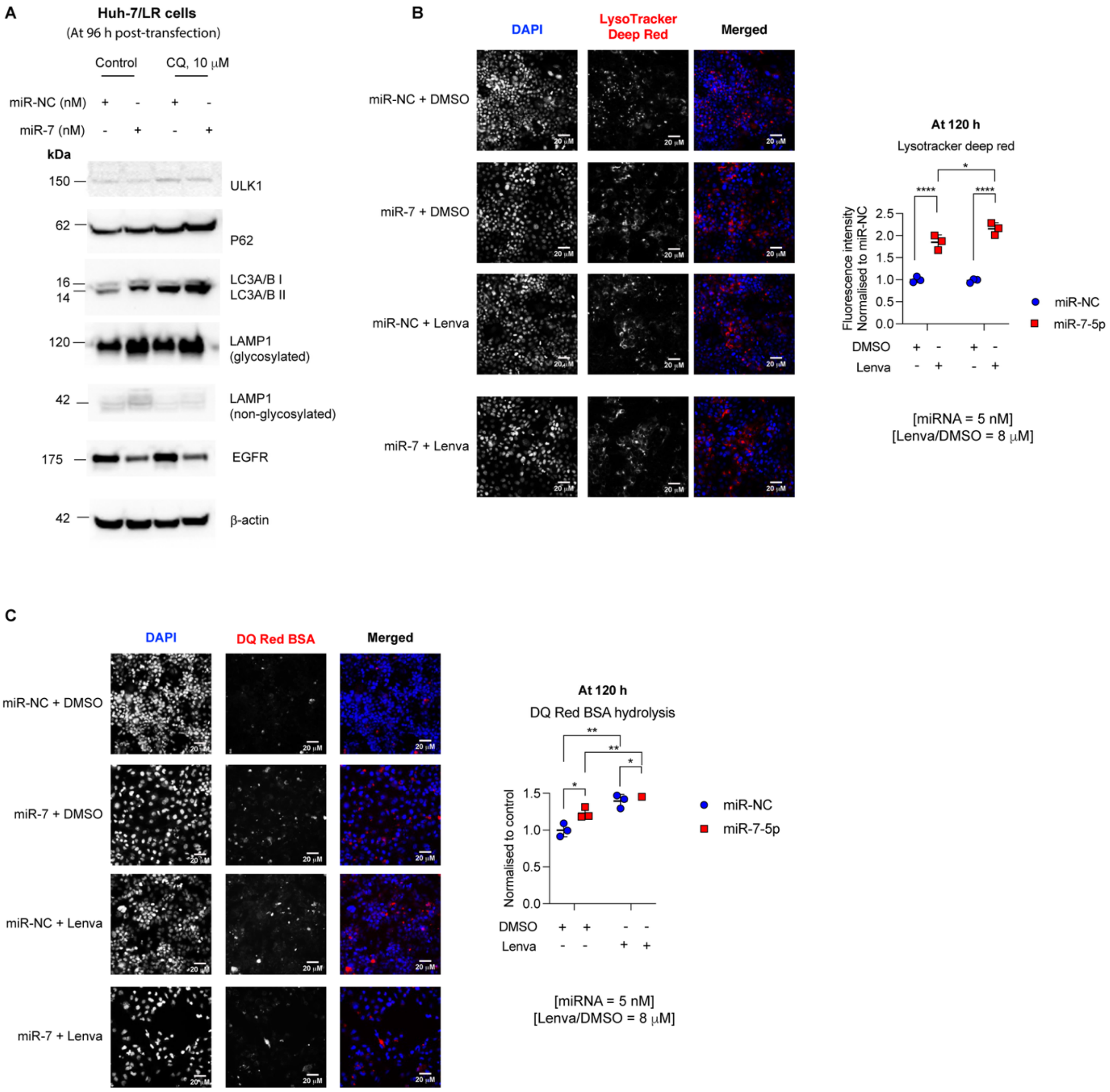

Suppl. Fig S15: miR-7 mediated regulation of autophagy in Huh-7/LR cells. (A) Western blot analysis of autophagy markers (P62, LC3A/B-I, LC3A/B-II, and LAMP1) and AMPKα signalling in miR-7 transfected Huh-7/LR cells under basal conditions and after 24 h chloroquine treatment (n=3). **(B)** Immunofluorescence analysis of lysosomes using LysoTracker Deep Red in miR-7 treated Huh-7/LR cells with/without lenvatinib. Images were acquired at 20X magnification across 16 fields per well using the Cell Insight CX7 system. DAPI staining identified nuclei and lysosomal content per cell was calculated as the product of average puncta intensity and total stained area per well. Data were normalised to control (miR-NC + DMSO). **(C)** Lysosomal enzymatic activity assessed by DQ Red BSA hydrolysis in miR-7 transfected Huh-7/LR cells treated with/without lenvatinib. Lysosomal enzymatic activity was quantified as the product of average fluorescence intensity and total stained area. Data were normalised to control (miR-NC + DMSO). β-actin was used as loading controls. All experiments were performed on three independent days with at least three technical replicates. Error bars represent ± SD. Data were analysed by one-way ANOVA with multiple comparisons. Statistical significance: *<p0.05, **p<0.01, ***p<0.001, ****p<0.0001.

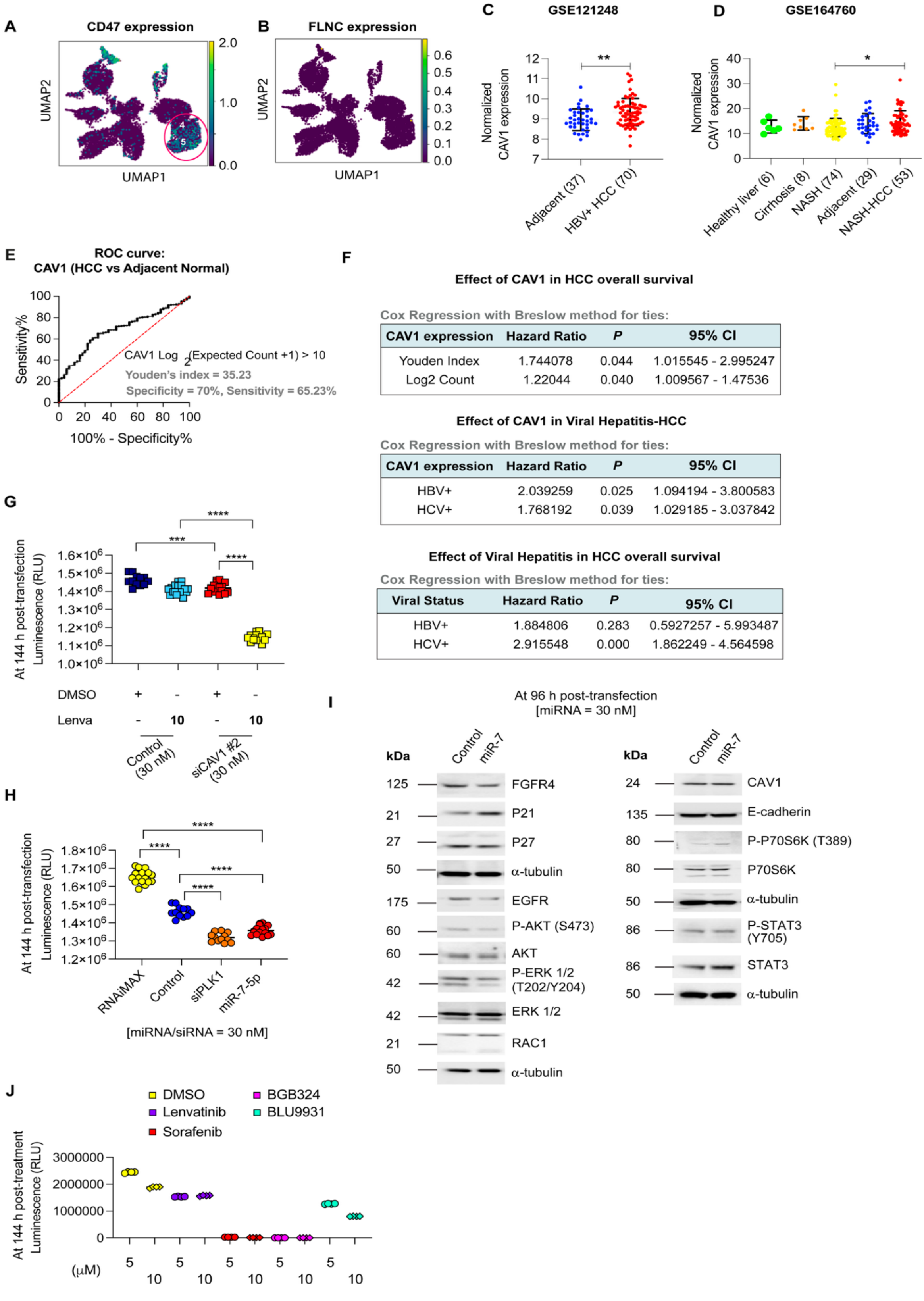

Effect of CAV1 in HCC overall survival

Cox Regression with Breslow method for ties:

CAV1 expression	Hazard Ratio	*P*	95% CI
Youden Index	1.744078	0.044	1.015545 - 2.995247
Log2 Count	1.22044	0.040	1.009567 - 1.47536

Effect of CAV1 in Viral Hepatitis-HCC

Cox Regression with Breslow method for ties:

CAV1 expression	Hazard Ratio	*P*	95% CI
HBV+	2.039259	0.025	1.094194 - 3.800583
HCV+	1.768192	0.039	1.029185 - 3.037842

Effect of Viral Hepatitis in HCC overall survival

Cox Regression with Breslow method for ties:

Viral Status	Hazard Ratio	*P*	95% CI
HBV+	1.884806	0.283	0.5927257 - 5.993487
HCV+	2.915548	0.000	1.862249 - 4.564598

Suppl. Fig. S16: Evaluation of drug targets in HCC. Spatial distribution of (A) CD47 and (B) FLNC in recurrence-associated hepatocyte clusters in HCC. Expression profile of CAV1 in HCC with different etiology: **(C)** GSE121248; (HBV+ HCC, n = 70 versus adjacent, n = 37),

and **(D)** GSE164760; NASH-associated HCC (HCC; n = 53 versus adjacent; n = 29, healthy liver; n =8, cirrhosis; n = 8 and NASH; n = 74). **(E)** Receiver operating characteristic (ROC) curve to calculate Youden Index of CAV1 in patients from the TCGA HCC cohort by using CAV1 expression values in adjacent normal and HCC tissues. **(F)** Cox Regression analysis of the TCGA HCC cohort using Breslow method to determine effect of CAV1 on overall survival of HCC patient and viral hepatitis + HCC, and the effect of viral etiology on HCC outcome. **(G)** 3D cell viability assay assessing lenvatinib sensitivity after CAV1 knockdown in HCC PDOs. **(H)** 3D cell viability assay evaluating the effect of miR-7 on HCC PDO growth. **(I)** Western blot of signalling pathways following miR-7 overexpression in HCC PDOs. **(J)** 3D cell viability assay evaluating the dose-response effects of TKIs; lenvatinib, sorafenib. BGB324 and BLU9931 on HCC PDO growth. For siRNA experiments, RNAiMAX was used as a lipid control, and non-targeting siRNA served as the lead control. All experiments were performed on three independent days with at least three technical replicates. Error bars represent SD. Data were analysed by one-way ANOVA with multiple comparisons (>2 groups) or unpaired two-tailed student's t-test. Significance is denoted as follows: *p<0.05, **p<0.01, ***p<0.001, ****p<0.0001. Abbreviations: Lenva = Lenvatinib.

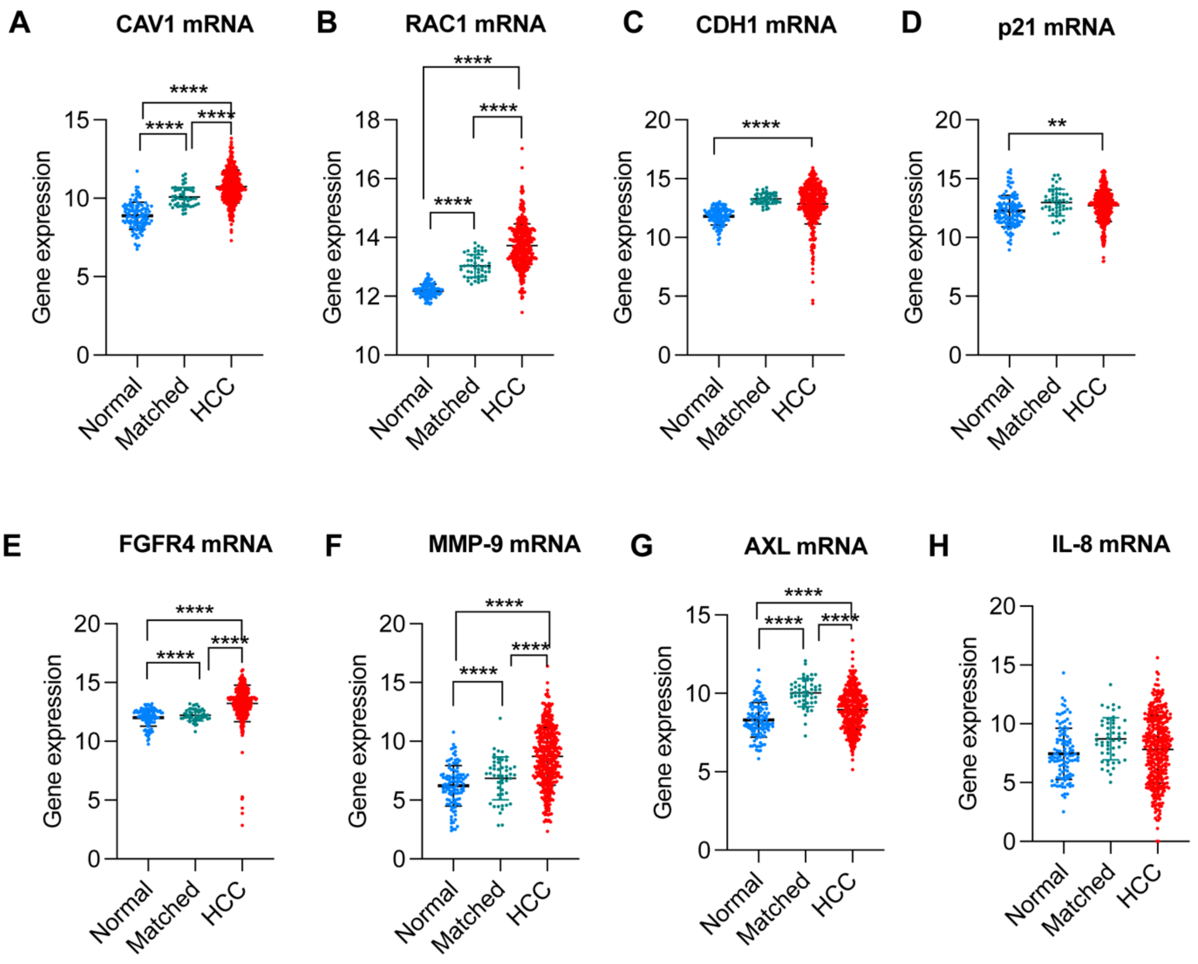

Normal = Normal Liver (GTEX, n = 110)
Matched = Adjacent normal, HCC tissue (TCGA LIHC, n = 50)
HCC = HCC tissue (TCGA LIHC, n = 369)

Suppl. Fig. S17: Analysis of gene targets of the CAV1 pathway in TCGA HCC cohort. Expression levels of **(A)** *CAV1*, **(B)** *RAC1*, **(C)** *CDH1*, **(D)** *p21*, **(E)** *FGFR4*, **(F)** *MMP-9*, **(G)** *AXL* and **(H)** *IL-8* in the TCGA HCC cohort (Matched or adjacent normal = 50 and HCC = 369) versus GTEX cohort for healthy liver tissues (n=110). Error bars represent SD. Data were analysed by one-way ANOVA with multiple comparisons (>2 groups). Significance is denoted as follows: **p<0.01 and ****p<0.0001.

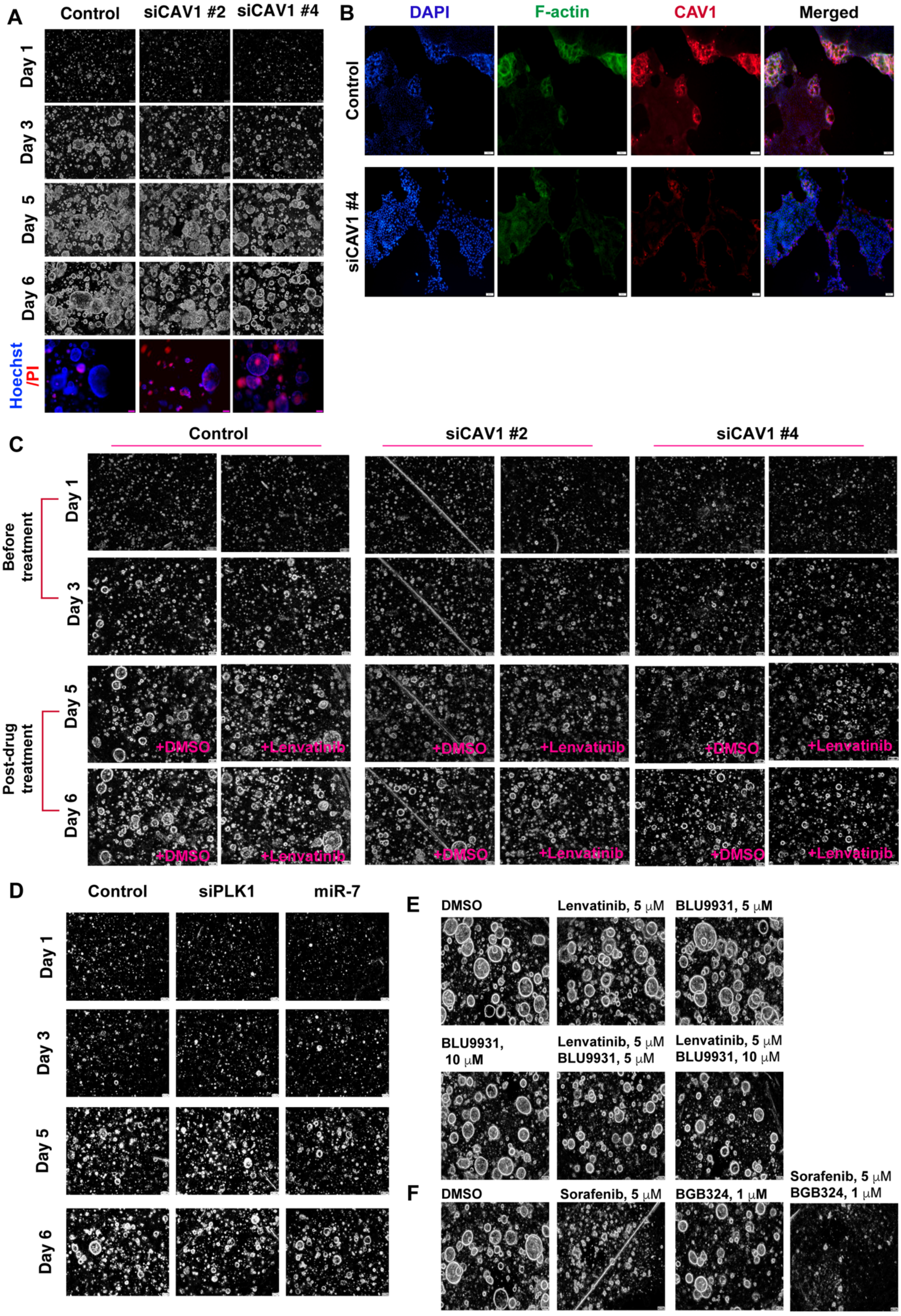

A
Control
siCAV1 #2
siCAV1 #4
Day 1
Day 3
Day 5
Day 6
Hoechst
/PI
B
DAPI
F-actin
CAV1
Merged
Control
siCAV1 #4
C
Control
siCAV1 #2
siCAV1 #4
Before treatment
Day 1
Day 3
Post-drug treatment
Day 5
Day 6
+DMSO
+Lenvatinib
D
Control
siPLK1
miR-7
Day 1
Day 3
Day 5
Day 6
E
DMSO
Lenvatinib, 5 μM
BLU9931, 5 μM
BLU9931, 10 μM
Lenvatinib, 5 μM BLU9931, 5 μM
Lenvatinib, 5 μM BLU9931, 10 μM
F
DMSO
Sorafenib, 5 μM
BGB324, 1 μM
Sorafenib, 5 μM BGB324, 1 μM

986

987

Suppl. Fig. S18: Image analysis of HCC PDOs following different drug treatments. **(A)** Effect of CAV1 knockdown in HCC PDO growth (n=3). **(B)** Immunofluorescence staining of CAV1 in HCC PDO derived cells in 2D culture (n=3). **(C)** Impact of siCAV1 and lenvatinib combination therapy on HCC PDO growth (n=3). **(D)** Effect of miR-7 overexpression on HCC PDO growth (n=3). **(E)** Synergy assessment between lenvatinib and BLU9931 compared to DMSO controls (n=3) and **(F)** Synergy assessment between sorafenib and BGB324 compared to DMSO controls. For siRNA experiments, non-targeting siRNA served as the lead control with siPLK1 monitoring transfection efficiency.

Genes	Description	Sorafenib		Lenvatinib		Cabozantinib	
		Rank	r	Rank	r	Rank	r
**FLT1** (VEGFR1)	fms related tyrosine kinase 1			9427	0.01	4579	0.07
**FLT4** (VEGFR3)	fms related tyrosine kinase 4	877	0.16	4127	0.08	9386	0.01
**FLT3**	fms related tyrosine kinase 3	3212	0.11			13010	-0.03
**KIT**	KIT proto-oncogene, receptor tyrosine kinase	6091	0.07	1551	0.13	6154	0.05
**BRAF**	B-Raf-proto-oncogne, serine-threonine kinase	9920	0.03				
**KDR** (VEGFR2)	Kinase insert domain receptor	13258	-0.02			8296	0.03
**PDGFRβ**	Platelet derived growth factor receptor beta	9433	0.03				
**PDGFRα**	Platelet derived growth factor receptor alpha			391	0.18		
**FGFR1**	Fibroblast growth factor 1			2447	0.11		
**FGFR2**	Fibroblast growth factor 2			11063	0		
**FGFR3**	Fibroblast growth factor 3			16683	-0.11		
**FGFR4**	Fibroblast growth factor 4			15423	-0.07		
**RET**	RET receptor tyrosine kinase			6117	0.05	5919	0.05
**NTRK2**	Neurotrophic receptor kinase 2					14024	-0.04
**AXL**	AXL receptor tyrosine kinase					15898	-0.07
**MET**	MET receptor tyrosine kinase					18435	-0.2

CTRPv2.0 and CCLE, n = 785 , "r" = correlation coefficient score

Table S2: Correlation coefficient score and ranking of validated TKI targets according to their sensitivity in 785 treatment-naïve human cancer cell lines

Genes	Description	Sorafenib		Lenvatinib		Cabozantinib	
		Rank	r	Rank	r	Rank	r
**MYOF**	Myoferlin	18509	-0.27	18469	-0.26	18168	-0.16
**CAV1**	Caveolin 1	18403	-0.22	17818	-0.17	17276	-0.11
**RAB27B**	Ras oncogene family RAB27B	18334	-0.21	18209	-0.21	15914	-0.07
**TGFB2**	Transforming growth factor beta 2	18192	-0.19	15281	-0.07	15481	-0.06
**SEMA3C**	Semaphorin 3C	18244	-0.19	17224	-0.13	18035	-0.15
**AXL**	AXL receptor tyrosine kinase	18000	-0.17	17111	-0.13	15898	-0.07
**SLPI**	Secretory leukocyte peptidase inhibitor	18069	-0.17	18361	-0.23	18239	-0.17
**COL12A1**	Collagen type XII alpha 1 chain	17745	-0.15	16124	-0.09	17010	-0.1
**BIRC3**	Baculoviral IAP repeat containing 3	17179	-0.12	16074	-0.09	16278	-0.08
**HK1**	Hexokinase 1	17128	-0.12	15661	-0.08	14895	-0.05
**CD47**	Leukocyte surface antigen CD47	16853	-0.11	12397	-0.02	12951	-0.02
**CDC42EP3**	CDC42 effector protein 3	17326	-0.13	16355	-0.1	15432	-0.06
**FLNC**	Filamin C	16310	-0.09	13060	-0.03	12925	-0.02
**GPRC5A**	G protein coupled receptor class C group 5 member A	18513	-0.28	18509	-0.3	18351	-0.18
**ANXA1**	Annexin A1	18194	-0.19	18208	-0.21	15905	-0.07
**FSTL1**	Follistatin like 1	17563	-0.14	11890	-0.01	16662	-0.09
**EXPH5**	Exophilin 5	18073	-0.17	18076	-0.19	18243	-0.17
**RBMS3**	RNA binding motif single stranded interacting protein 3	16036	-0.08	10959	0	14230	-0.04
**LUM**	Lumicane	14327	-0.04	6286	0.05	8370	0.03
**PAPPA**	Pappalysin-1or Pregnancy	13788	-0.03	7875	0.03	7398	0.04

	associated plasma protein A						
**MAP7D3**	MAP7 domain containing 3	8688	0.04	3356	0.09	4711	0.07
**NEBL**	Nebulin like protein	14626	-0.04	16393	-0.1	16109	-0.08
**EMP3**	Epithelial membrane protein 3	8550	0.04	5812	0.05	1724	0.12

CTRPv2.0 and CCLE, n = 785 , “r” = correlation coefficient score

Table S3: Ranking and correlation coefficient score of SR23 gene signature according to their sensitivity to sorafenib, lenvatinib and cabozantinib in 785 treatment-naïve human cancer cell lines

Variable	Rho or Chi2	*P*	n
Gender (male vs. female)	0.385	0.535	367
Age (years)	-1.77	0.077	366
Viral hepatitis (yes vs. no)	7.84	0.005	275
HBV (yes vs. no)	5.43	0.020	163
HCV (yes vs. no)	2.12	0.145	163
HCC grade	4.73	0.173	362
T stage (1, 2, 3, 4)	7.12	0.068	364
N stage (positive vs. negative)	1.5	0.220	252
M stage (positive vs. negative)	1.53	0.216	267
Stage overall (0-4)	7.37	0.118	367
Alpha Fetoprotein	0.471	0.638	276
New tumor (any vs. no)	1.54	0.215	318
New tumor (recurrence vs. no)	1.58	0.209	317
History of alcohol (yes vs. no)	0.653	0.419	367
History of hemochromatosis (yes vs. no)	0.299	0.584	367
Tumor weight	-4.71	<0.0001	367
Tumor nuclei (%)	2.3	0.022	367
Tumour necrosis (%)	0.483	0.629	367

Table S4: Univariate association between CAV1 Youden Index and potential covariates In TCGA HCC cohort

Name of repository	Identifier	Link
Total RNA sequencing of sorafenib-resistant Huh-7 cells	**GSE20098**	**https://aus01.safelinks.protection.outlook.com/?url=https%3A%2F%2Fwww.ncbi.nlm.nih.gov%2Fgeo%2Fquery%2Facc.cgi%3Facc%3DGSE200098&data=04%7C01%7Ctasnuva.kabir%40perkins.uwa.edu.au%7Cc249f1493ec54e228db508da16b7ec9f%7C05894af0cb2846d8871674cdb46e2226%7C0%7C0%7C637847276447081288%7CUnknown%7CTWFpbGZsb3d8eyJWIjoiMC4wLjAwMDAiLCJQIjoiV2luMzIiLCJBTiI6Ik1haWwiLCJXVCI6Mn0%3D%7C3000&sdata=BF2PnNPYYOMUIXQI22CpN47TFbsEI1PE%2BfHn%2BF81uV8%3D&reserved=0**
scRNA sequencing of HCC patients	PRJNA658541	**https://www.ncbi.nlm.nih.gov/geo/query/acc.cgi?acc=GSE156625**

Table S5: Deposited data

Name	Sequence	Supplier
**Hs_CAV1 v1 Forward**	5’ ACGTAGACTCGGAGGGACAT 3’	Sigma Aldrich
**Hs_CAV1 v1 Reverse**	5’ TCTGCCATGGCCTTGTTGTT 3’	Sigma Aldrich
**Hs_AXL**	QT00067725	Qiagen
**Hs_TYRO3**	QT00055482	Qiagen
**Hs_EGFR**	QT00085701	Qiagen
**Hs_Albumin**	Hs00609411_m1	Thermo Fisher Scientific
**Hs_GAPDH**	4333764F	Thermo Fisher Scientific
**Hs_VIM**	QT00095795	Qiagen
**Hs_MMP9 Forward**	5’ ACTACGACACCGACGACCGGTT 3’	Sigma Aldrich
**Hs_MMP9 Reverse**	5’ GTGGTGCAGGCGGAGTAGGATT 3’	Sigma Aldrich
**Hs_α–SMA Forward**	5’ AGCACTGTCAGGAATCCTGTGAA 3’	Sigma Aldrich
**Hs_α-SMA Reverse**	5’ CATTGTCACACACCAAGGCAGT 3’	Sigma Aldrich
**Hs_ITGβ8**	Hs_00174456	Thermo Fisher Scientific
**Hs_IGF1R**	QT00005831	Qiagen
**Hs_E-cadherin**	QT00080143	Qiagen
**Hs_N-cadherin**	QT00063196	Qiagen
**Hs_POSTN**	QT00023800	Qiagen
**Hs_IL8**	QT00000322	Qiagen
**Hs_HPRT**	QT00059066	Qiagen
**Hs_Slug Forward**	5’ ATACAGCCCCATCACTGTGT 3’	Sigma Aldrich
**Hs_Slug Reverse**	5’ GACTCACTCGCCCCAAAGATGA 3’	Sigma Aldrich
**Hs_Snail Forward**	5’ CTAATCCAGAGTTTACCTTCC 3’	Sigma Aldrich
**Hs_Snail Reverse**	5’ ACAGAGTCCCAGATGAGCATTGG 3’	Sigma Aldrich
**Hs_Twist1 Forward**	5’ AAGATGGCAAGCTGCAGCTATGT 3’	Sigma Aldrich
**Hs_Twist1 Reverse**	5’ CTTCTCTGGAAACAATGACATCT 3’	Sigma Aldrich

**Hs_COL1A1 Forward**	5’ AAGGGTAACAGCGGTGAACCT 3’	Sigma Aldrich
**Hs_COL1A1 Reverse**	5’ TCGAGCTCCTCGCTTTCCTT 3’	Sigma Aldrich
**Hs_FGFR4**	Hs_01106910_g1	Thermo Fisher Scientific
**Hs_KLF4 Forward**	5’ TCCATTACCAAGAGCTCATGCCA 3’	Sigma Aldrich
**Hs_KLF4 Reverse**	5’ TTGAGATGGGAACTCTTTGTGTA 3’	Sigma Aldrich
**Hs_EpCAM Forward**	5’ AGTGTACTTCAGTTGGTGCACA 3’	Sigma Aldrich
**Hs_EpCAM Reverse**	5’ TAAAGCCCATCATTGTTCTGGAG 3’	Sigma Aldrich
**Hs_CD44 Forward**	5’ TTCAATGCTTCAGCTCCAC 3’	Sigma Aldrich
**Hs_CD44 Reverse**	5’ CCATCACGGTTAACAATAGT 3’	Sigma Aldrich
**Hs_CD133 Forward**	5’ TCTGAACAGTATCAATTCAGTG 3’	Sigma Aldrich
**Hs_CD133 Reverse**	5’ TCTTTGGTCTCCTTGATCGCTGTT 3’	Sigma Aldrich
**Hs_CD24 Forward**	5’ ACGCAGATTTATTCCAGTGAAACA 3’	Sigma Aldrich
**Hs_CD24 Reverse**	5’ GAAGAGACTGGCTGTTGACT 3’	Sigma Aldrich
**Hs_Myc**	QT00035406	Qiagen
**Hs_Nanog**	QT01025850	Qiagen
**Hs_Sox2_Forward**	5’ AAACCAAGACGCTCATGAAGAAG 3’	Sigma Aldrich
**Hs_Sox2_Reverse**	5’ GTTCATGTGCGCGTAACTGTCCAT 3’	Sigma Aldrich
**Hs_ITGβ3**	QT00044590	Qiagen
**Hs_CDK2_Forward**	5’ TGGACACTGAGACTGAGGGTGTG 3’	Sigma Aldrich
**Hs_CDK2_Reverse**	5’ GACATCCAGCAGCTTGACAATATT 3’	Sigma Aldrich
**Hs_CDKN2C_Forward**	5’ TGCAGGTTATGAAACTTGGAAAT 3’	Sigma Aldrich
**Hs_CDKN2C_Reverse**	5’ TCGATGTTAACATCAGCTTGAAA 3’	Sigma Aldrich
**Hs_CDK1**	QT00042672	Qiagen
**Hs_BCL2**	QT00025011	Qiagen
**Hs_ENO2_Forward**	5’ AGCCTCTACGGGCATCTATGA 3’	Sigma Aldrich
**Hs_ENO2_Reverse**	5’ TTCTCAGTCCCATCCAACTCC 3’	Sigma Aldrich

Table S6: Sequences and IDs of Primers
