# Supplementary material for: Inhibition of the Caveolin-1 pathway promotes apoptosis and overcomes pan-tyrosine kinase inhibitor resistance in hepatocellular carcinoma
Source: Cell Death Dis. 2025 Jul 25;16(1):561. doi: 10.1038/s41419-025-07887-4 (PMC12297225; doi:10.1038/s41419-025-07887-4)

Suppl. Fig. 2:

Suppl. Fig. S2F:

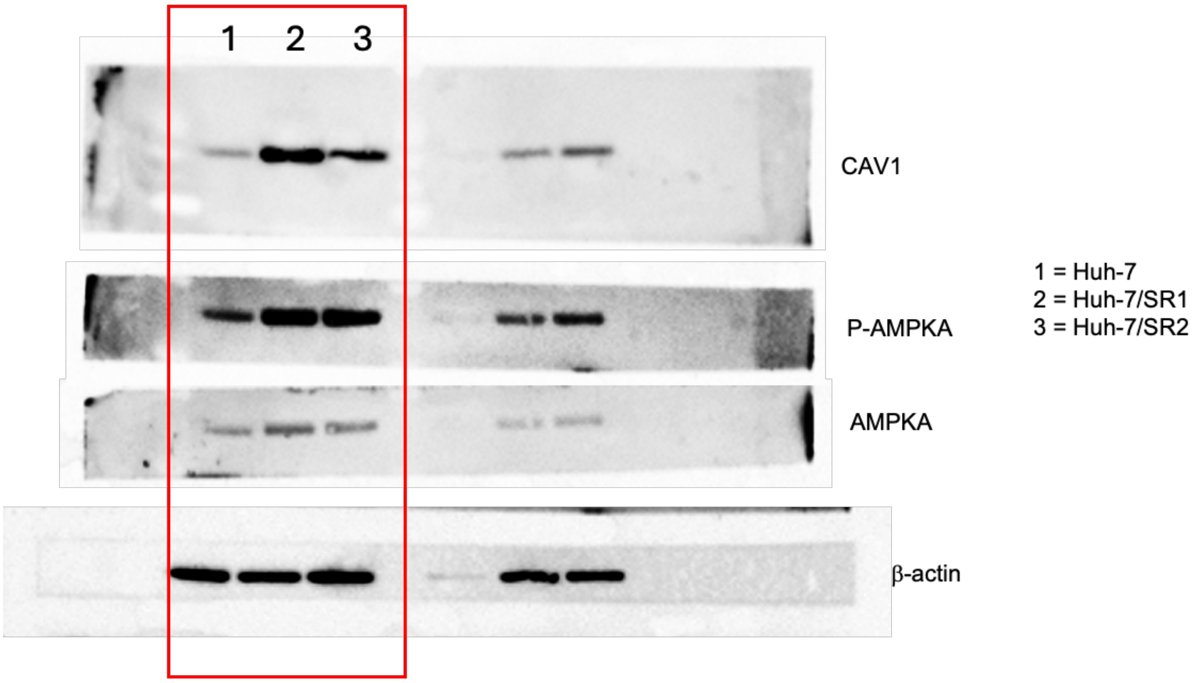

Suppl. Fig. S2H:

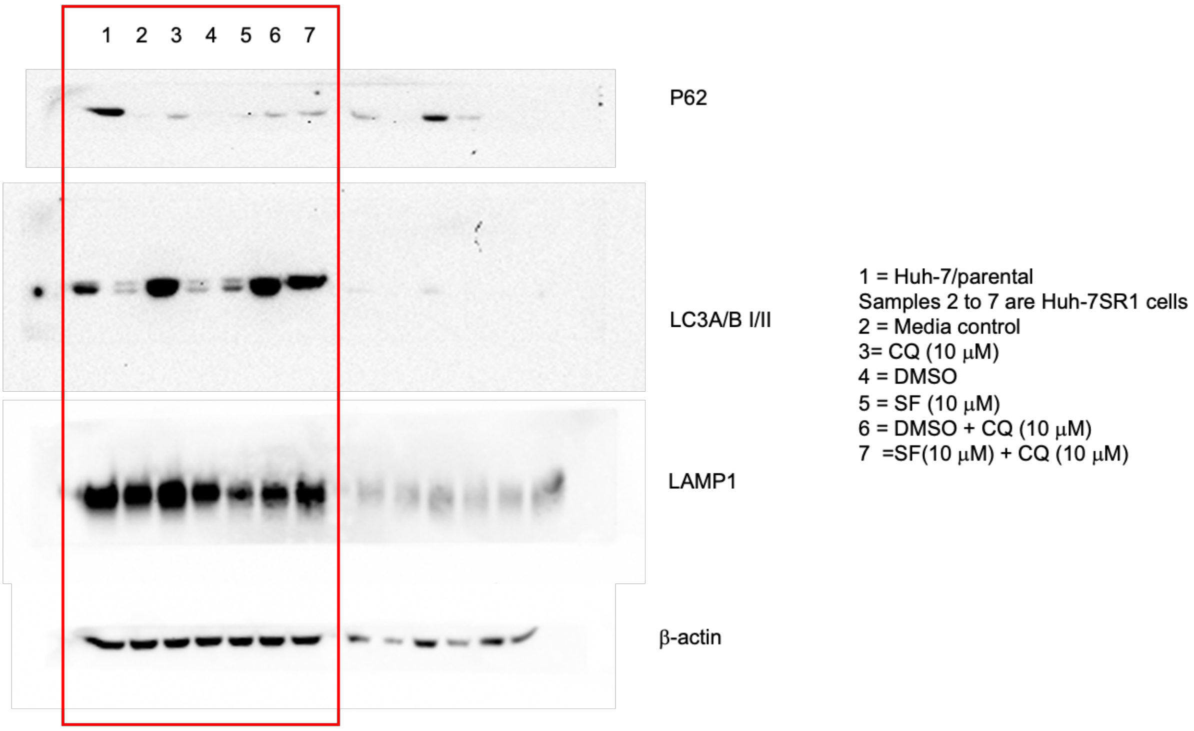

Suppl. Fig. S2I:

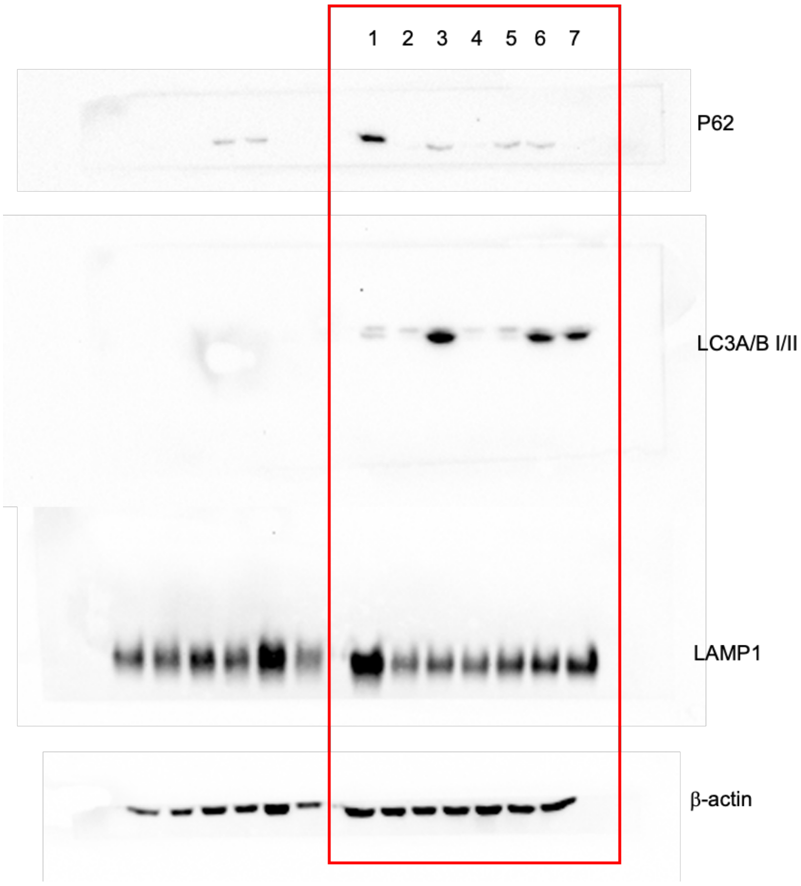

1 = Huh-7/parental
Samples 2 to 7 are Huh-7LR cells
2 = Media control
3= CQ (10 μM)
4 = DMSO
5 = Lenvatinib (10 μM)
6 = DMSO + CQ (10 μM)
7 = Lenvatinib (10 μM) + CQ (10 μM)

Fig. 2:

Fig. 2C:

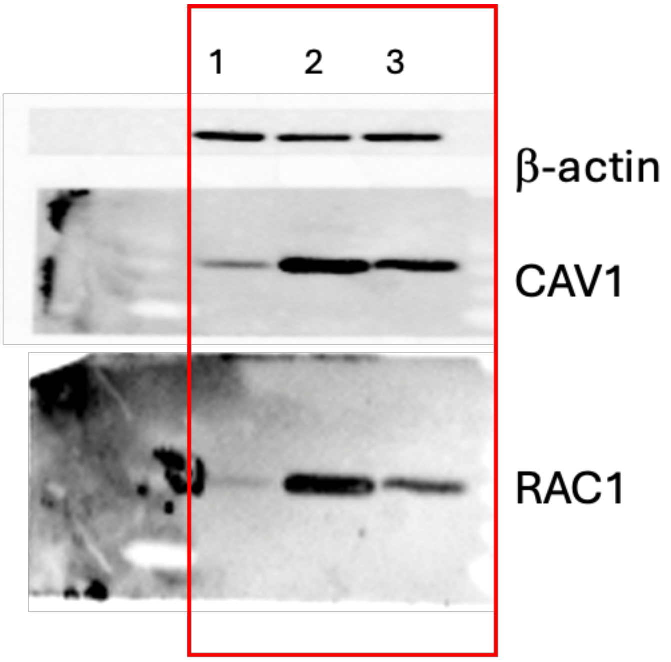

1 Huh-7
2 Huh-7/SR1
3 Huh-7/SR2

Fig. 2F:

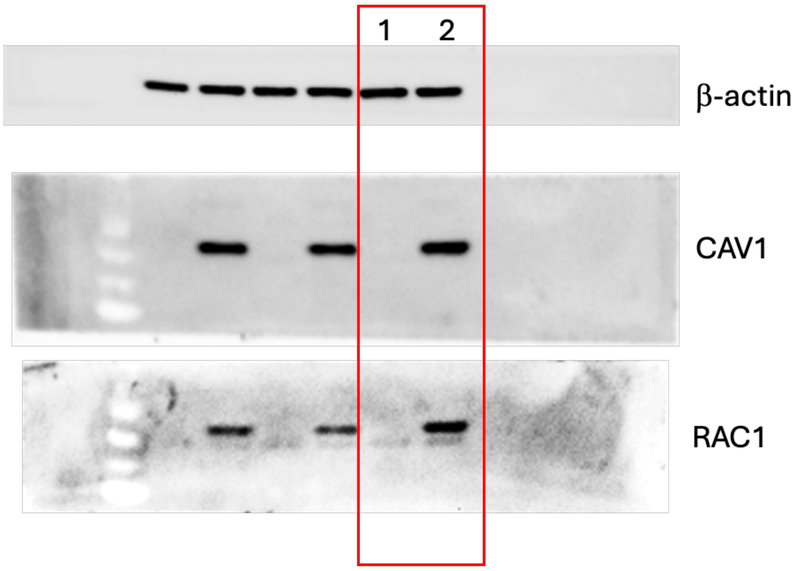

1 Huh-7-EV
2 Huh-7-CAV1-OE

Fig. 2I:

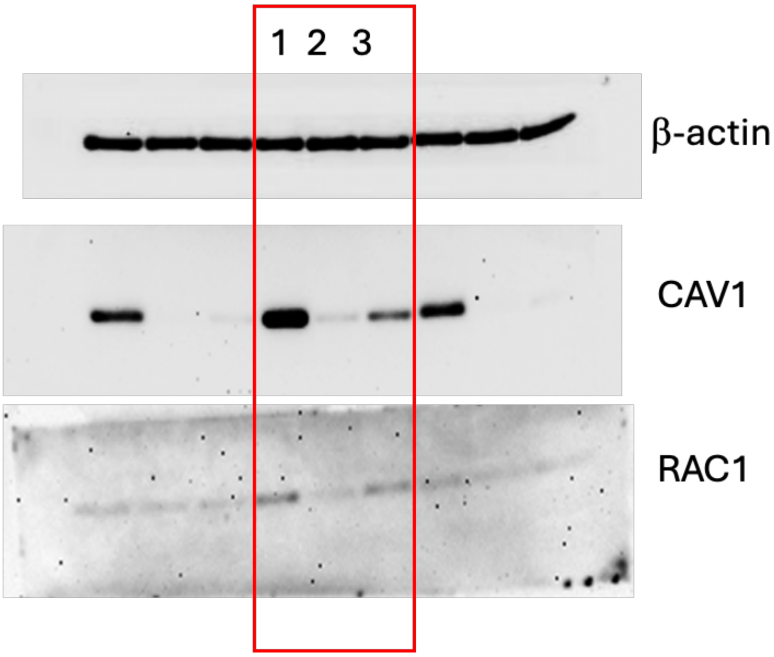

1 siNC_Huh-7SR1
2 siCAV1 #2_Huh-7SR1
3 siCAV1 #4_Huh-7SR1

Fig. 2O:

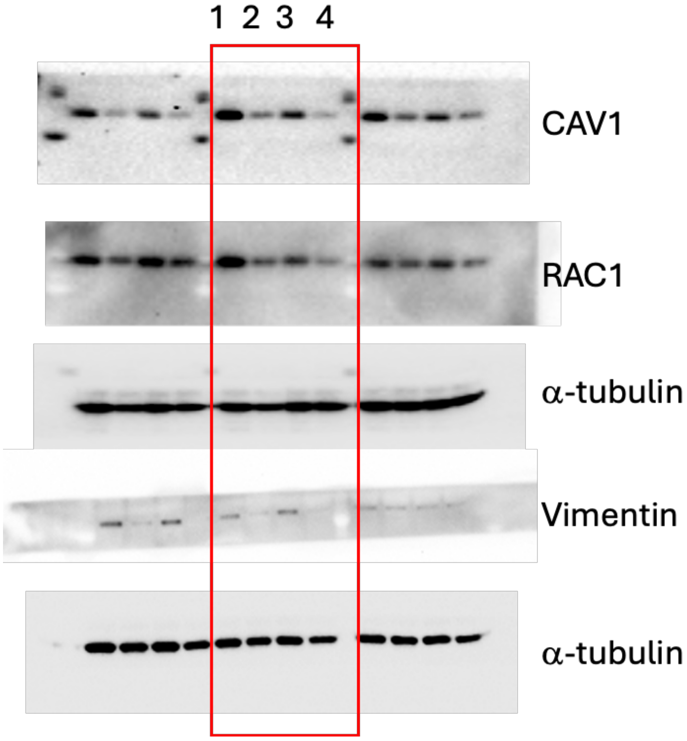

Huh-7/SR1 cells:

1 siNC + DMSO
2 siCAV1 #2 + DMSO
3 siNC + SF
4 siCAV1 #2 + SF

Fig. 2R:

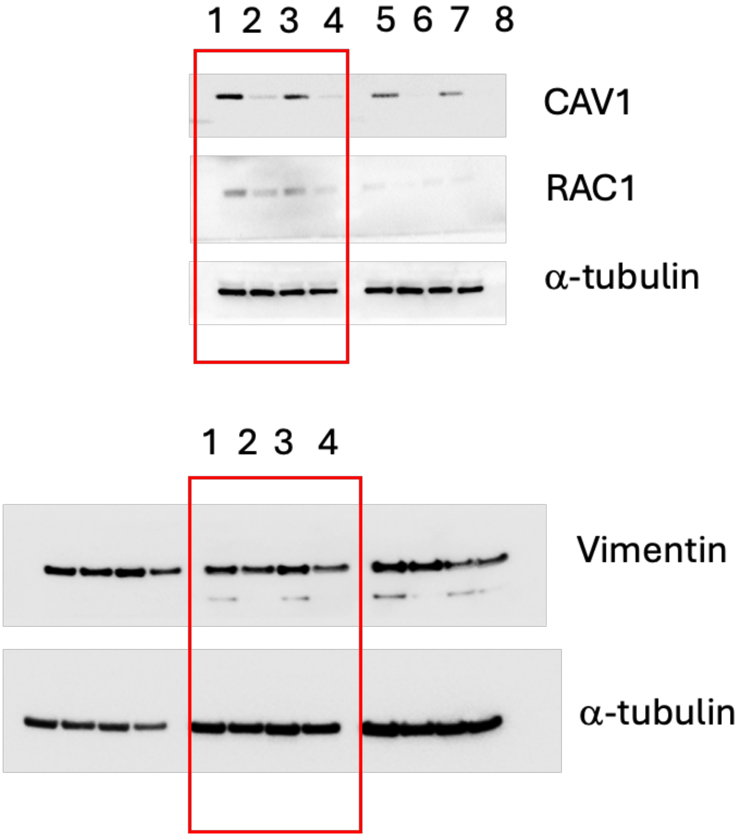

Huh-7/LR cells:

1 siNC + DMSO
2 siCAV1 #2 + DMSO
3 siNC + SF
4 siCAV1 #2 + SF

Suppl. Fig. S3

Suppl. Fig. S3C

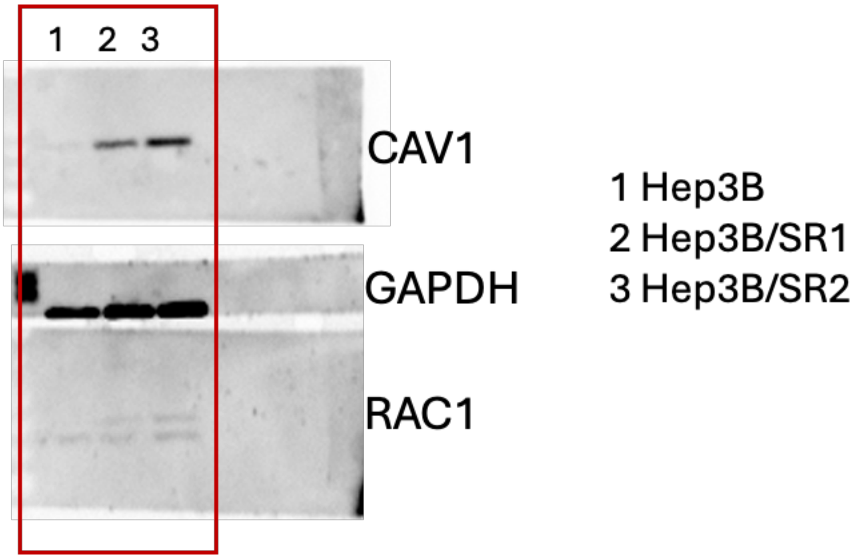

Suppl. Fig. S3E

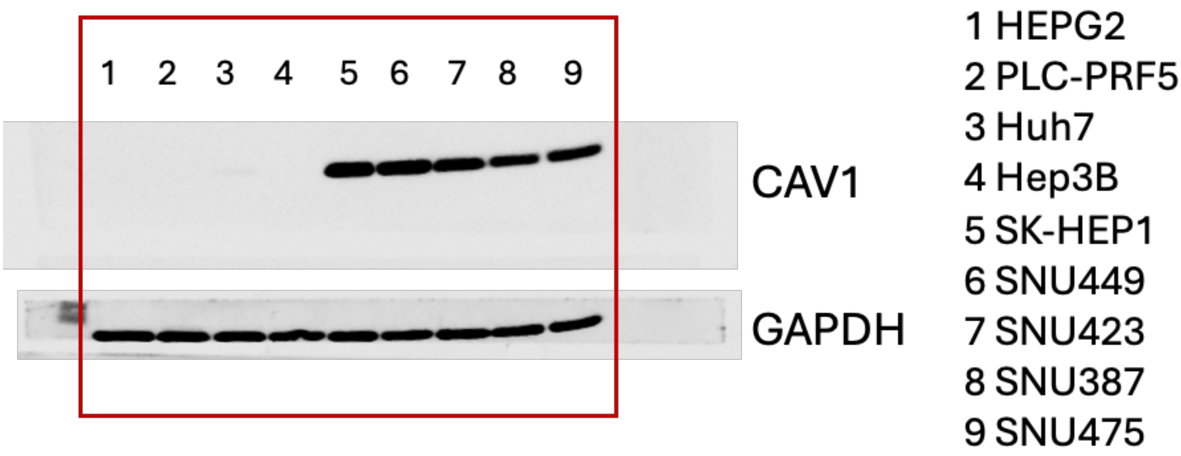

Suppl. Fig. S4:

Suppl. Fig. S4B:

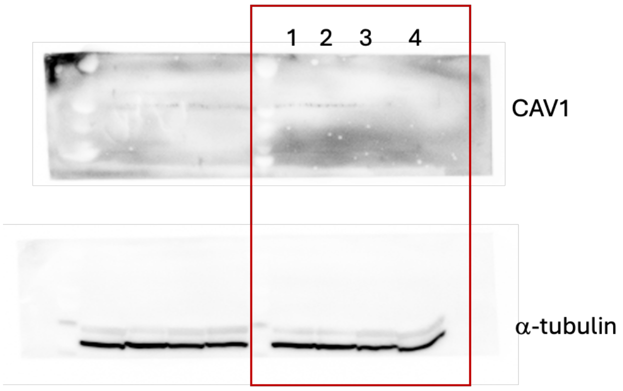

Huh-7/SR1 cells:

1 = siNC_Day 4
2 = siNC Day 7
3 = siCAV1#2 Day 4
4 = siCAV1#2 Day 7

Suppl. Fig. S4H:

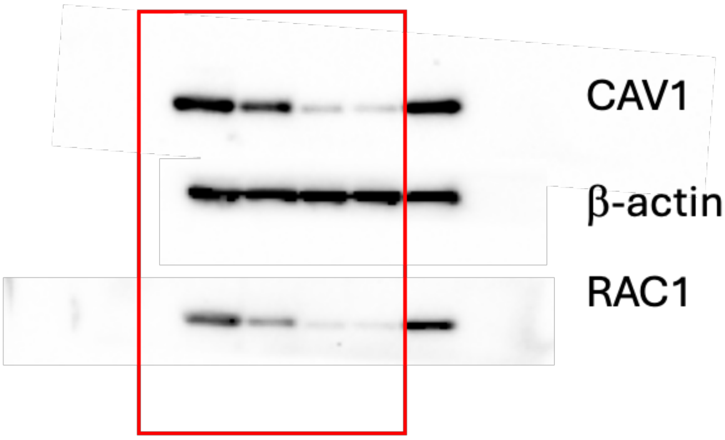

SNU475

1 = shCONtrol
2 = shCAV1 7999
3 = shCAV1 8000
4 = shCAV18002
5 = shAXL193

Suppl. Fig S4J:

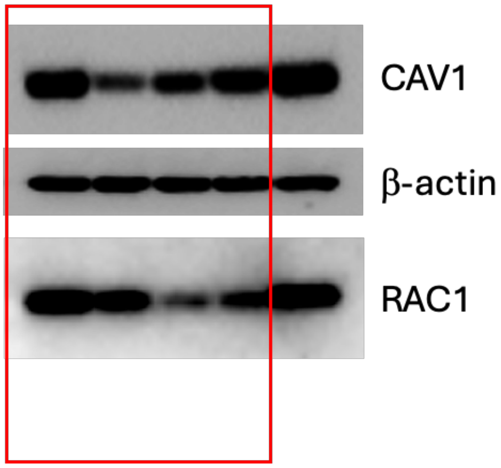

SNU449

1 = shCONtrol
2 = shCAV1 7999
3 = shCAV1 8000
4 = shCAV18002
5 = shAXL193

Fig. 4:

Fig. 4A:

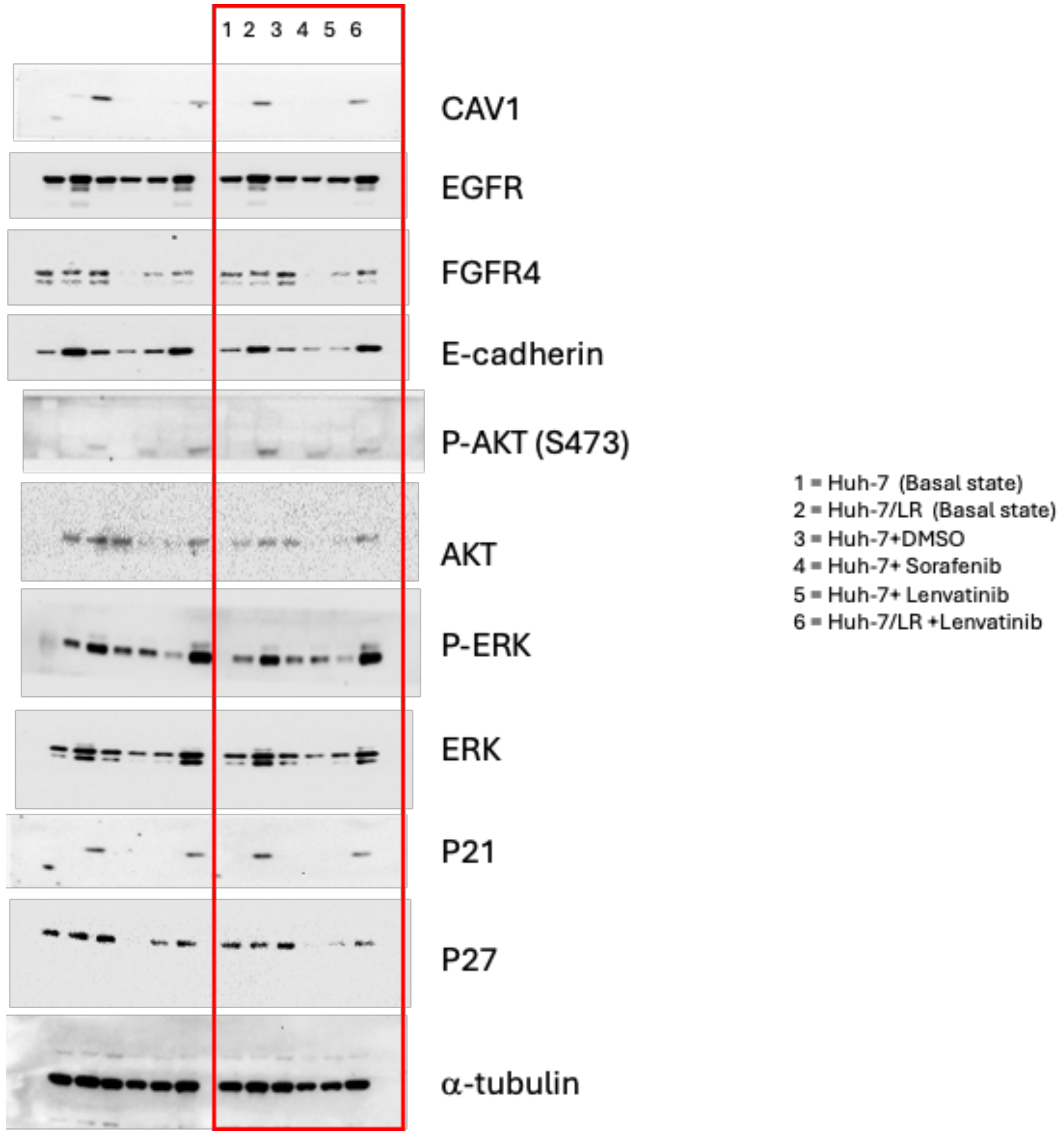
1 2 3 4 5 6
CAV1
EGFR
FGFR4
E-cadherin
P-AKT (S473)
AKT
P-ERK
ERK
P21
P27
α-tubulin
1 = Huh-7 (Basal state)
2 = Huh-7/LR (Basal state)
3 = Huh-7+DMSO
4 = Huh-7+ Sorafenib
5 = Huh-7+ Lenvatinib
6 = Huh-7/LR +Lenvatinib

Fig. 4C:

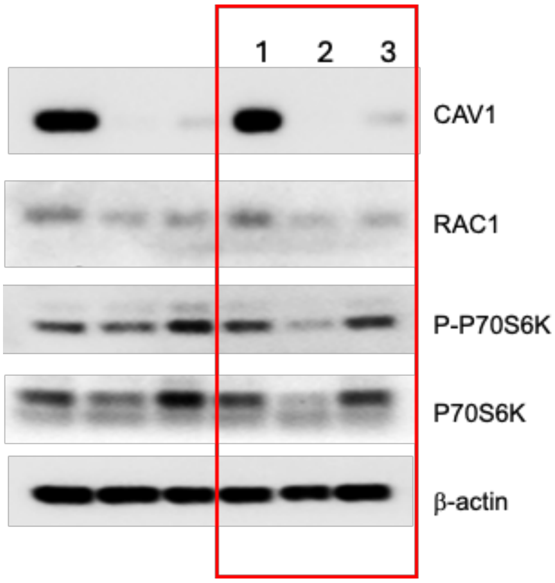

1 : Huh-7/SR1 siNC, 30 nM
2: Huh-7/SR1 siCAV1, #2, 30 nM
3: Huh-7/SR1 siCAV1, #4, 30 nM

Fig 4D:

Fig. 4D: Left Panel

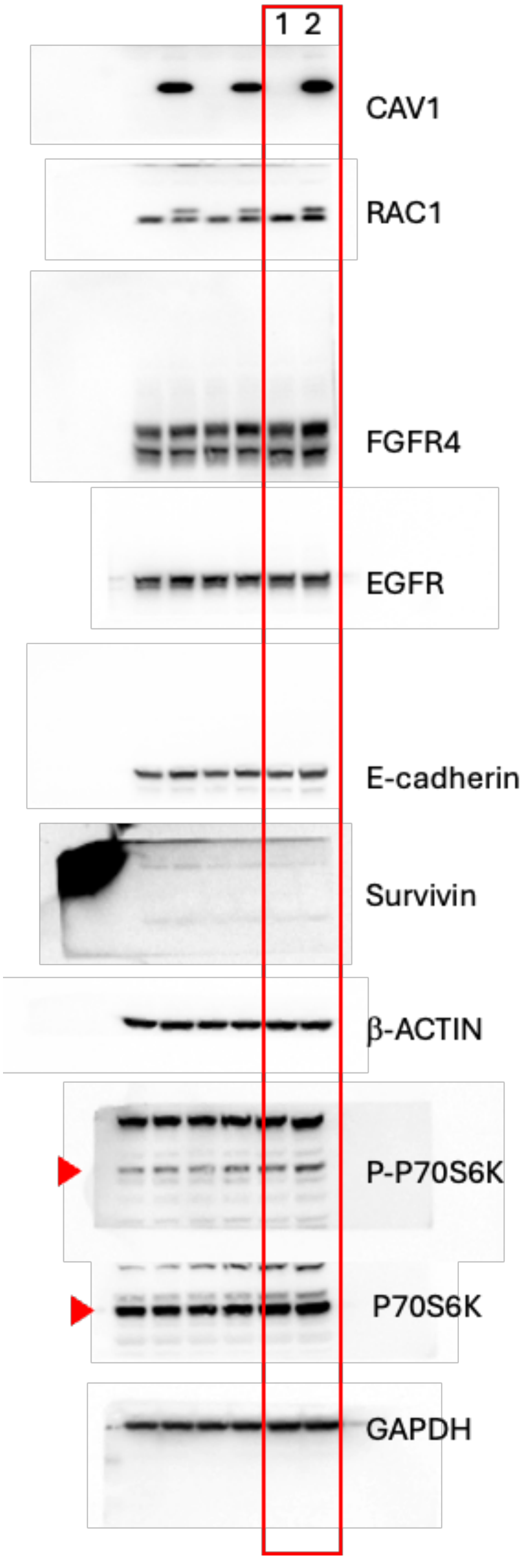

1 Huh-7 empty vector (EV)
2 Huh-7 CAV1 over expression (OE)

Fig 4D: Right Panel

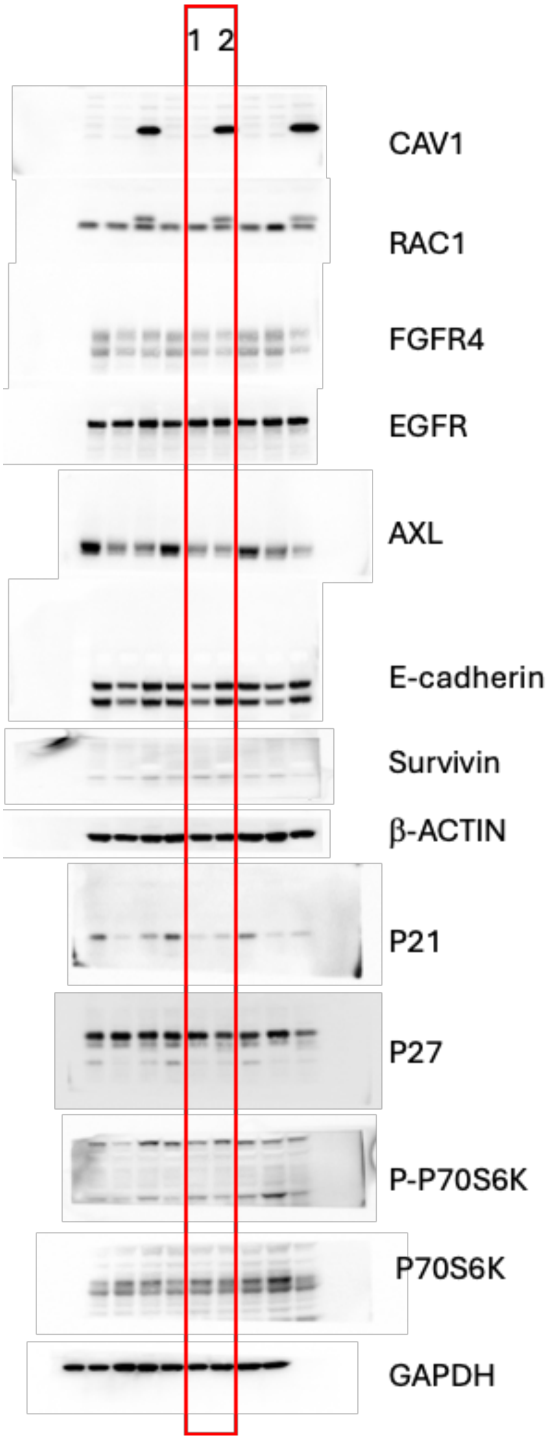

1 PLC-PRF-5 empty vector (EV)
2 PLC-PRF-5 CAV1 over expression (OE)

Fig 4F:

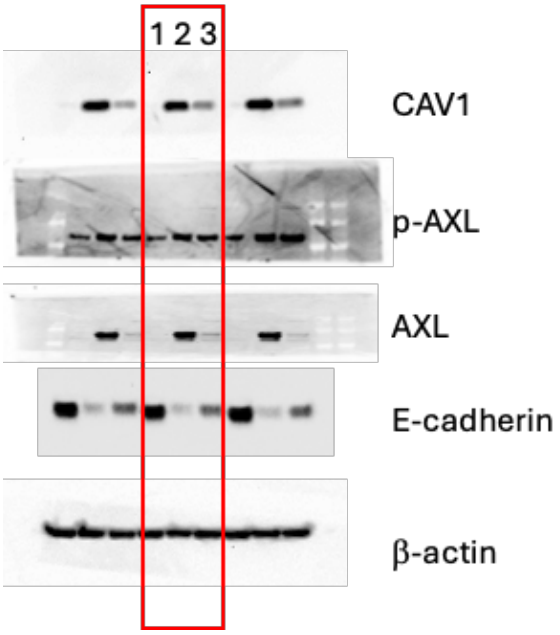

1 = Huh-7
2 = Huh-7/SR1
3 = Huh-7/SR2

Fig 4G:

Fig 4G: Left panel

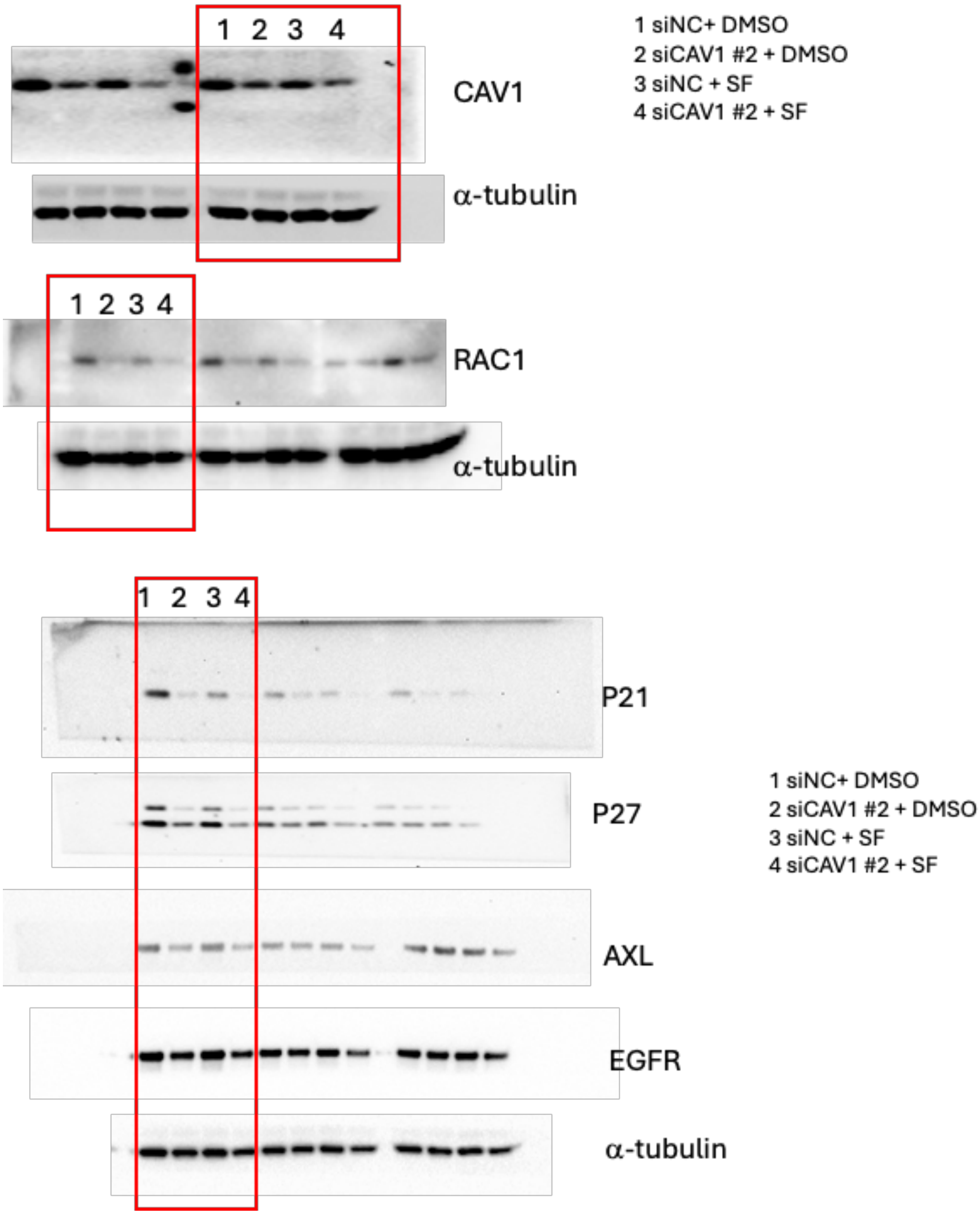

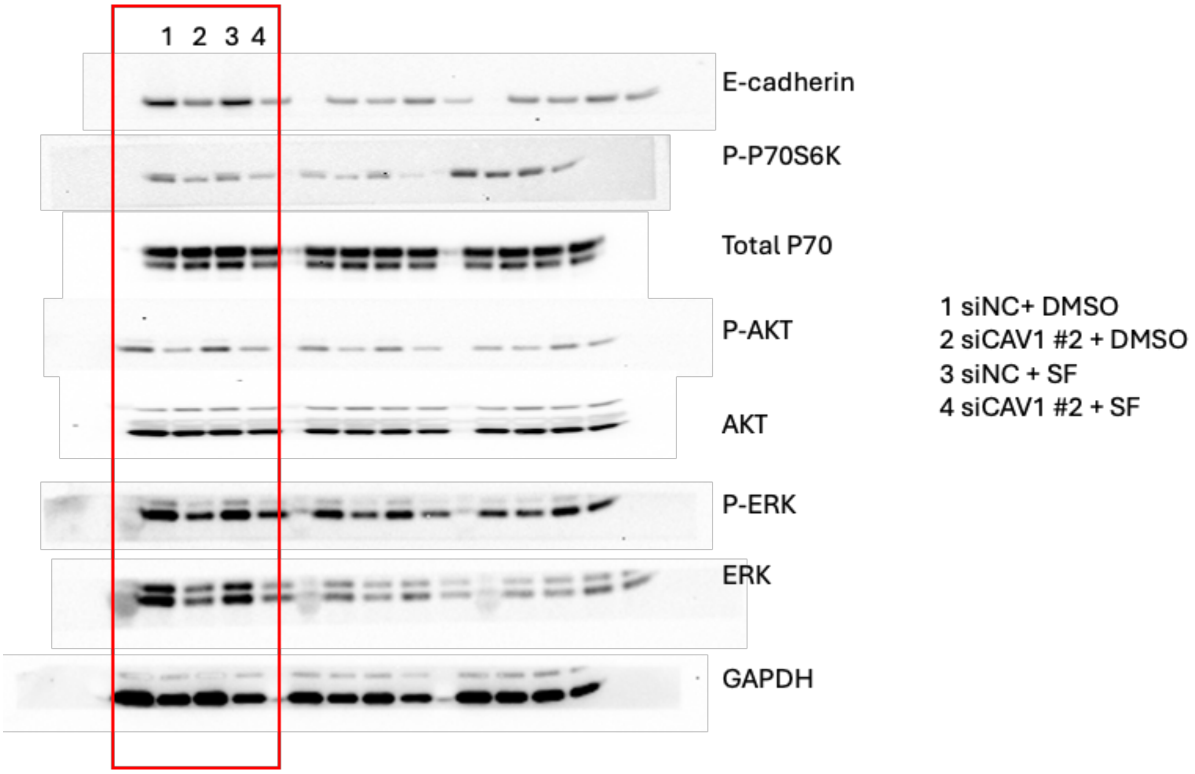

Fig 4G: Right Panel

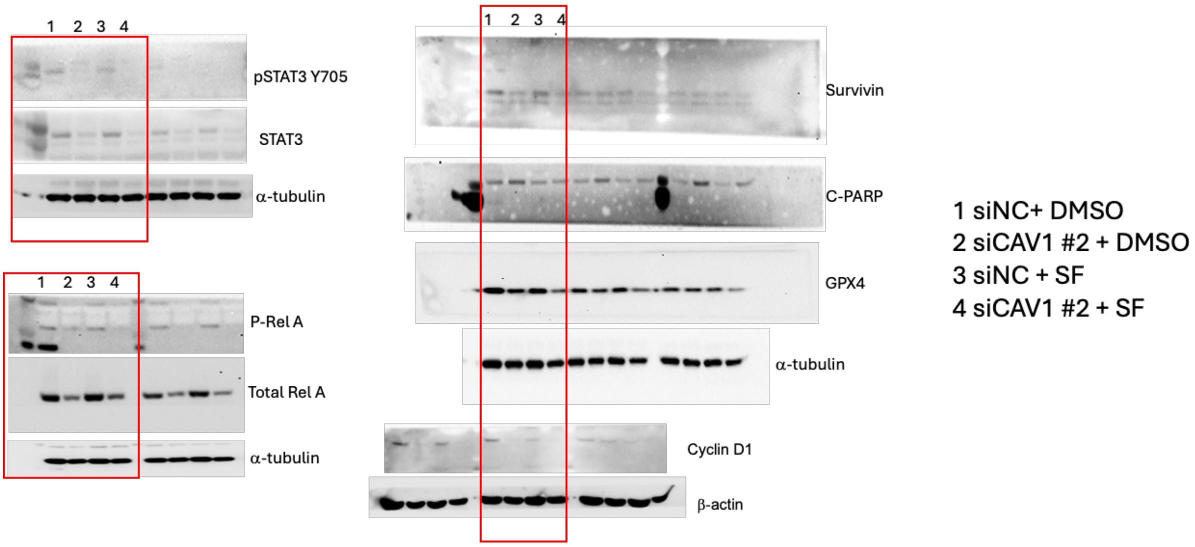

Fig 4H:

Fig 4H: Left Panel

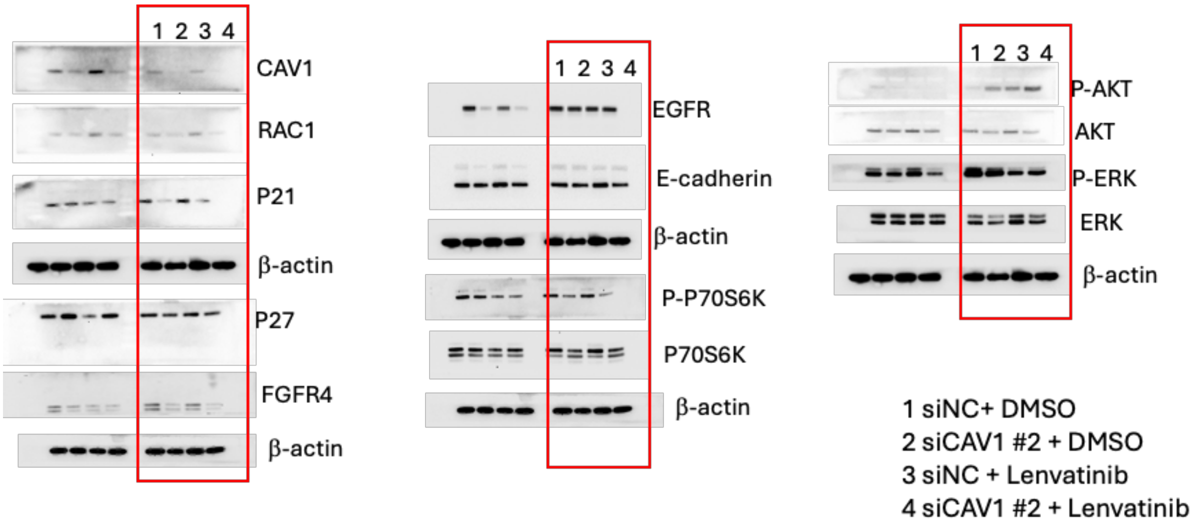

Fig 4H: Right Panel

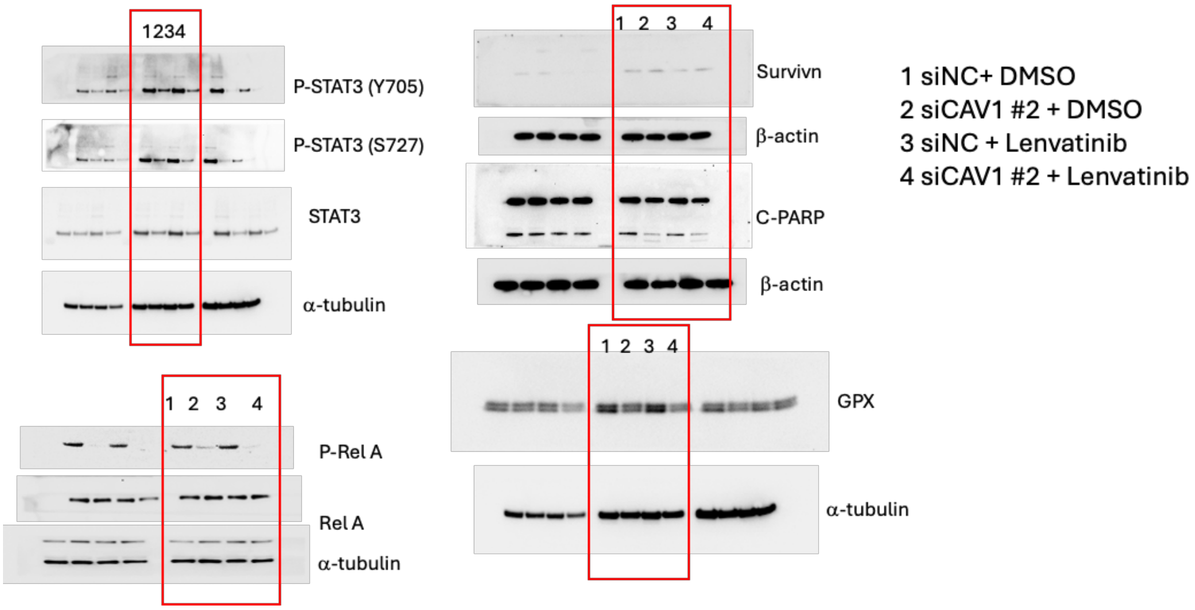

Fig 4K:

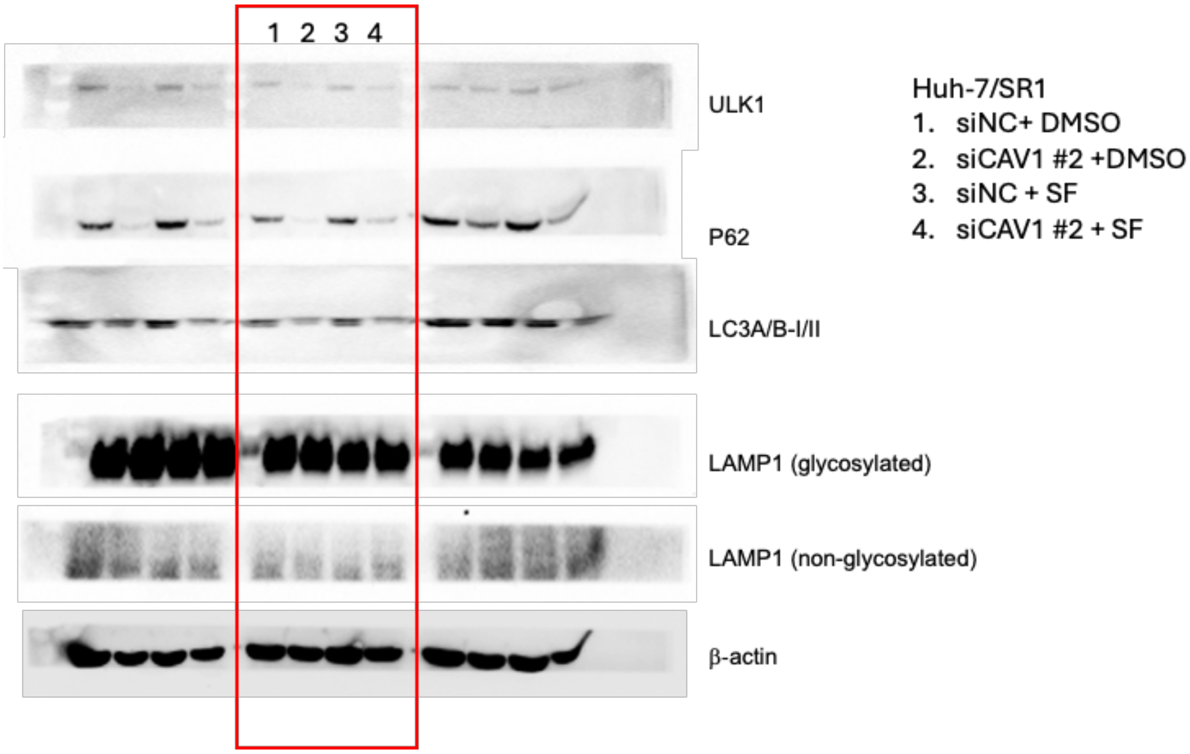
1 2 3 4
ULK1
P62
LC3A/B-I/II
LAMP1 (glycosylated)
LAMP1 (non-glycosylated)
β-actin
Huh-7/SR1
1. siNC+ DMSO
2. siCAV1 #2 +DMSO
3. siNC + SF
4. siCAV1 #2 + SF

Suppl. Fig. S6:

Suppl. Fig, S6A:

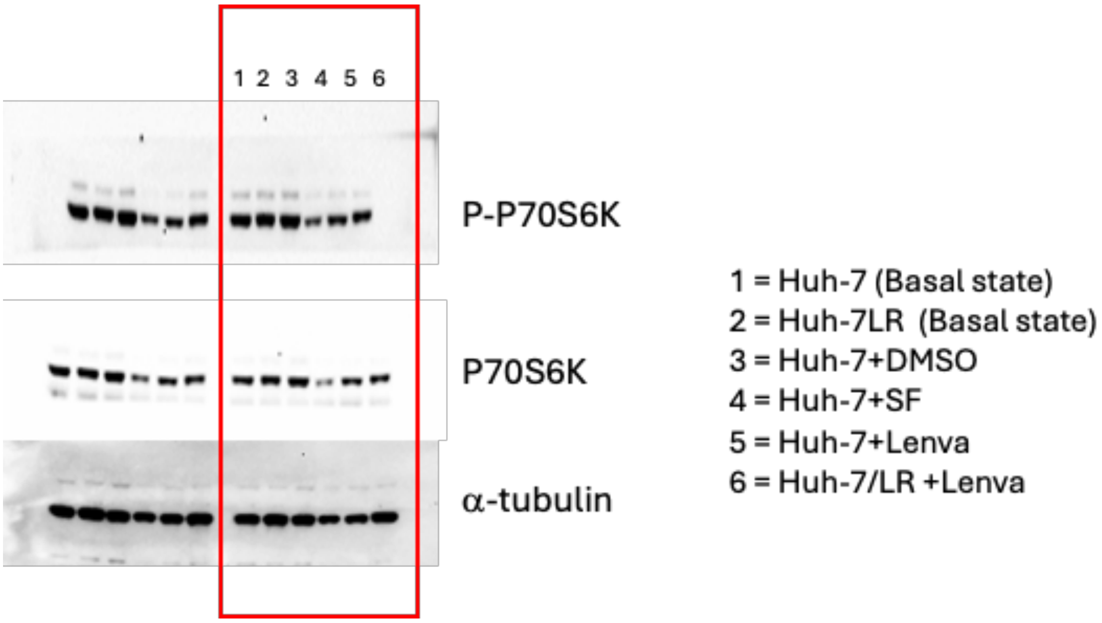

Suppl. Fig, S6B:

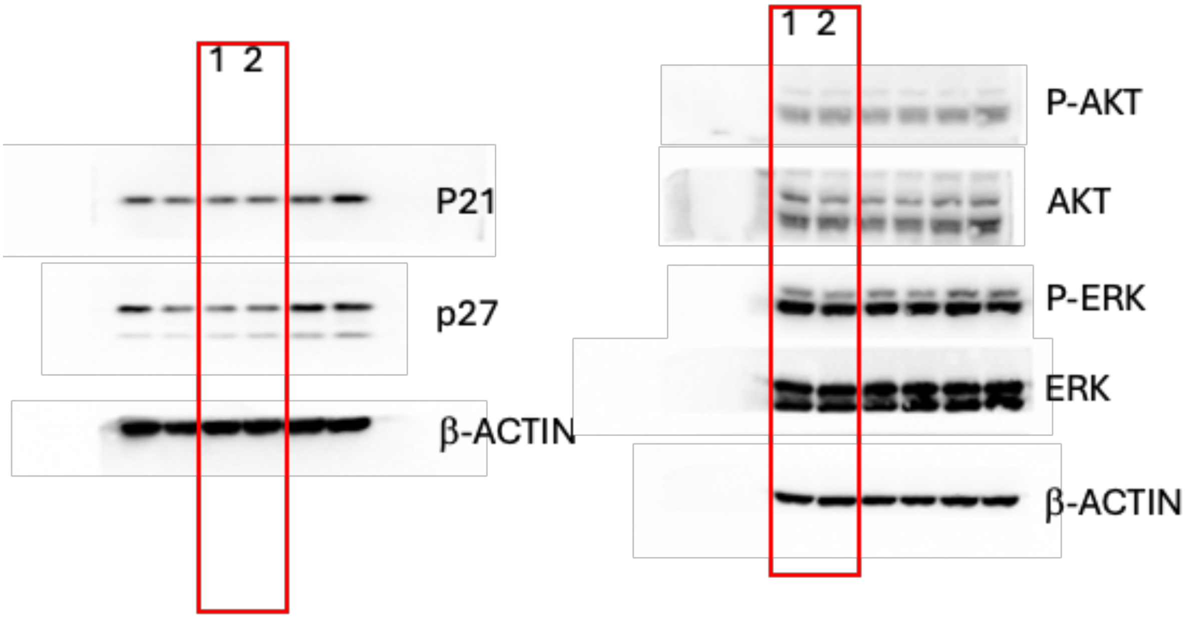

1 Huh-7 empty vector (EV)
2 Huh-7 CAV1 over expression (OE)

Suppl. Fig, S6D:

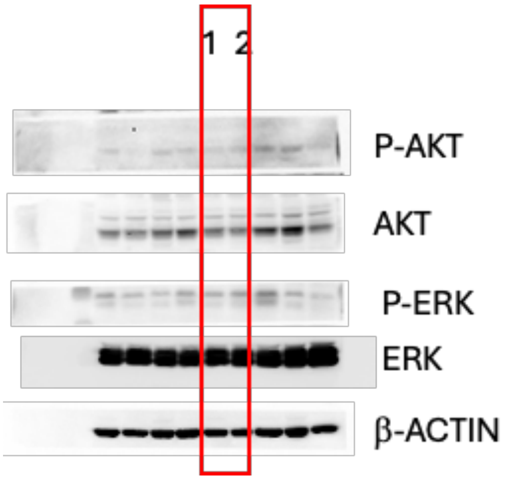

1 PLC-PRF-5 empty vector (EV)
2 PLC-PRF-5 CAV1 over expression (OE)

Suppl. Fig. S6 F:

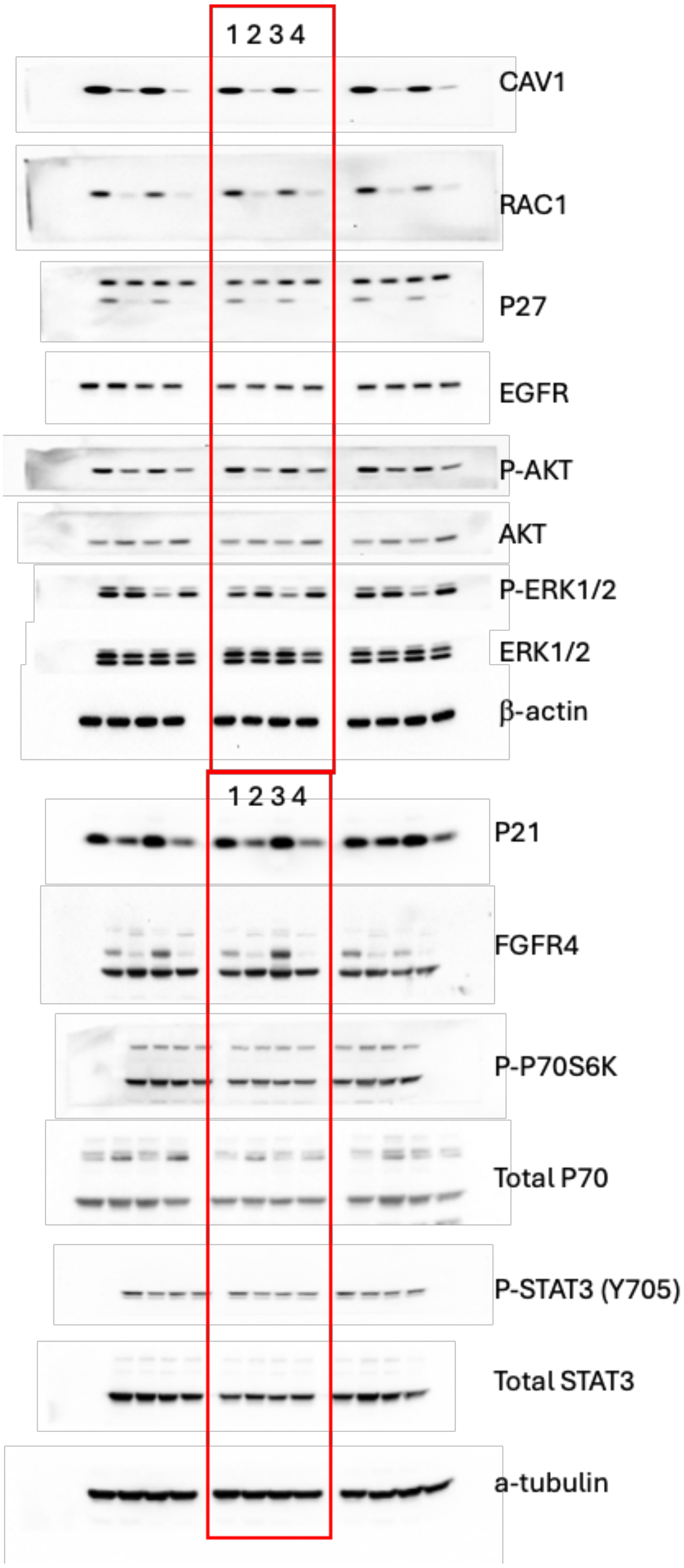

SNU475 cells:

1 : shcontrol + DMSO

2 : shCAV1 #2 + DMSO

3 : shcontrol + Lenva

4 : shCAV1 #2 + Lenva

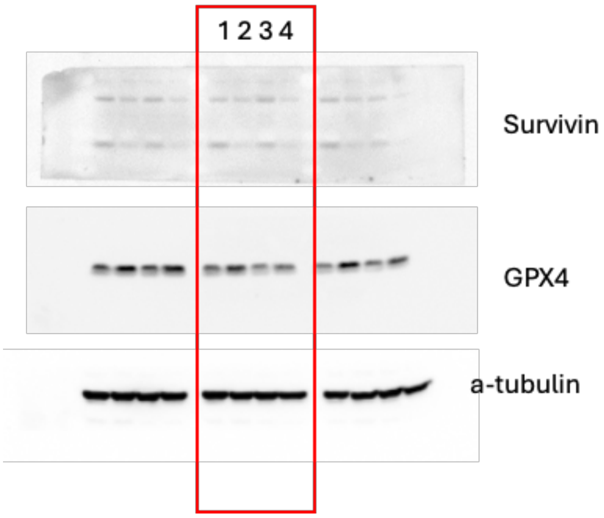

SNU475 cells:
1 : shcontrol + DMSO
2 : shCAV1 #2 + DMSO
3 : shcontrol + Lenva
4 : shCAV1 #2 + Lenva

Suppl. Fig, S6 G:

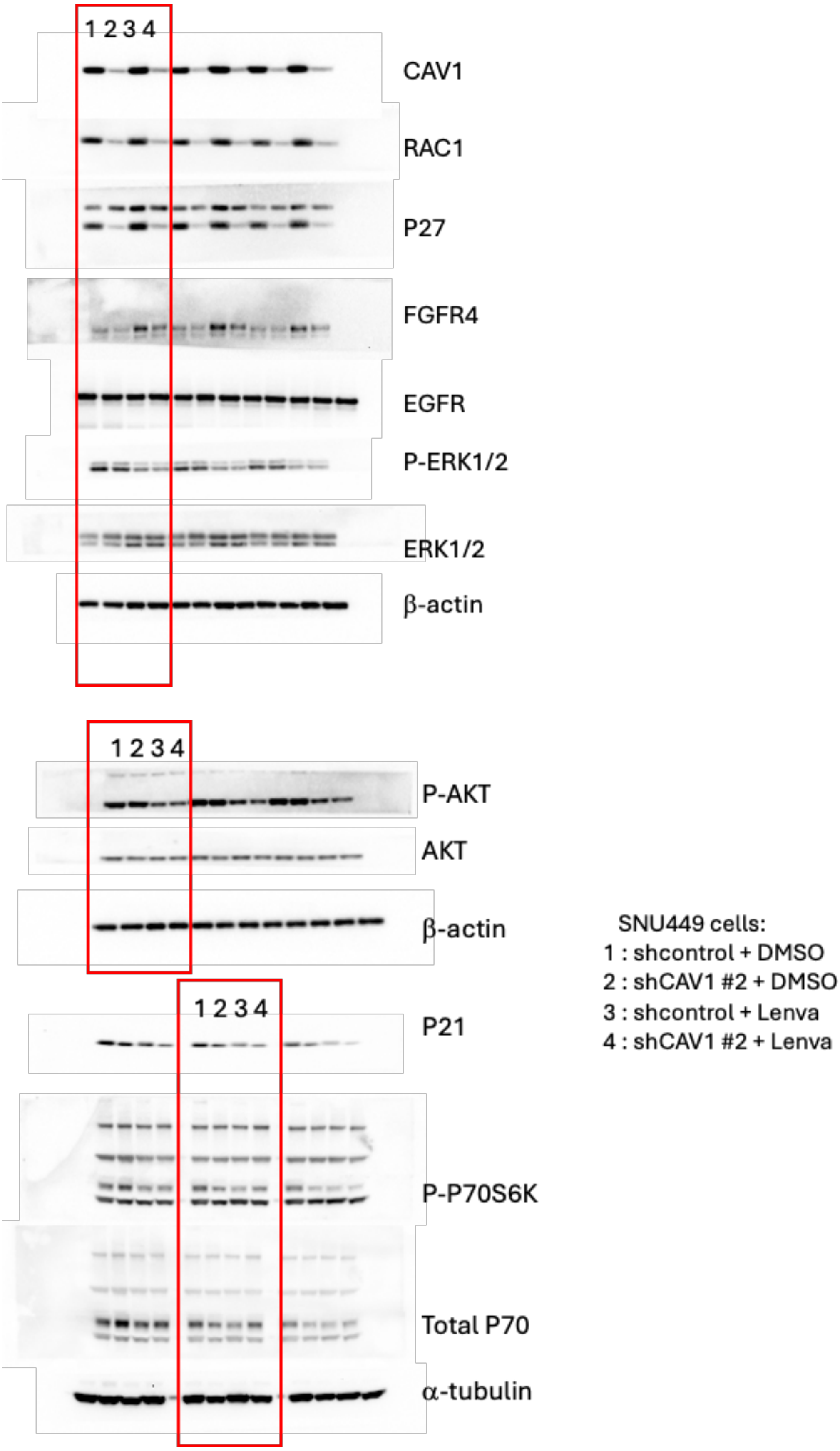

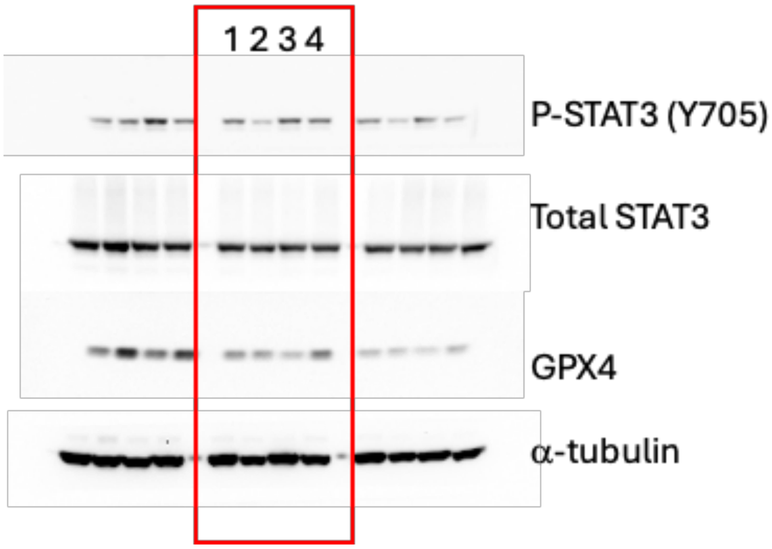

SNU449 cells:
1 : shcontrol + DMSO
2 : shCAV1 #2 + DMSO
3 : shcontrol + Lenva
4 : shCAV1 #2 + Lenva

Suppl. Fig. S7:

Suppl. Fig. S7A:

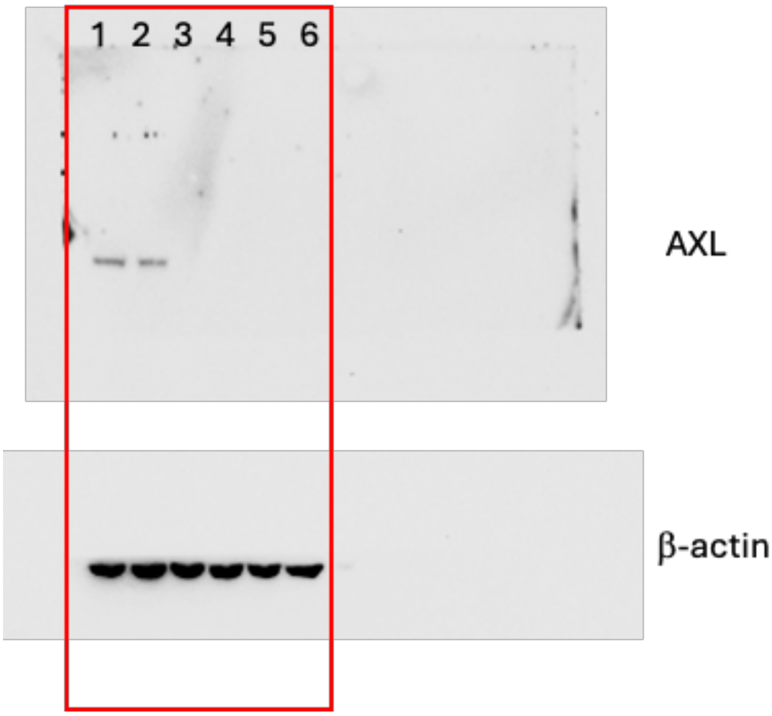

1 = siNC Day 4
2 = siNC Day
3 = siAXL #45 Day 4
4 = siAXL #45 Day 7
5 = siAXL #47 Day 4
6 = siAXL #47 Day 7

Suppl. Fig S8:

Suppl. Fig. S8A:

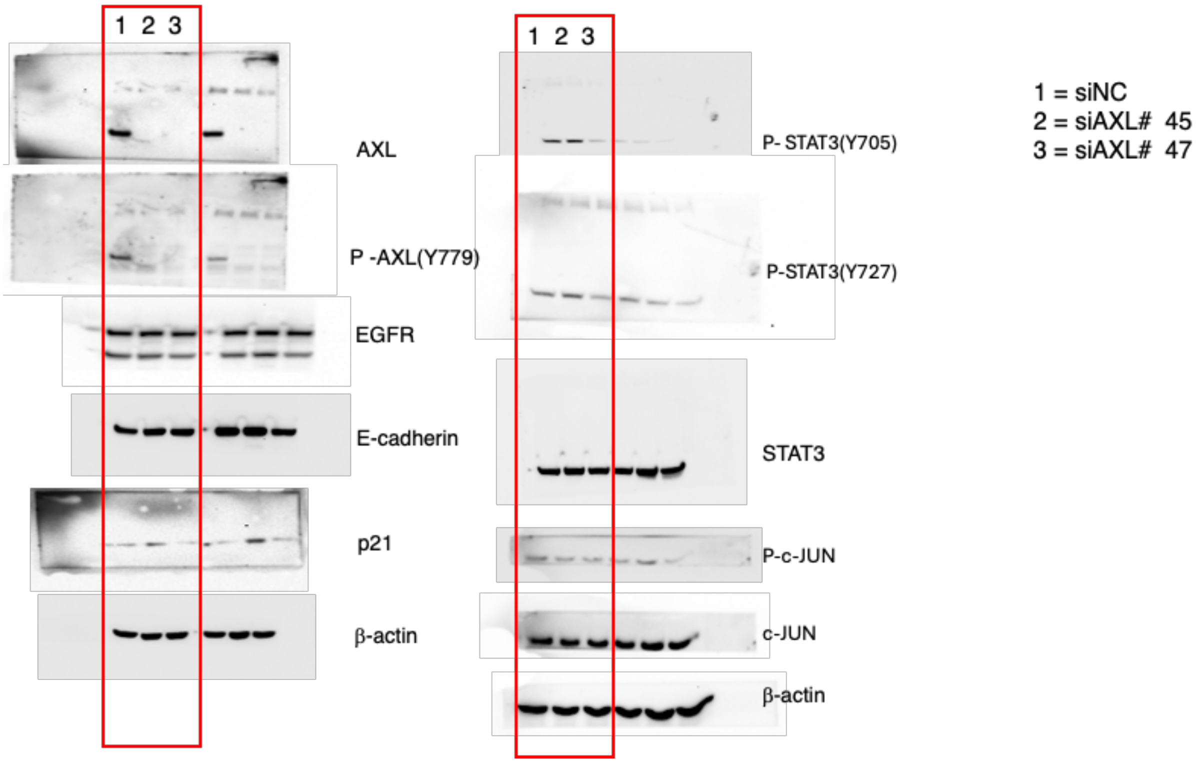

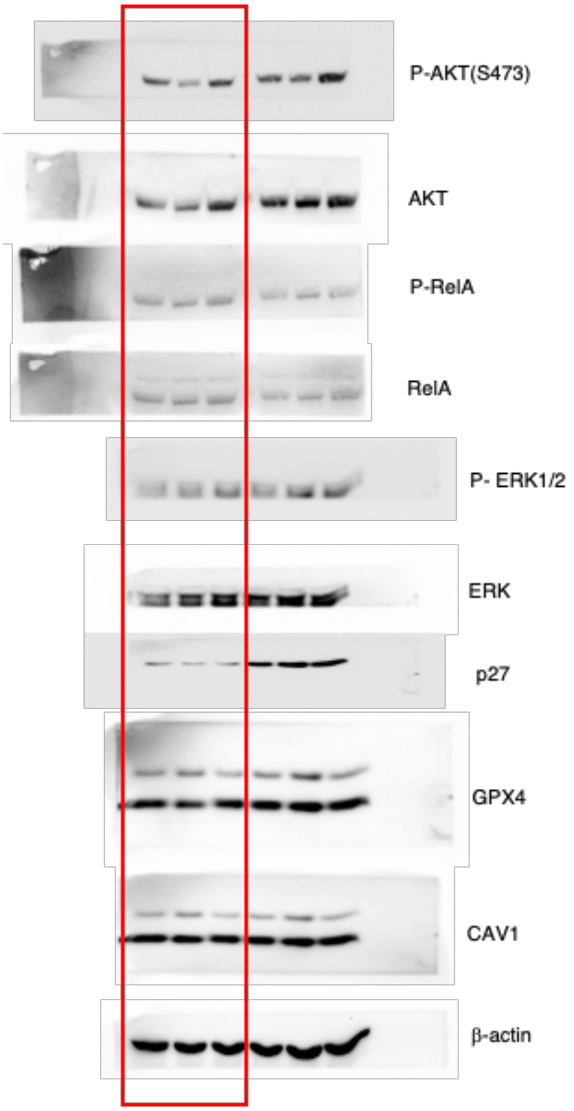

1 = siNC
2 = siAXL# 45
3 = siAXL# 47

Suppl. Fig S8B:

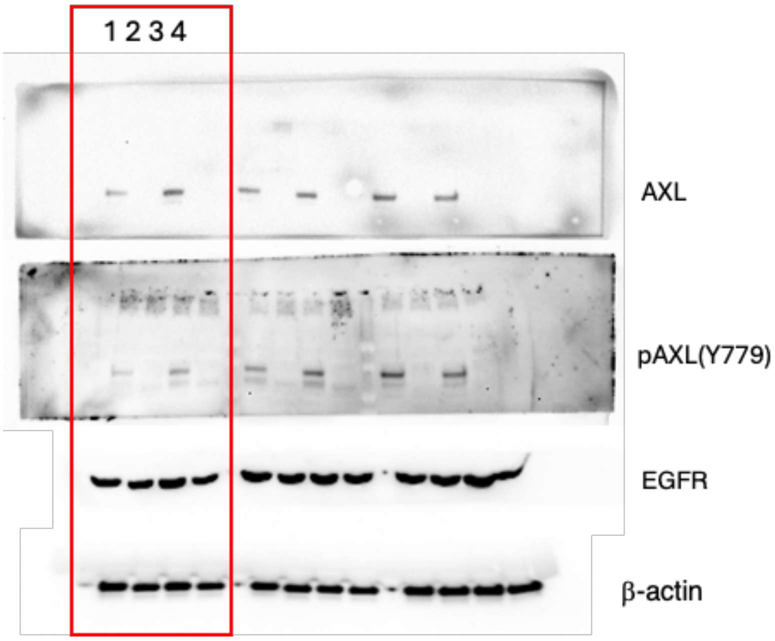

1 = siNC+ DMSO
2 = siAXL #47 + DMSO
3 = siNC+ SF
4 = siAXL #47 + SF

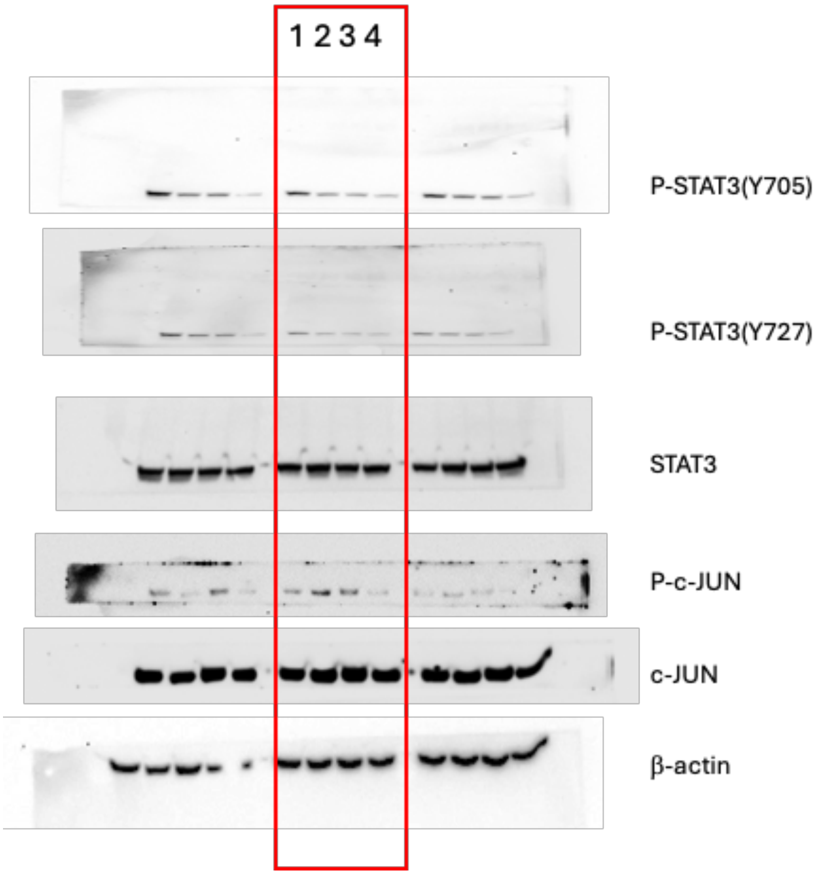

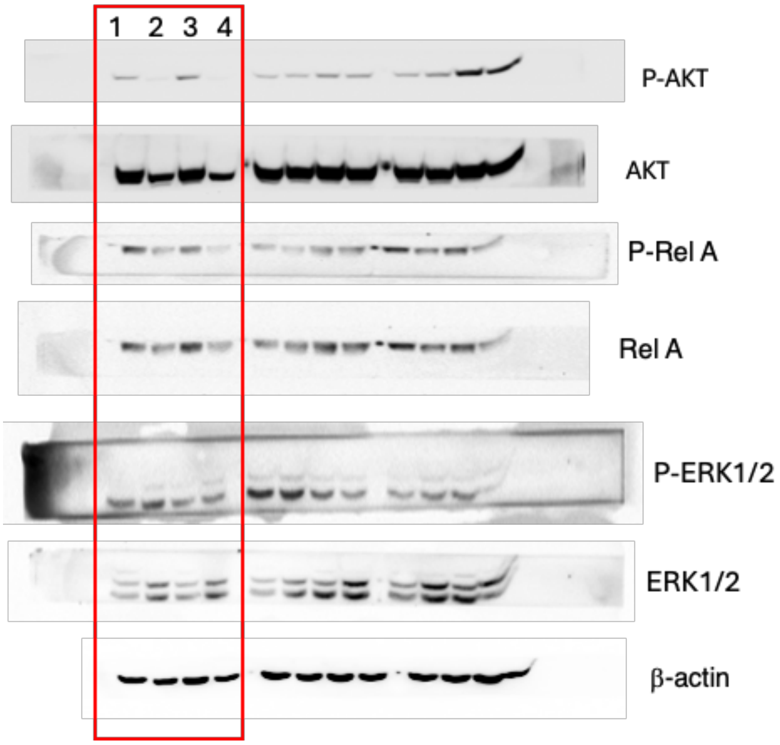

1 = siNC+ DMSO
2 = siAXL #47 + DMSO
3 = siNC+ SF
4 = siAXL #47 + SF

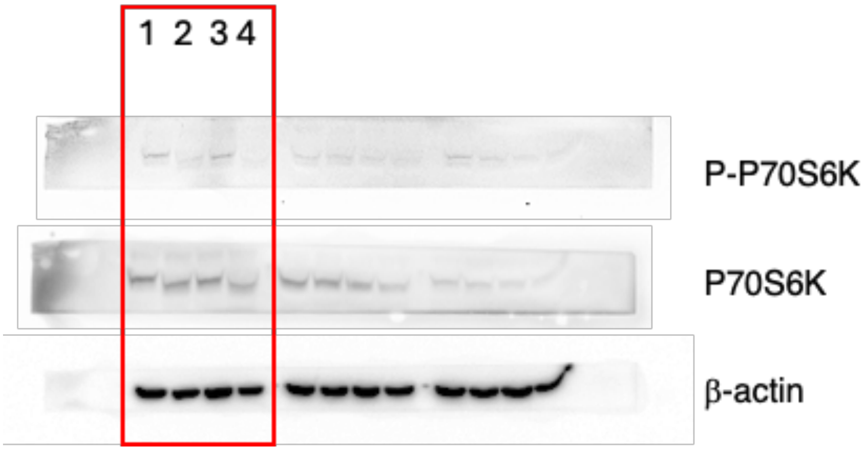

Suppl. Fig. S8C:

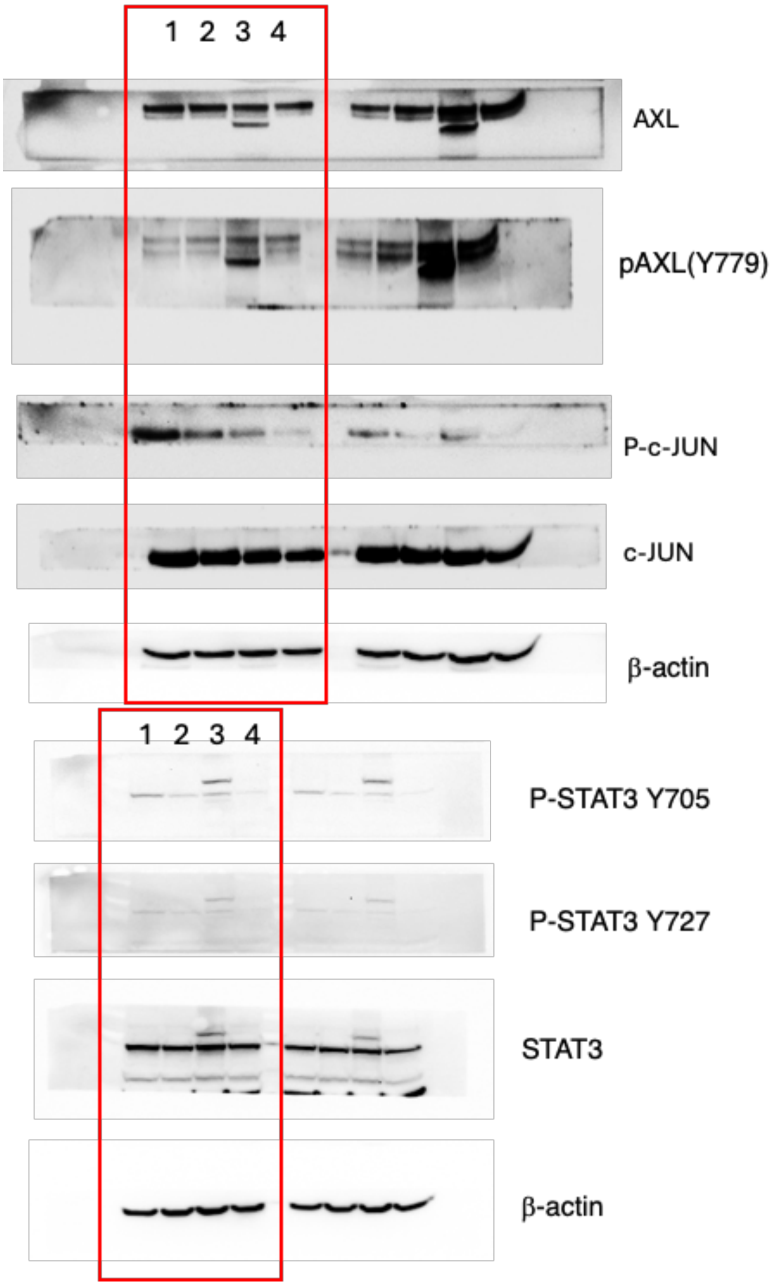

1 = DMSO
2 = SF
3 = BGB324
4 = SF+BGB324

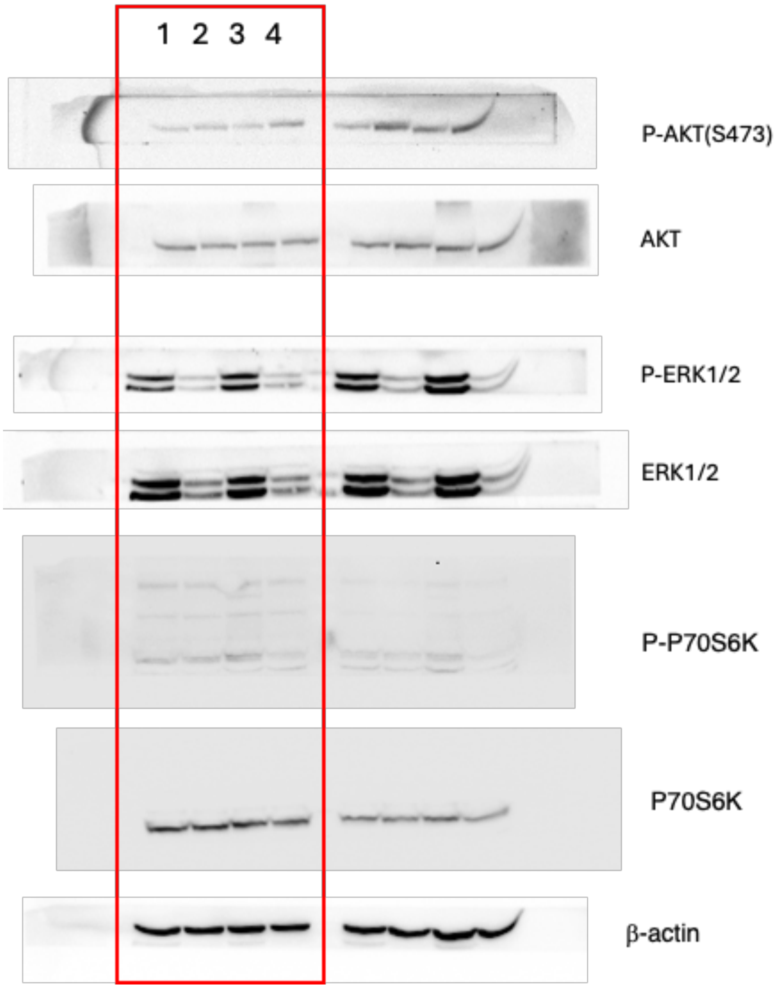

1 = DMSO
2 = SF
3 = BGB324
4 = SF+BGB324

Suppl. Fig. S10:

Suppl. Fig. S10A:

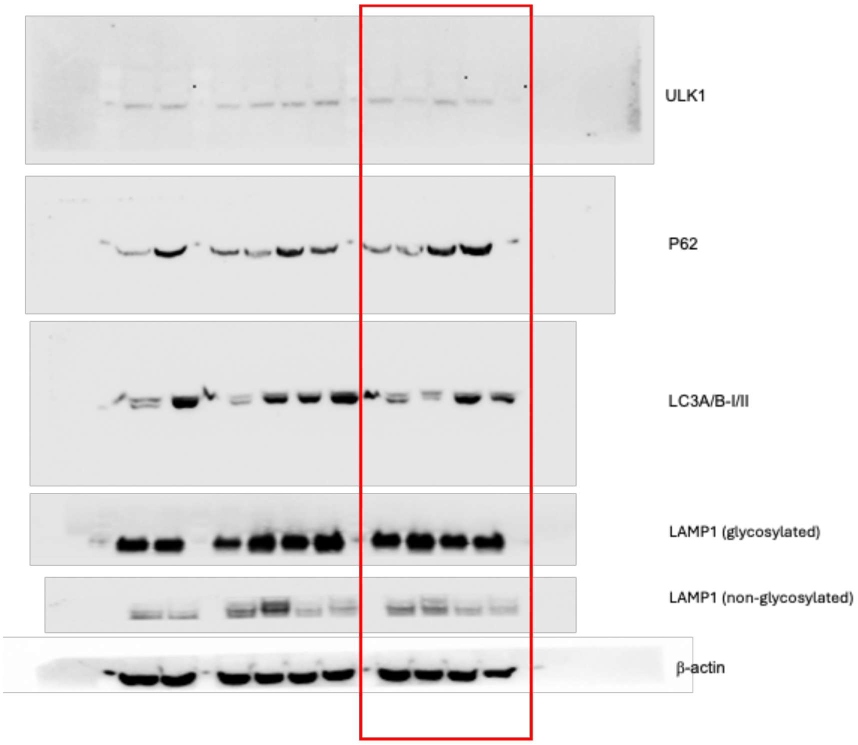

1 = siNC, basal
2 = siCAV1 #2, basal
3 = siNC + CQ
4 = siCAV1 #2 + CQ

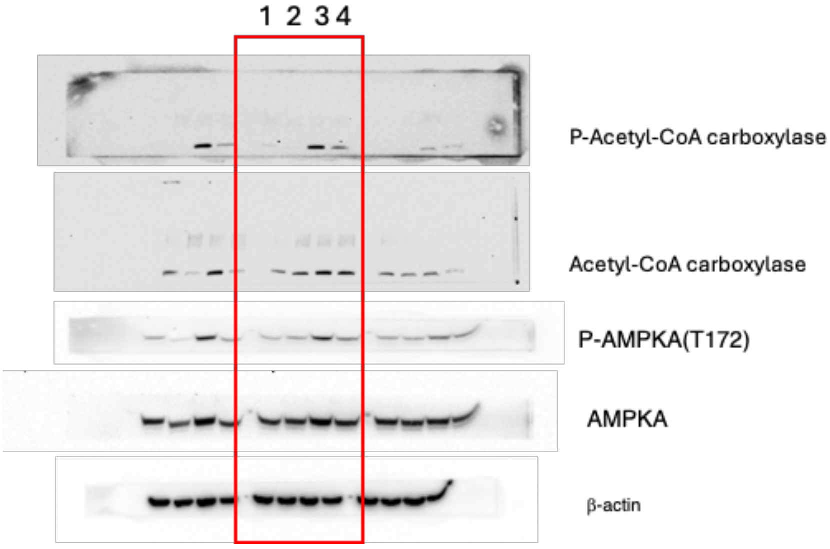

1 = siNC + DMSO
2 = siAXL #47 + DMSO
3 = siNC + SF
4 = siAXL #47 + SF

Suppl. Fig. S10B:

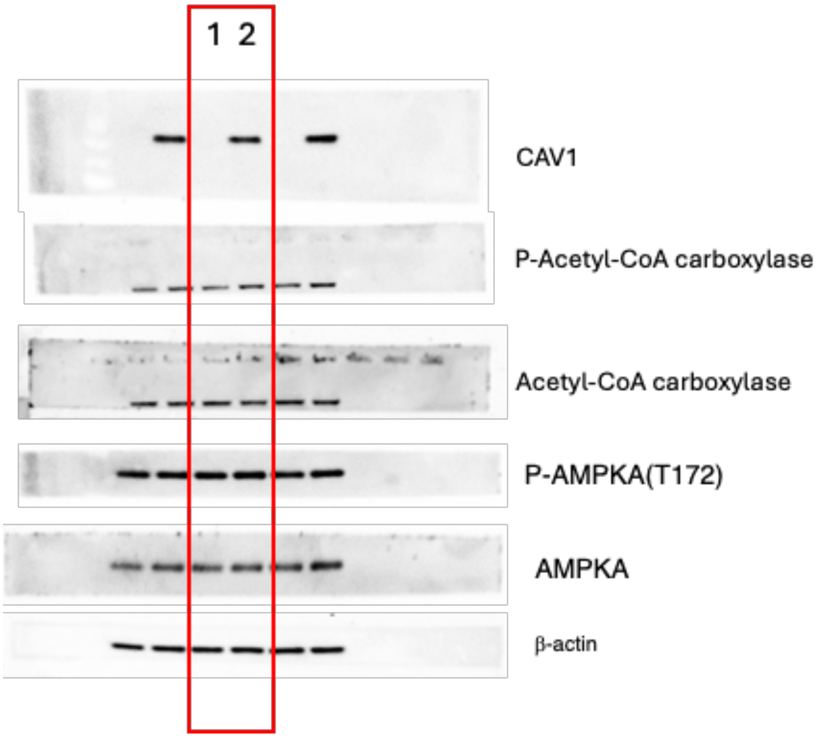

1 = Huh-7-EV
2 = Huh-7-CAV1-OE

Suppl. Fig. S10E:

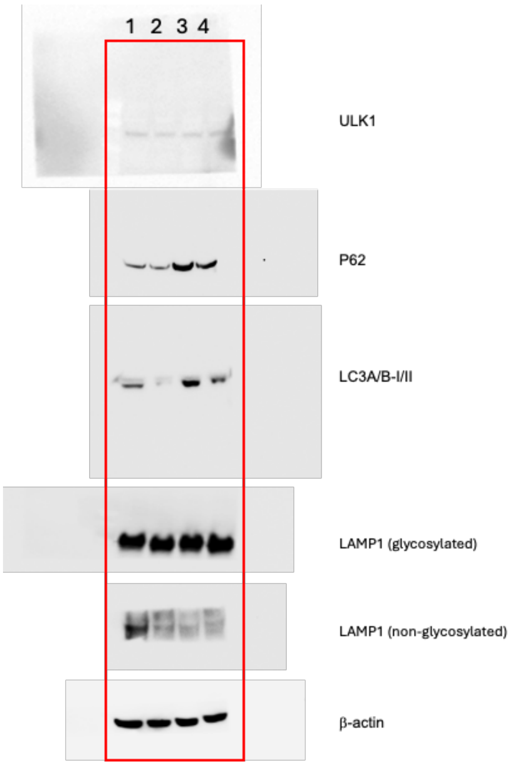

1 = siNC (basal)
2 = siAXL #47 (Basal)
3 = siNC + CQ
4 = siAXL #47 + CQ

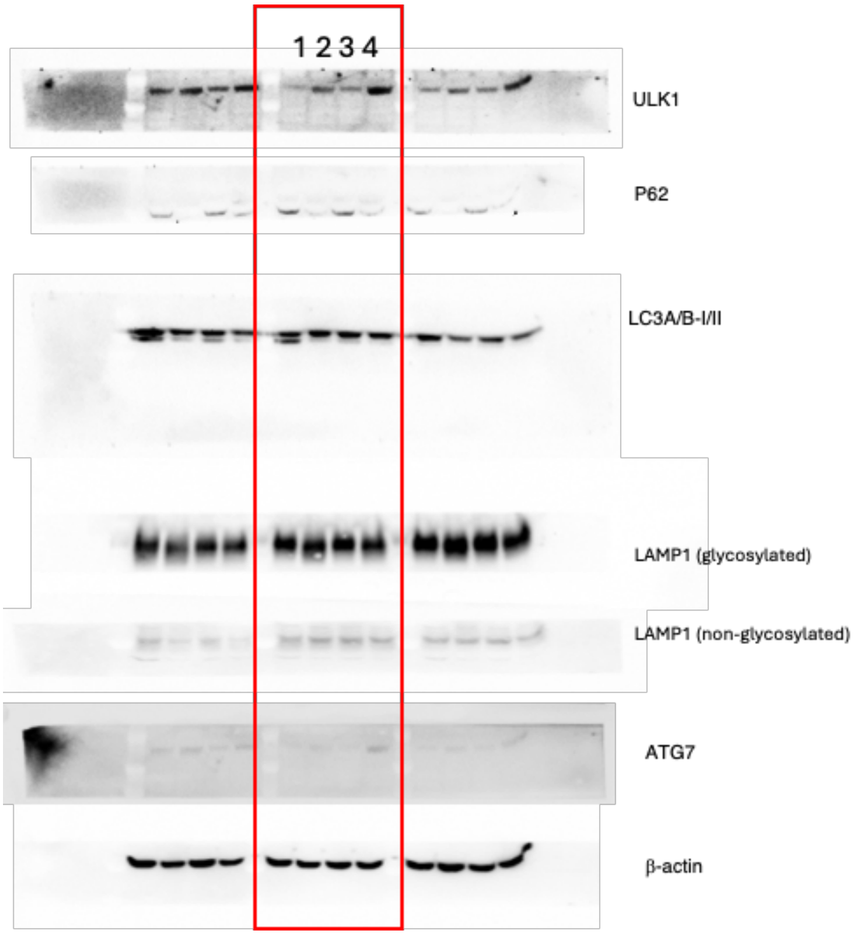

1 = siNC + DMSO
2 = siAXL + DMSO
3 = siNC + SF
4 = siAXL + SF

Suppl. Fig S10 G:

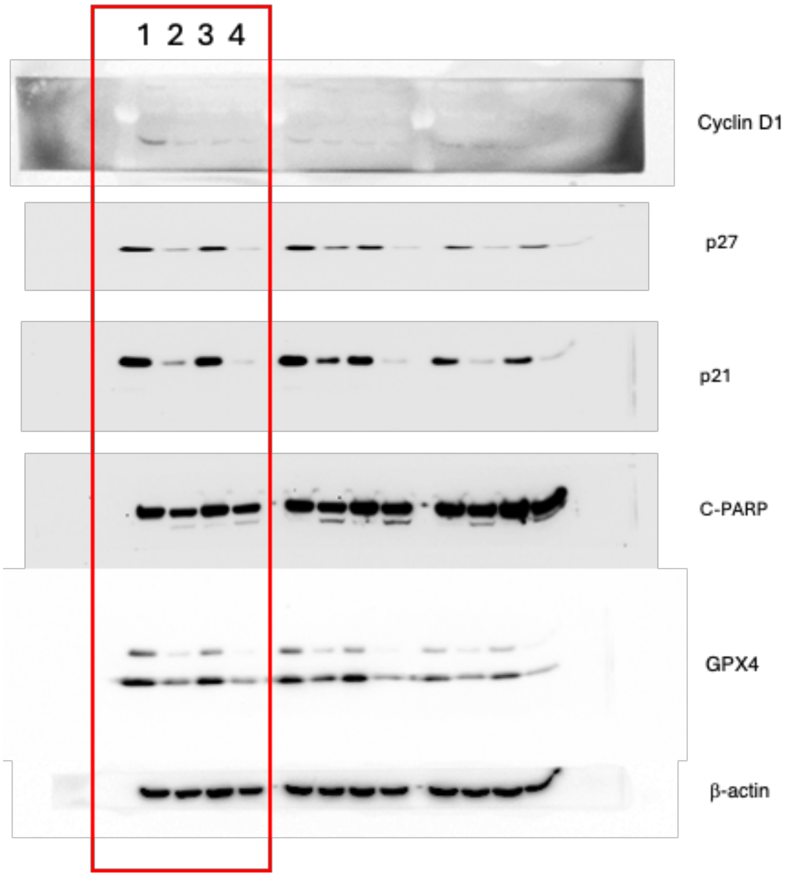

1 = siNC + DMSO
2 = siAXL + DMSO
3 = siNC + SF
4 = siAXL + SF

Suppl. Fig. S10 H:

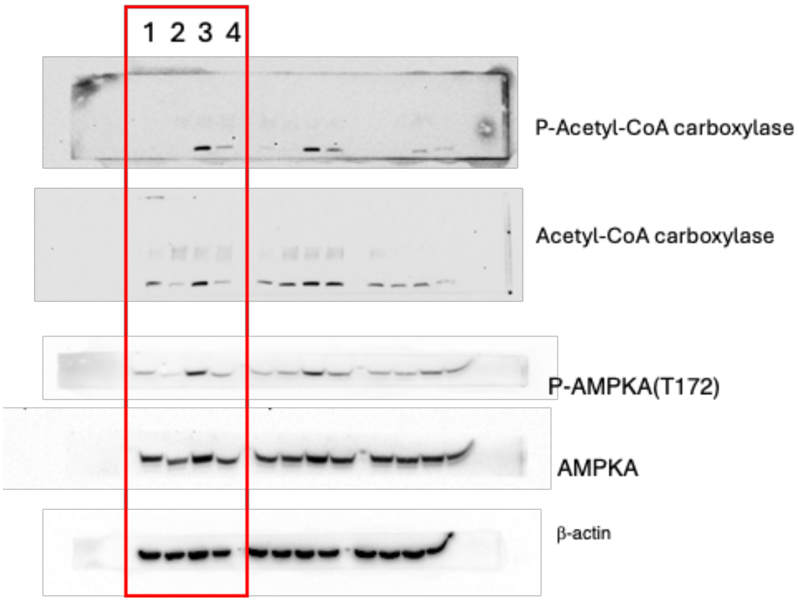

1 = siNC + DMSO
2 = siAXL + DMSO
3 = siNC + SF
4 = siAXL + SF

Suppl. Fig. S12:

Suppl. Fig. S12D:

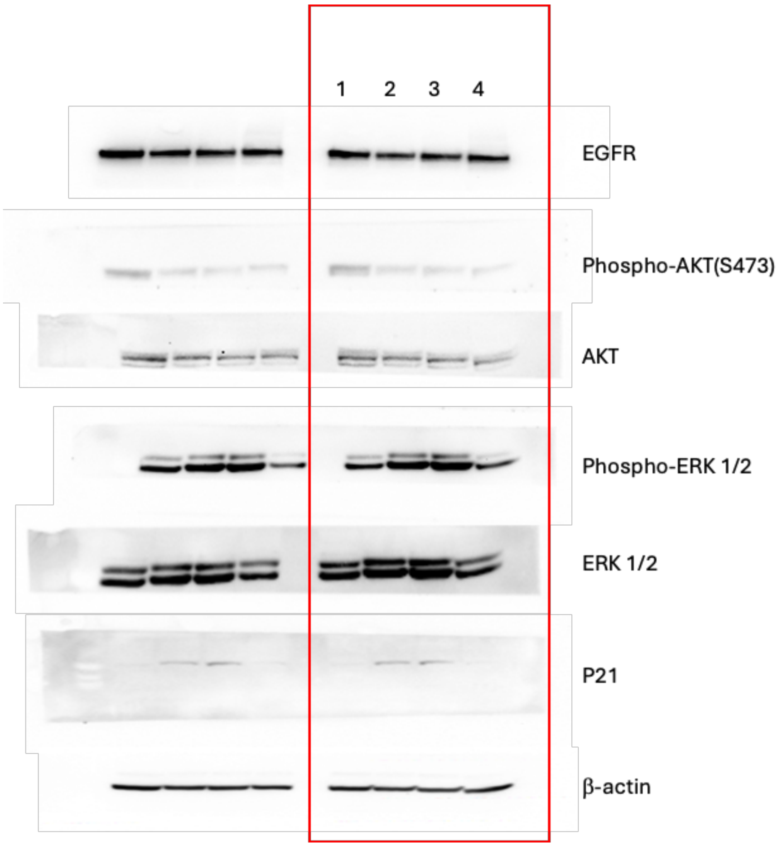

1 = Cy3-miRNC
2 = miR-7 (Ambion)
3= Cy3-labelled passenger miR-7
4 = Double labelled miR-7

Suppl. Fig. S12E:

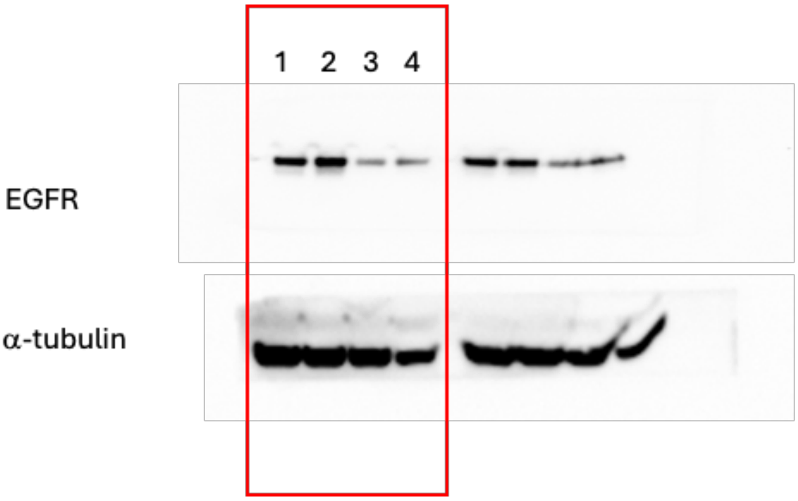

1 = miR-NC Day 4
2 = miR-NC Day 7
3 = miR-7 Day 4
4 = miR-7 Day 7

Fig 6:

Fig. 6H:

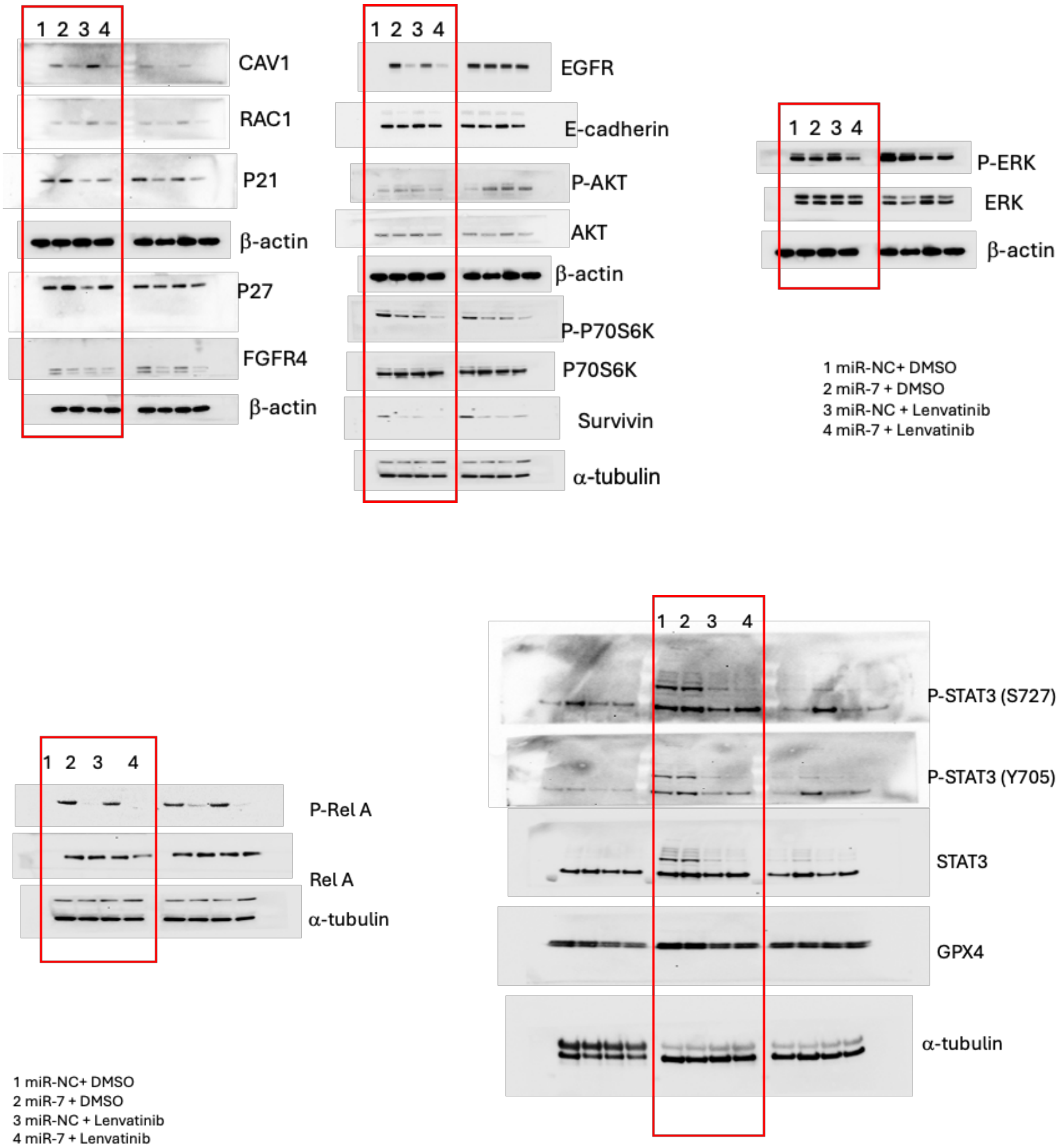
1 2 3 4
CAV1
RAC1
P21
β-actin
P27
FGFR4
β-actin
1 2 3 4
EGFR
E-cadherin
P-AKT
AKT
β-actin
P-P70S6K
P70S6K
Survivin
α-tubulin
1 2 3 4
P-ERK
ERK
β-actin
1 miR-NC+ DMSO
2 miR-7 + DMSO
3 miR-NC + Lenvatinib
4 miR-7 + Lenvatinib
1 2 3 4
P-Rel A
Rel A
α-tubulin
1 miR-NC+ DMSO
2 miR-7 + DMSO
3 miR-NC + Lenvatinib
4 miR-7 + Lenvatinib
1 2 3 4
P-STAT3 (S727)
P-STAT3 (Y705)
STAT3
GPX4
α-tubulin

Suppl. Fig. S13:

Suppl. Fig. S13F:

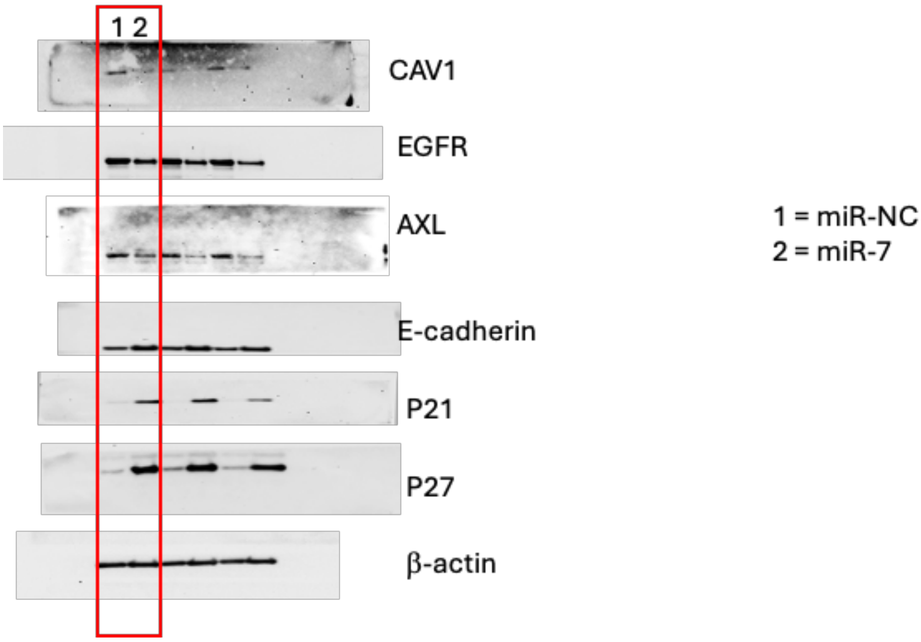

Suppl. Fig. S13G:

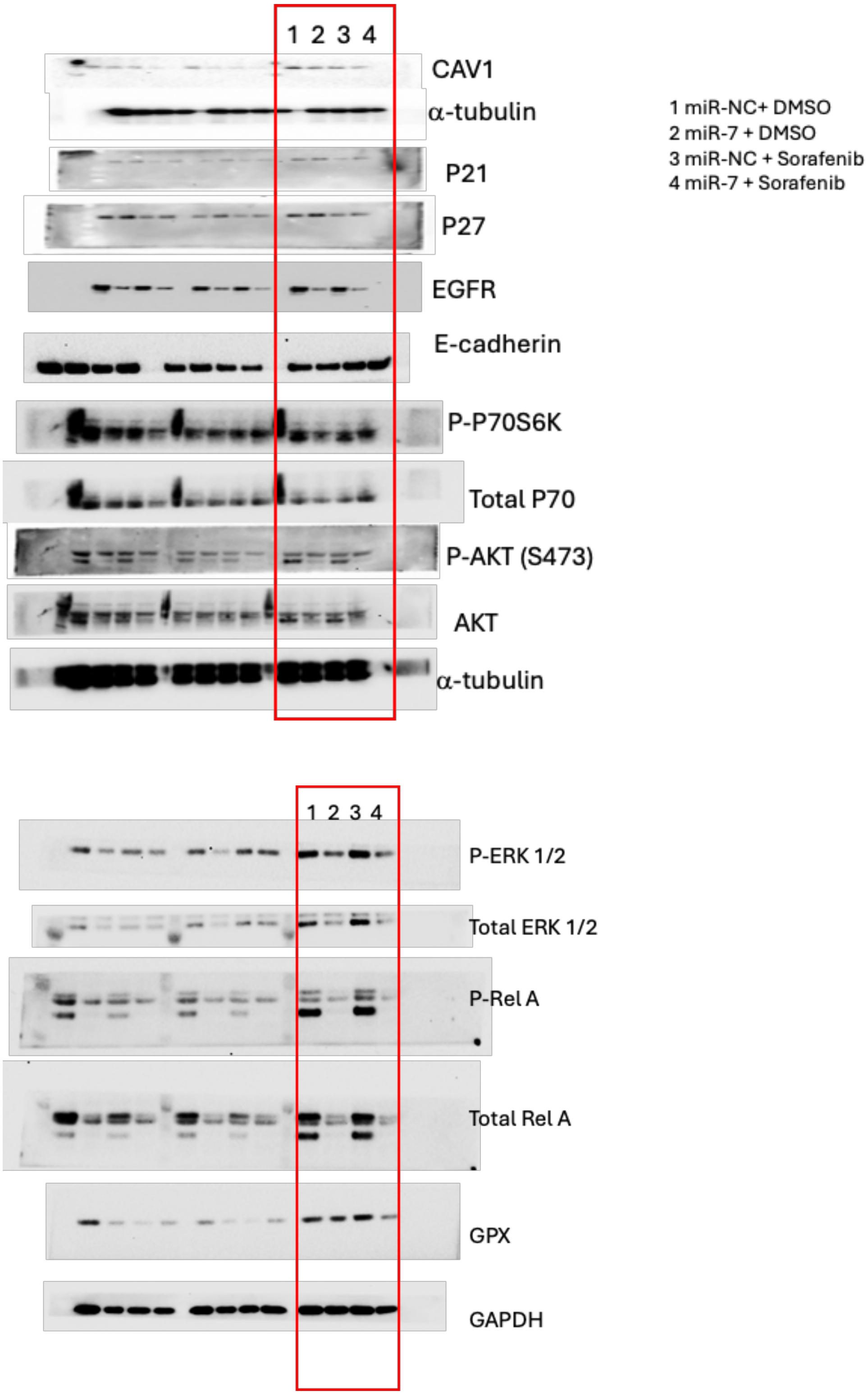

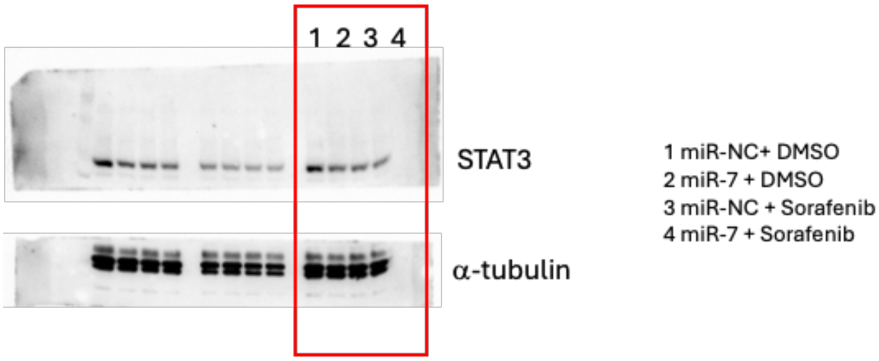
1 2 3 4
STAT3
α-tubulin
1 miR-NC+ DMSO
2 miR-7 + DMSO
3 miR-NC + Sorafenib
4 miR-7 + Sorafenib

Suppl. Fig. S14:

Suppl. Fig. S14A:

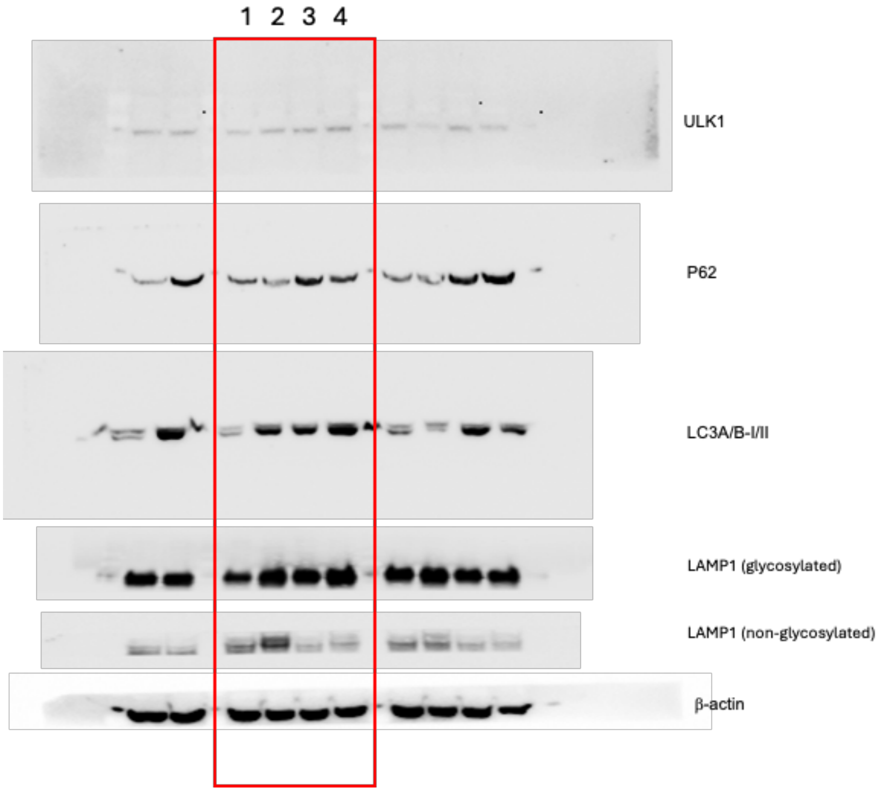

1 = miR-NC, basal
2 = miR-7, basal
3 = miR-NC + CQ
4 = miR-7 + CQ

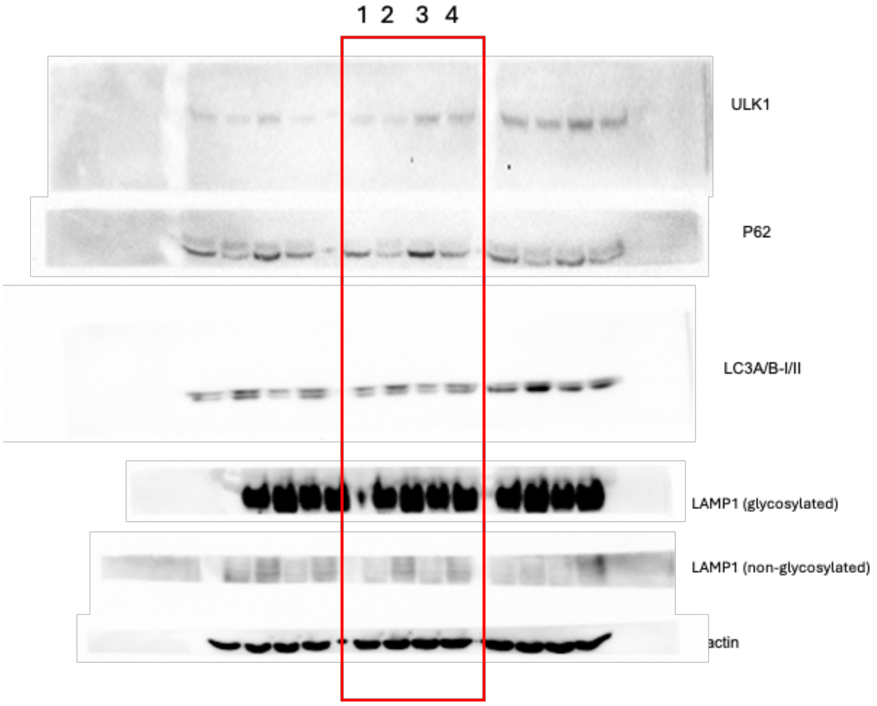

1 = miR-NC + DMSO
2 = miR-7 + DMSO
3 = miR-NC + SF
4 = miR-7 + SF

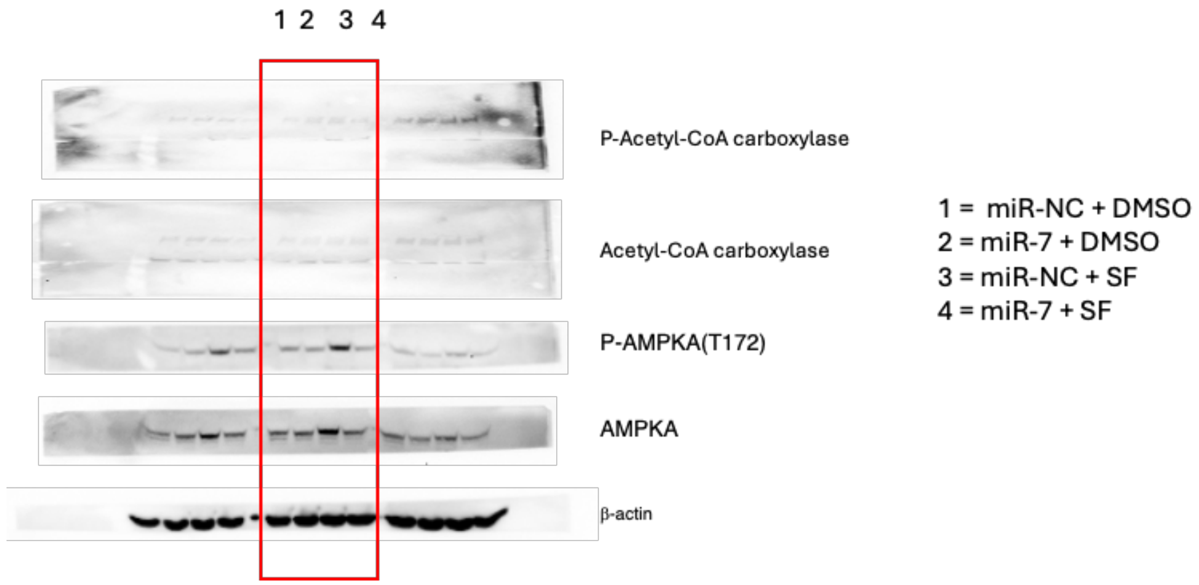
1 2 3 4
P-Acetyl-CoA carboxylase
Acetyl-CoA carboxylase
P-AMPKA(T172)
AMPKA
β-actin
1 = miR-NC + DMSO
2 = miR-7 + DMSO
3 = miR-NC + SF
4 = miR-7 + SF

Suppl. Fig. S15:

Suppl. Fig. S15A:

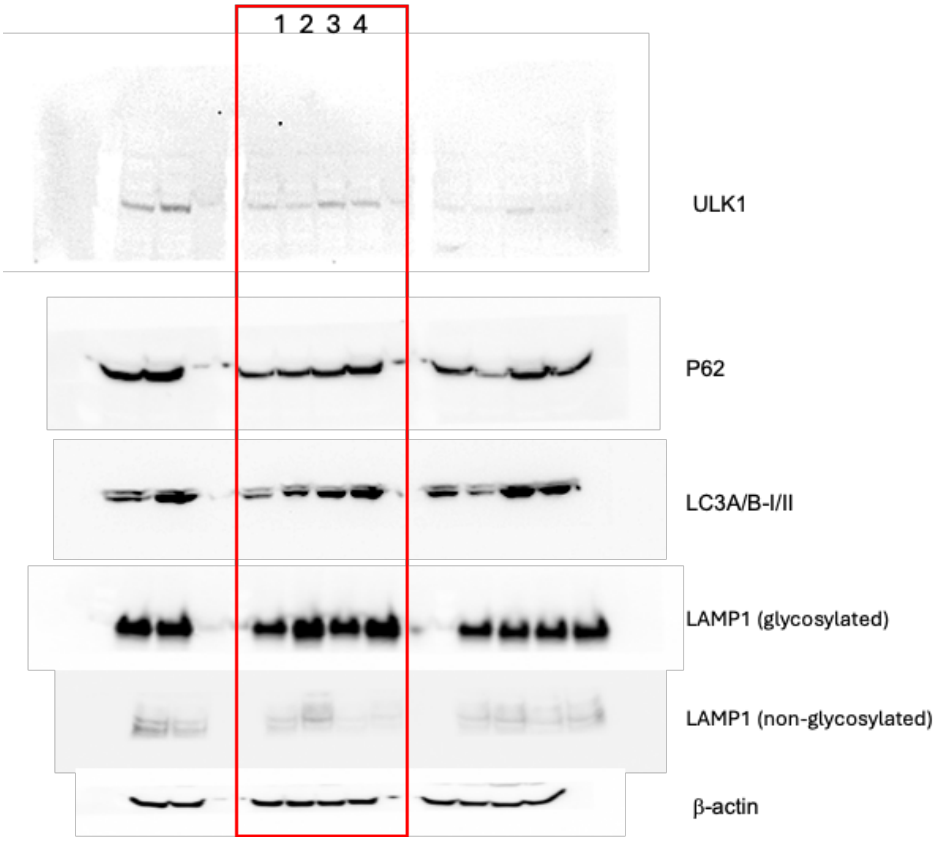

Huh-7/LR cells:

1 = miR-NC (basal)
2 = miR-7 (basal)
3 = miR-NC + CQ
4 = miR-7 + CQ

Fig. 7:

Fig. 7M:

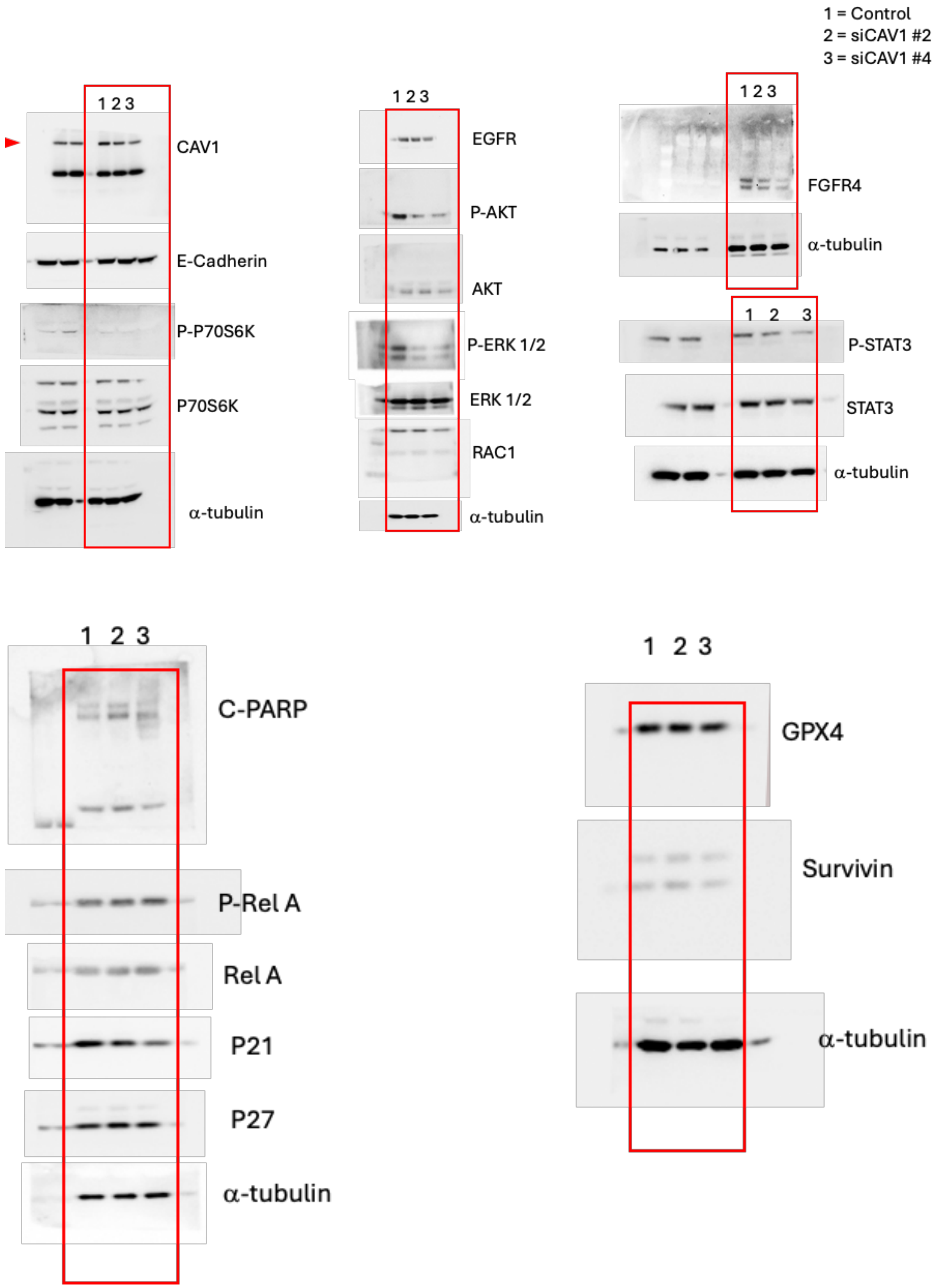
1 = Control
2 = siCAV1 #2
3 = siCAV1 #4
1 2 3
CAV1
E-Cadherin
P-P70S6K
P70S6K
α-tubulin
1 2 3
EGFR
P-AKT
AKT
P-ERK 1/2
ERK 1/2
RAC1
α-tubulin
1 2 3
FGFR4
α-tubulin
1 2 3
P-STAT3
STAT3
α-tubulin
1 2 3
C-PARP
P-Rel A
Rel A
P21
P27
α-tubulin
1 2 3
GPX4
Survivin
α-tubulin

Suppl. Fig. S16:

Suppl. Fig, S16I:

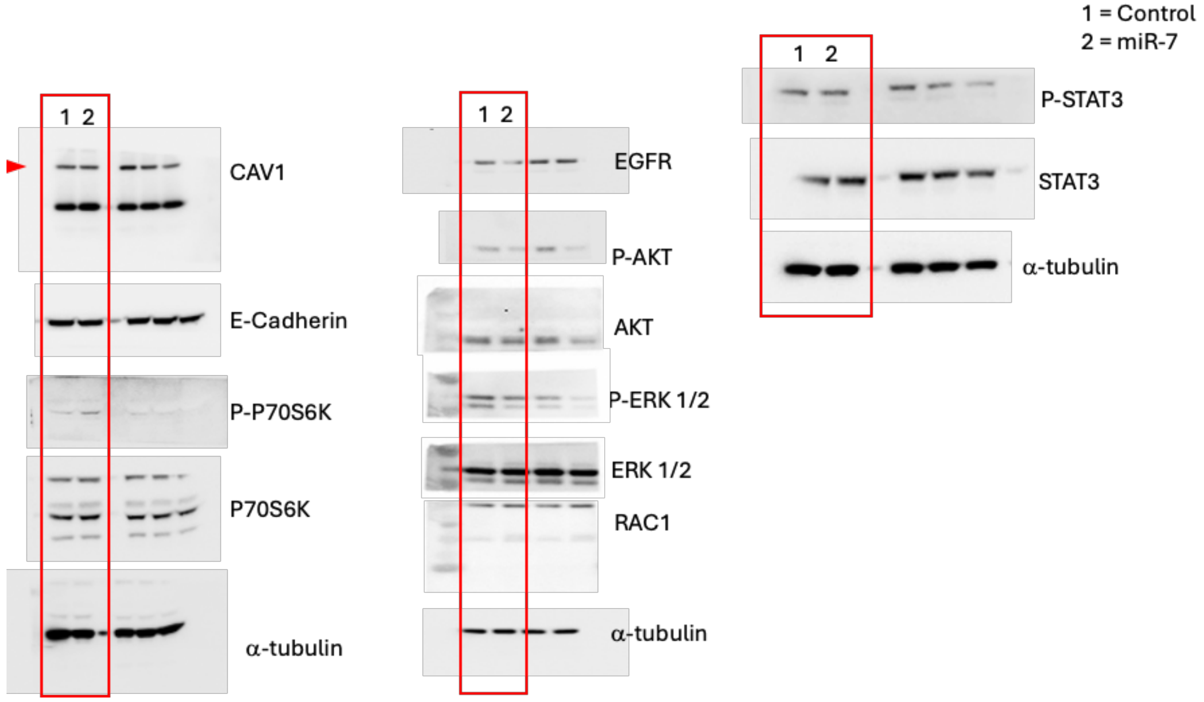

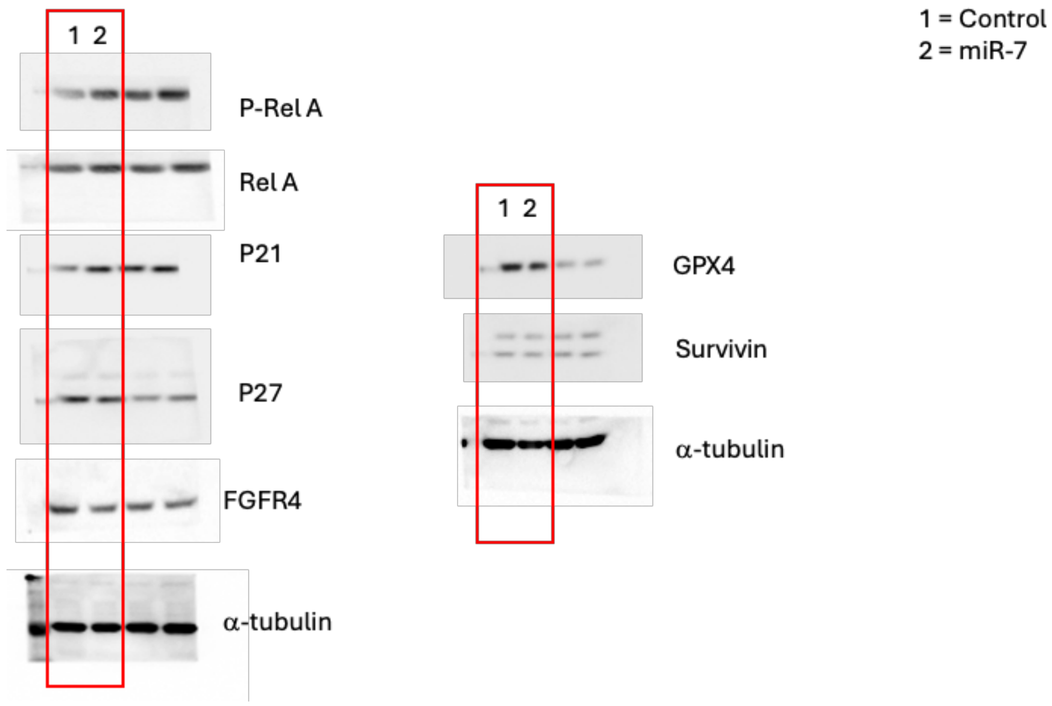

Supplement: Supplementary file 3 — uncropped membranes [file 41419_2025_7887_MOESM3_ESM.pdf]
